# Temporal Trend Analysis of Atrial Fibrillation/Flutter Disease Burden in High-Income Countries Between 1990 and 2021

**DOI:** 10.31083/RCM36427

**Published:** 2025-07-25

**Authors:** Yuqiao Pang, Hui Li, Yuyao Zhang, Xiaoting Sun, Hong Li, Yi Liang, Xuejing Song, Lizhi Zhao

**Affiliations:** ^1^Department of Cardiology, The Affiliated Traditional Chinese Medicine Hospital, Southwest Medical University, 646000 Luzhou, Sichuan, China; ^2^The Second Affiliated Hospital of Guangzhou University of Chinese Medicine, 510120 Guangzhou, Guangdong, China; ^3^Department of Cardiology, Chongqing Traditional Chinese Medicine Hospital, 400000 Chongqing, China; ^4^Depatment of Endocrinology, Hospital of Chengdu University of Traditional Chinese Medicine, 610075 Chengdu, Sichuan, China; ^5^Department of Nephrology, Luzhou Hospital of Traditional Chinese Medicine, 646000 Luzhou, Sichuan, China

**Keywords:** atrial fibrillation/atrial flutter, the Global Burden of Disease Study, age-period-cohort (APC) models

## Abstract

**Background:**

Atrial fibrillation and atrial flutter (AF/AFL) disease is a common arrhythmia that poses a significant health risk to the older population. With an aging population worldwide, the incidence and mortality rates of AF/AFL show notable gender differences, presenting a challenge to public health systems. This study focused on the AF/AFL disease burden trends in high-income European Union 15+ (EU15+) countries.

**Methods:**

Data were sourced from the Global Burden of Disease Study (GBD 2021), using age-standardized incidence rates (ASIRs) and age-standardized mortality rates (ASMRs) by gender for each year from 1990 to 2021 in EU15+ countries; the mortality-incidence index (MII) was calculated. Analyses were conducted using Joinpoint regression software and age–period–cohort (APC) models to evaluate trends in morbidity, annual changes in morbidity (net drift), annual percentage changes in age-specific morbidity (local drift), and period- and cohort-relative risks by gender, allowing the impact of age, period, and cohort effects on morbidity trends to be assessed.

**Results:**

The study found a declining trend in AF/AFL ASIRs and ASMRs in most EU15+ countries, though significant differences were observed between countries. Male ASIRs and ASMRs were generally higher than those of females, though older women often had higher incidence and mortality rates than men. Furthermore, advances in treatment methods, such as updated anticoagulation therapy, radiofrequency ablation, and novel rhythm control drugs, have impacted the changes in disease burden.

**Conclusions:**

Although the AF/AFL disease burden has declined in more than half of the high-income EU15+ countries, there are significant differences in trend changes between countries. This decline may be due to advances in treatment, such as newer anticoagulation therapies, radiofrequency ablation techniques, and the use of novel cardioverter drugs. Trend changes with unique characteristics may be related to the healthcare system of each country, socioeconomic factors, and the promotion of health education. This study also identified gender differences, with older women at greater risk of developing AF/AFL, implying that the older female population faces the need for enhanced risk assessment and management.

## 1. Introduction

Atrial fibrillation and atrial flutter (AF/AFL), prevalent cardiac arrhythmias globally, significantly contribute to elevated morbidity and mortality, particularly among aging populations [1]. Against the backdrop of global demographic aging, AF/AFL epidemiology exhibits pronounced sex-specific disparities in prevalence, incidence, and mortality, creating substantial challenges for healthcare infrastructure [2]. While existing literature predominantly emphasizes cross-national disease burden comparisons [3], granular analyses of high-income nations remain limited. These countries demonstrate distinct advantages in healthcare resource allocation, therapeutic innovation, and socioeconomic determinants, potentially driving divergent burden trajectories compared to other regions [4]. This study aims to explore the trends in the disease burden of AF/AFL in high-income countries by analyzing the age-standardized incidence rate (ASIR), age-standardized mortality rate (ASMR), and mortality-incidence index (MII) between 1990 and 2021 in European Union 15+ (EU15+) countries. The study also seeks to uncover gender differences, age effects, and diverse trends across countries. Concurrently, the progress in treatment methods for AF/AFL, such as anticoagulation therapy, radiofrequency ablation, and novel rhythm control drugs (e.g., dronedarone, vernakalant), and their implementation and dissemination in different countries, will be examined to assess their impact on disease burden trends [5, 6, 7]. Through an in-depth analysis of gender differences and trends in the incidence and mortality of AF/AFL in high-income countries, this study aims to provide evidence for the development of more targeted public health policies and optimized disease management strategies.

## 2. Materials and Methods

### 2.1 Data Sources

The present study utilizes data sourced from the Global Burden of Disease Study 2021 (GBD 2021; https://vizhub.healthdata.org/gbd-results/). GBD 2021 provides systematic health estimates for 369 diseases and injuries and 87 risk factors across 204 countries and regions. These estimates are primarily based on methods such as the Cause of Death Ensemble Modeling (CODEm), Spatiotemporal Gaussian Process Regression (ST-GPR), and the Bayesian meta-regression tool DisMod-MR, which estimate age-specific incidence rates, mortality rates, disability-adjusted life years (DALYs), and demographic data for each country, stratified by cause, age, sex, year, and location [8]. Compared to GBD 2019, GBD 2021 incorporates an additional 147 surveys, 21 censuses, and 634 country-year life and sample registration data, bringing the total to 1455 surveys and censuses and 8709 country-year life and sample registration datasets, along with 150 other sources. Methodological enhancements feature temporal weighting optimization in ST-GPR via beta density functions, improving trend estimation accuracy through enhanced data representativeness [9]. AF/AFL is categorized as a Level 3 cause within the broader Level 2 cause category of Cardiovascular Disease, which is a subclassification of Level 1 non-communicable diseases [10]. According to the 2021 Disease Classification, non-fatal causes and injuries from AF/AFL diagnosed by electrocardiogram are mapped to the corresponding International Classification of Diseases (ICD) codes in GBD 2021, which are 472.3–427.32 (ICD-9) and 148–148.92 (ICD-10) [11]. AF/AFL in GBD 2021 is defined as ECG-confirmed arrhythmia meeting all three criteria:Irregular RR intervals; Absence of distinct P waves; Atrial cycle length <200 ms when visible. Cases represent unique individuals with incident or prevalent AF/AFL within a calendar year, excluding duplicate records from multiple healthcare encounters [12]. GBD evaluates the availability and completeness of Vital registration and verbal autopsy data by reporting the percentage of total deaths from specific causes in a given location and year, excluding sources with completeness below 50%. The quality of mortality data is also assessed based on the availability and completeness of life registration and verbal autopsy data, with ratings ranging from 0 to 5 stars. A higher star rating indicates better completeness, availability, and fewer “garbage codes” (i.e., deaths assigned to ambiguous diagnoses or conditions that cannot be potential causes of death according to ICD-10 classification) [13]. This study assesses the quality of available mortality data for 19 EU15+ countries during the period 1990–2020, with 15 countries receiving a 5-star rating, indicating that 85%–100% of their mortality data are well-validated. The remaining four countries (Belgium, France, Greece, and Luxembourg) received a 4-star rating, signifying that 65%–84% of their data are well-validated [13]. These 19 countries have been used in previous observational reports [14, 15] because of their similarities in health expenditure and comparability.

### 2.2 Data Processing

The data on ASIR and ASMR per 100,000 population for EU15+ countries from 1990 to 2021 were extracted from the GBD Results Tool. These age-standardized rates were derived using a standard population based on the unweighted average of populations across all countries for each 5-year age group, as defined in the United Nations World Population Prospects (2012 Revision) [16] for 2010–2035. The differences in ASIR and ASMR at the beginning and end of the observation period were calculated for each gender in all 19 countries to facilitate comparisons of absolute and relative changes over time. To quantify the proportion of ASMR to ASIR per 100,000 population among males and females in EU15+ countries from 1990 to 2021, MII was calculated by dividing mortality rates by incidence rates and multiplying by 1000. MII provides a standardized measure to assess disease burden and is commonly used to identify disparities in cancer screening and treatment internationally [17]. By analyzing temporal changes in MII, long-term trends and progress in disease management can be effectively tracked.

### 2.3 Statistical Analysis

Longitudinal trends in AF/AFL disease burden across EU15+ nations were analyzed using Joinpoint Regression Analysis v4.5.0.1 (National Cancer Institute Surveillance Research Program, Bethesda, MD, USA). This methodology identifies temporal inflection points in epidemiological trends by fitting the most parsimonious segmented linear model to log-transformed annual rates. The algorithm iteratively evaluates potential trend breaks through permutation testing with Bonferroni correction, permitting ≤5 significant joinpoints (*p* < 0.05, Monte Carlo-adjusted for multiple comparisons) [18]. Joinpoint regression has been extensively employed in previous cardiovascular disease burden studies to evaluate long-term trend changes [19]. This analysis calculates average percentage change, average annual percentage change (AAPC), and their 95% confidence intervals (CI) to assess temporal trends in AF/AFL morbidity and mortality from 1990 to 2021.

### 2.4 Age-Period-Cohort Analysis

The age-period-cohort (APC) model, a widely used framework in contemporary epidemiology, is built upon the Poisson distribution and allows simultaneous assessment of age, period, and cohort effects on AF/AFL incidence trends. Beyond traditional epidemiological analysis, the APC model aids in uncovering societal changes, disease etiology, aging processes, and demographic dynamics. It has been extensively applied to descriptive epidemiological studies on cardiovascular diseases [20].

The longitudinal age curve represents the relative risk of age groups compared to a reference group, adjusted for period effects. Rate ratios (RR) for periods or cohorts indicate relative risks compared to reference cohorts or periods, accounting for nonlinear period or cohort effects.

We used the GBD 2021 database to estimate the morbidity of AF/AFL as well as demographic data from individual EU15+ countries as inputs to the APC model.To minimize overlap between adjacent cohorts, mortality and population data were divided into consecutive 5-year age groups (1992–2021) and corresponding 5-year birth cohorts. Midpoint years (e.g., 1992–1996 represented by 1994) were used to approximate specific time intervals, resulting in 14 age groups (30–34 to 95–99 years) and 19 birth cohorts (1897–1901 to 1987–1991). The analysis was conducted using the R-based web tool provided by the National Cancer Institute’s Division of Cancer Epidemiology and Genetics (http://analysistools.nci.nih.gov/apc/), which reduces potential confounding and bias. To address limitations of the APC model, stricter selection criteria were applied.Statistical analyses were performed using R software v4.3.1 (R Foundation for Statistical Computing, Vienna, Austria). The significance of estimable functions was evaluated using the Wald χ^2^ test, with two-sided *p*-values < 0.05 indicating statistical significance.

## 3. Results

This study analyzes the statistical changes in the ASIR, ASMR, and MII of AF/AFL in EU15+ countries during the observation period from 1990 to 2021. The ASIR and ASMR data, stratified by gender for each country, are presented in graphical form (per 100,000).

### 3.1 AF/AFL Incidence in 1990 and 2021

**Fig. 1.**
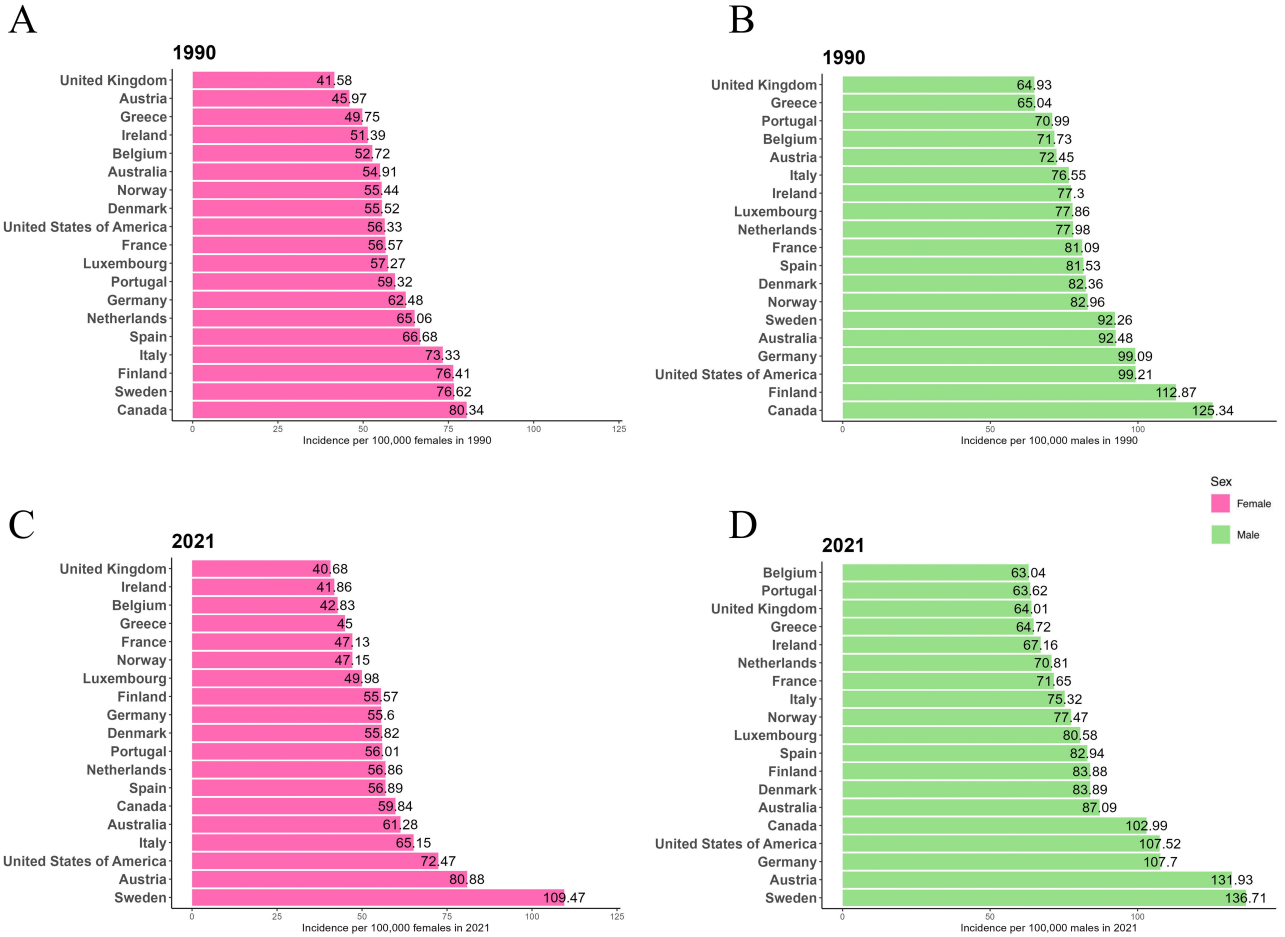
**Age-standardized incidence rates (ASIR) per 100,000 for atrial fibrillation and atrial flutter (AF/AFL) in 1990 and 2021 in European Union 15+ (EU15+) countries**. (A) Female ASIR in 1990. (B) Male ASIR in 1990. (C) Female ASIR in 2021. (D) Male ASIR in 2021.

Fig. 1 shows the ASIR for males and females in EU15+ countries for the years 1990 and 2021. In 1990, Canada had the highest ASIR for both males (125.34/100,000) and females (80.34/100,000). In 2021, Sweden had the highest ASIR for both males (136.71/100,000) and females (109.47/100,000). In 1990, the UK had the lowest ASIR for both males (64.93/100,000) and females (41.58/100,000). By 2021, Belgium had the lowest ASIR for males (63.04/100,000), the UK had the lowest ASIR for females (40.68/100,000), and Belgium’s female ASIR was also among the lowest (42.83/100,000). Throughout both 1990 and 2021, the ASIR for males was consistently higher than that for females in all 19 EU15+ countries.

### 3.2 AF/AFL Mortality in 1990 and 2021

**Fig. 2.**
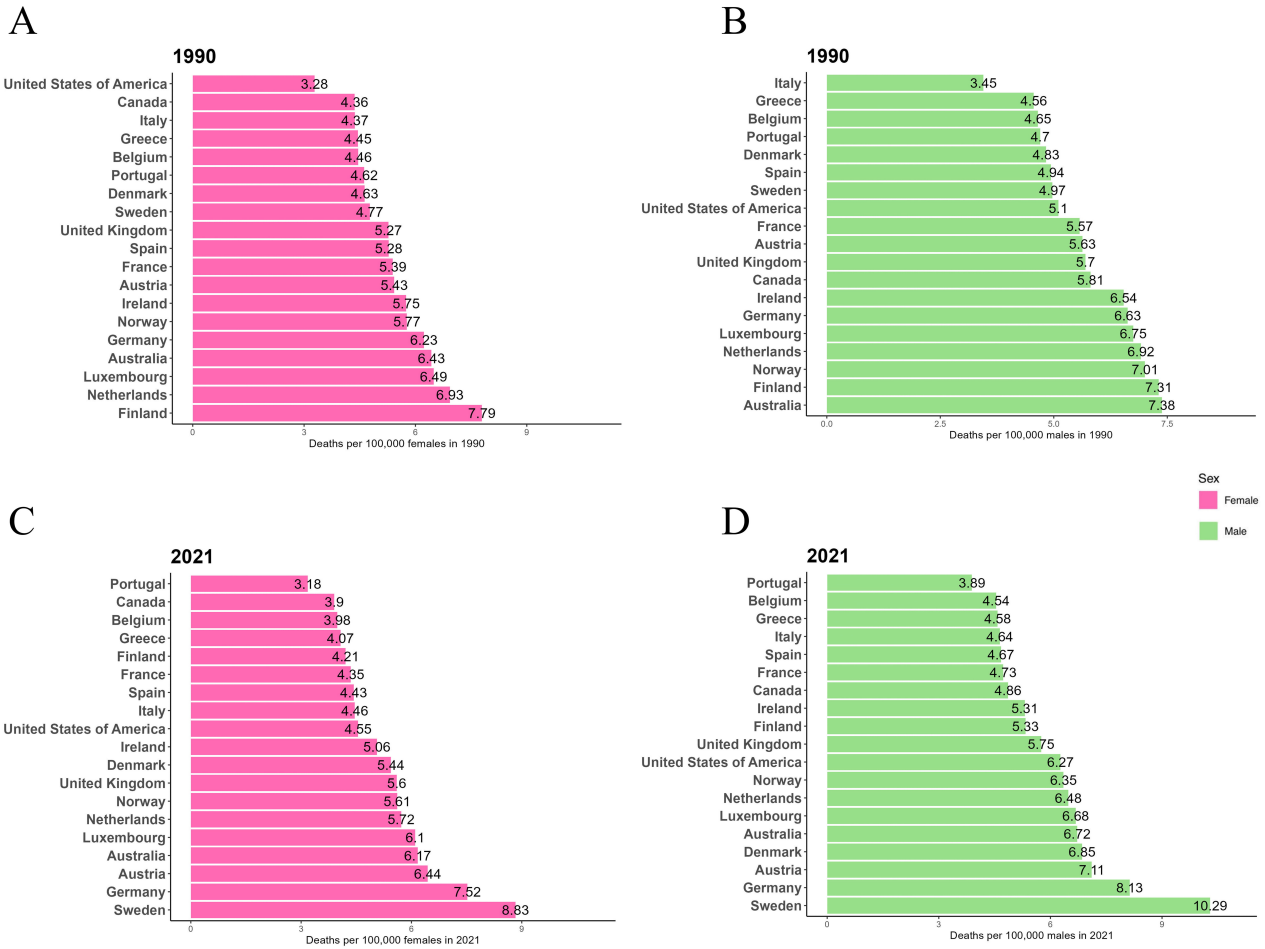
**ASMR per 100,000 for AF/AFL in 1990 and 2021 in EU15+ countries**. (A) Female ASMR in 1990. (B) Male ASMR in 1990. (C) Female ASMR in 2021. (D) Male ASMR in 2021.

Fig. 2 presents the ASMR for males and females in EU15+ countries for 1990 and 2021. In 1990, Australia had the highest ASMR for males (7.38/100,000), while Finland had the highest ASMR for females (7.79/100,000). By 2021, Sweden had the highest ASMR for both males (10.29/100,000) and females (8.83/100,000). In 1990, Italy had the lowest ASMR for males (3.45/100,000), while the USA had the lowest ASMR for females (3.28/100,000). In 2021, Portugal had the lowest ASMR for both males (3.89/100,000) and females (3.18/100,000). In both 1990 and 2021, the ASMR for males was higher than that for females in all 19 EU15+ countries.

### 3.3 AF/AFL Incidence Trend

**Fig. 3.**
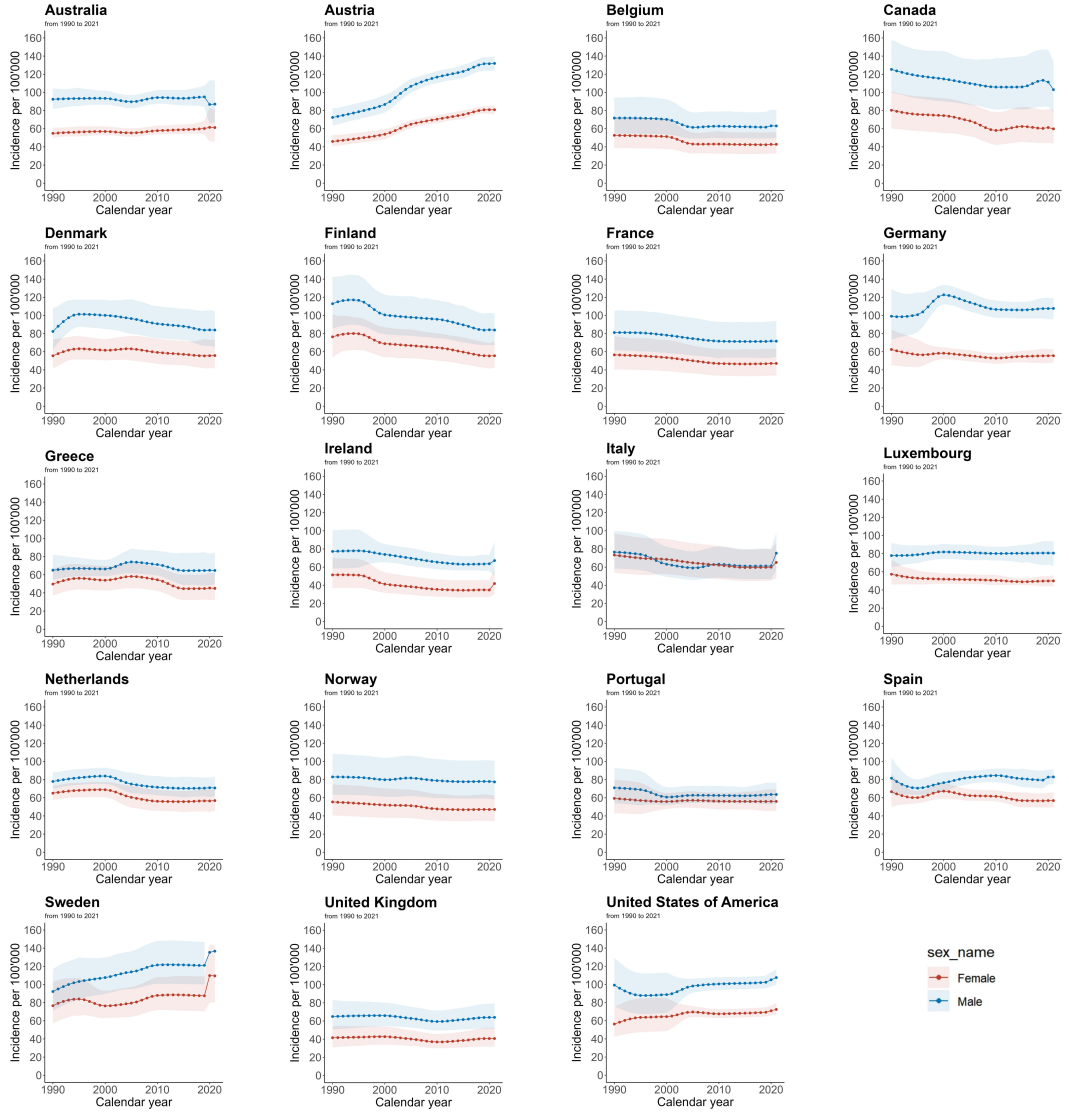
**Trends in age-standardized incidence rates per 100,000 for AF/AFL in EU15+ countries between 1990 and 2021**. Blue squares indicate males and red squares indicate females.

Fig. 3 presents the AF/AFL incidence rate data from 1990 to 2021 for each EU15+ country, disaggregated by gender. Among women, a decrease in ASIR was observed in 14 countries from 1990 to 2021. Finland showed the largest decrease (–27.3%), followed by Canada (–25.5%), while Austria (+75.9%) and Sweden (+42.9%) experienced the greatest increases in ASIR. In men, during the same period, the ASIR decreased in 12 countries, with Finland (–25.7%) and Canada (–17.8%) showing the largest decreases. Austria (+82.1%) and Sweden (+48.2%) saw the largest increases in male ASIR.

### 3.4 Joint Point Analysis of AF/AFL Incidence

Tables 1,2 summarize the joint point analysis results of male and female AF/AFL ASIR from 1990 to 2021, including average percentage change and AAPC for each trend interval. From the AAPC, it is evident that the incidence rates of AF/AFL have generally decreased for women in 15 countries between 1990 and 2021. Finland showed the most significant decrease in incidence (–1.04%, 95% CI: –1.08%, –0.99%), while Austria, Australia, Sweden, and the USA showed increases, with Austria experiencing the largest increase (+1.86%, 95% CI: 1.82%, 1.90%). In men, 12 countries saw a decline in incidence, with Finland showing the largest decrease (–0.95%, 95% CI: –1.02%, –0.88%). Austria, Denmark, Germany, Luxembourg, Spain, Sweden, and the USA experienced increases, with Austria again showing the largest increase (+1.97%, 95% CI: 1.93%, 2.01%). However, the rate of decline varied across countries. According to the average percentage change, Ireland exhibited the fastest decrease in AF/AFL incidence for women between 1996–2000 (–4.9%), while Australia saw the fastest decline in male incidence from 2019–2021 (–5.04%). The most recent trends show that, among the 19 EU15+ countries, 16 countries reported an increase in female AF/AFL incidence, and 15 countries observed an increase in male incidence. The highest increases in incidence from 2019–2021 were observed in Italy for men (+10.25%) and Sweden for women (+13.54%).

**Table 1.** **Joinpoint analysis for AF/AFL disease age-standardized incidence rates in European Union 15+ (EU15+) countries for years 1990–2021 in males**.

Country	Trend1		Trend2		Trend3		Trend4		Trend5		Trend6		AAPC	
	Year	APC (95% CI)	Year	APC (95% CI)	Year	APC (95% CI)	Year	APC (95% CI)	Year	APC (95% CI)	Year	APC (95% CI)	Year	AAPC (95% CI)
Australia	1990–2000	0.10 (0.08 to 0.12)*	2000–2005	–0.94 (–1.00 to –0.88)*	2005–2010	1.14 (1.08 to 1.21)*	2010–2015	–0.25 (–0.31 to –0.18)*	2015–2019	0.40 (0.27 to 0.53)*	2019–2021	–5.04 (–5.68 to –4.41)*	1990–2021	–0.26 (–0.30 to –0.21)*
Austria	1990–2000	1.81 (1.76 to 1.85)*	2000–2005	4.48 (4.35 to 4.61)*	2005–2009	1.87 (1.73 to 2.01)*	2009–2015	1.04 (0.99 to 1.10)*	2015–2018	1.96 (1.70 to 2.21)*	2018–2021	0.44 (0.31 to 0.58)*	1990–2021	1.97 (1.93 to 2.01)*
Belgium	1990–1995	–0.02 (–0.07 to 0.04)	1995–2000	–0.34 (–0.42 to –0.25)*	2000–2005	–2.83 (–2.90 to –2.75)*	2005–2009	0.70 (0.60 to 0.81)*	2009–2019	–0.18 (–0.20 to –0.16)*	2019–2021	1.27 (1.04 to 1.50)*	1990–2021	–0.40 (–0.43 to –0.38)*
Canada	1990–1994	–1.21 (–1.25 to –1.18)*	1994–2001	–0.65 (–0.67 to –0.63)*	2001–2009	–0.9 (–0.92 to –0.89)*	2009–2015	–0.04 (–0.08 to –0.01)*	2015–2019	1.98 (1.90 to 2.05)*	2019–2021	–4.62 (–4.77 to –4.48)*	1990–2021	–0.60 (–0.61 to –0.58)*
Denmark	1990–1993	5.98 (5.24 to 6.72)*	1993–1996	1.09 (0.19 to 2.01)*	1996–2003	–0.46 (–0.62 to –0.30)*	2003–2009	–1.15 (–1.40 to –0.89)*	2009–2019	–0.86 (–0.97 to –0.75)*	2019–2021	–0.31 (–1.68 to 1.08)	1990–2021	0.04 (–0.10 to 0.19)
Finland	1990–1992	1.57 (1.01 to 2.13)*	1992–1995	0.24 (–0.28 to 0.77)	1995–2000	–3.13 (–3.28 to –2.98)*	2000–2011	–0.44 (–0.48 to –0.40)*	2011–2019	–1.63 (–1.70 to –1.57)*	2019–2021	0.25 (–0.22 to 0.72)	1990–2021	–0.95 (–1.02 to –0.88)*
France	1990–1996	–0.11 (–0.17 to –0.06)*	1996–2000	–0.75 (–0.90 to –0.59)*	2000–2007	–0.99 (–1.04 to –0.94)*	2007–2010	–0.69 (–1.00 to –0.38)*	2010–2017	–0.06 (–0.11 to 0.00)*	2017–2021	0.18 (0.08 to 0.28)*	1990–2021	–0.4 (–0.44 to –0.36)*
Germany	1990–1995	0.16 (–0.07 to 0.39)	1995–1999	5.20 (4.89 to 5.52)*	1999–2002	–0.29 (–0.64 to 0.07)	2002–2009	–1.72 (–1.78 to –1.66)*	2009–2014	–0.21 (–0.32 to –0.09)*	2014–2021	0.27 (0.22 to 0.33)*	1990–2021	0.29 (0.23 to 0.35)*
Greece	1990–1994	0.78 (0.67 to 0.90)*	1994–2000	–0.27 (–0.33 to –0.21)*	2000–2005	2.46 (2.36 to 2.55)*	2005–2011	–0.97 (–1.04 to –0.89)*	2011–2014	–2.74 (–3.15 to –2.32)*	2014–2021	–0.03 (–0.10 to 0.04)	1990–2021	–0.02 (–0.07 to 0.03)
Ireland	1990–1995	0.27 (0.14 to 0.40)*	1995–2003	–1.13 (–1.17 to –1.08)*	2003–2010	–1.28 (–1.33 to –1.23)*	2010–2014	–0.84 (–0.98 to –0.70)*	2014–2019	–0.02 (–0.11 to 0.07)	2019–2021	1.99 (1.52 to 2.47)*	1990–2021	–0.52 (–0.56 to –0.48)*
Italy	1990–1995	–0.54 (–0.80 to –0.28)*	1995–2000	–3.33 (–3.68 to –2.98)*	2000–2005	–1.37 (–1.72 to –1.01)*	2005–2010	1.50 (1.12 to 1.87)*	2010–2019	–0.63 (–0.76 to –0.50)*	2019–2021	10.25 (8.96 to 11.55)*	1990–2021	–0.17 (–0.30 to –0.04)
Luxembourg	1990–1992	0.02 (–0.24 to 0.28)	1992–1995	0.4 (0.19 to 0.61)*	1995–1999	0.92 (0.83 to 1.00)*	1999–2002	–0.01 (–0.17 to 0.15)	2002–2010	–0.25 (–0.27 to –0.23)*	2010–2021	0.07 (0.06 to 0.09)*	1990–2021	0.12 (0.09 to 0.15)*
Netherlands	1990–1993	1.20 (0.94 to 1.47)*	1993–1998	0.72 (0.56 to 0.87)*	1998–2001	–0.12 (–0.62 to 0.38)	2001–2005	–2.73 (–3.01 to –2.45)*	2005–2012	–0.82 (–0.94 to –0.71)*	2012–2021	0.03 (–0.04 to 0.09)	1990–2021	–0.32 (–0.39 to –0.25)*
Norway	1990–1995	–0.12 (–0.23 to 0.00)*	1995–2000	–0.70 (–0.85 to –0.54)*	2000–2005	0.55 (0.39 to 0.71)*	2005–2010	–0.78 (–0.94 to –0.62)*	2010–2014	–0.33 (–0.58 to –0.08)*	2014–2021	0.01 (–0.06 to 0.08)	1990–2021	–0.21 (–0.26 to –0.15)*
Portugal	1990–1995	–0.54 (–0.66 to –0.42)*	1995–2000	–2.69 (–2.82 to –2.56)*	2000–2004	0.92 (0.77 to 1.08)*	2004–2007	0.12 (–0.18 to 0.42)	2007–2015	–0.14 (–0.18 to –0.10)*	2015–2021	0.43 (0.37 to 0.48)*	1990–2021	–0.35 (–0.39 to –0.31)*
Spain	1990–1992	–4.58 (–7.60 to –1.46)*	1992–1995	–1.89 (–3.72 to –0.03)*	1995–2004	1.74 (1.56 to 1.92)*	2004–2010	0.58 (0.30 to 0.85)*	2010–2018	–0.85 (–1.05 to –0.64)*	2018–2021	1.70 (0.92 to 2.49)*	1990–2021	0.07 (–0.21 to 0.34)
Sweden	1990–1994	2.55 (2.37 to 2.74)*	1994–2000	0.85 (0.74 to 0.97)*	2000–2006	1.14 (1.03 to 1.24)*	2006–2010	1.38 (1.15 to 1.61)*	2010–2019	–0.03 (–0.08 to 0.02)	2019–2021	7.09 (6.39 to 7.80)*	1990–2021	1.33 (1.27 to 1.39)*
UK	1990–1998	0.19 (0.14 to 0.24)*	1998–2001	–0.15 (–0.54 to 0.25)	2001–2010	–1.16 (–1.20 to –1.12)*	2010–2013	0.71 (0.34 to 1.08)*	2013–2019	0.95 (0.86 to 1.04)*	2019–2021	0.03 (–0.40 to 0.46)	1990–2021	–0.05 (–0.11 to 0.01)
USA	1990–1994	–2.98 (–3.15 to –2.81)*	1994–2001	0.26 (0.17 to 0.35)*	2001–2004	2.93 (2.62 to 3.24)*	2004–2008	0.70 (0.62 to 0.78)*	2008–2019	0.19 (0.18 to 0.20)*	2019–2021	2.64 (2.47 to 2.80)*	1990–2021	0.27 (0.23 to 0.32)*

*Significantly different from 0 (*p* < 0.001). CI, confidence interval; APC, annual percentage change; AAPC, average annual percentage change.

**Table 2.** **Joinpoint analysis for AF/AFL disease age-standardized incidence rates in EU15+ countries for years 1990–2021 in females**.

Country	Trend1		Trend2		Trend3		Trend4		Trend5		Trend6		AAPC	
	Year	APC (95% CI)	Year	APC (95% CI)	Year	APC (95% CI)	Year	APC (95% CI)	Year	APC (95% CI)	Year	APC (95% CI)	Year	AAPC (95% CI)
Australia	1990–1995	0.54 (0.46 to 0.61)*	1995–2000	0.22 (0.12 to 0.31)*	2000–2005	–0.68 (–0.77 to –0.59)*	2005–2010	0.96 (0.88 to 1.05)*	2010–2018	0.38 (0.34 to 0.42)*	2018–2021	1.06 (0.71 to 1.42)*	1990–2021	0.37 (0.33 to 0.41)*
Austria	1990–2000	1.57 (1.53 to 1.6)*	2000–2004	4.25 (4.08 to 4.42)*	2004–2007	2.30 (2.03 to 2.56)*	2007–2015	1.37 (1.34 to 1.40)*	2015–2018	2.08 (1.87 to 2.28)*	2018–2021	0.38 (0.27 to 0.48)*	1990–2021	1.86 (1.82 to 1.90)*
Belgium	1990–1998	–0.20 (–0.24 to –0.16)*	1998–2001	–0.80 (–1.16 to –0.43)*	2001–2004	–4.81 (–5.12 to –4.49)*	2004–2007	0.49 (0.79 to –0.19)*	2007–2019	–0.14 (–0.16 to –0.11)*	2019–2021	0.63 (0.31 to 0.95)*	1990–2021	–0.66 (–0.72 to –0.61)*
Canada	1990–1994	–1.29 (–1.68 to –0.9)*	1994–2001	–0.40 (–0.62 to –0.19)*	2001–2005	–1.86 (–2.49 to –1.24)*	2005–2010	–3.52 (–3.95 to –3.1)*	2010–2014	2.11 (1.34 to 2.89)*	2014–2021	–0.51 (–0.72 to –0.31)*	1990–2021	–0.92 (–1.08 to –0.77)*
Denmark	1990–1994	3.19 (2.96 to 3.42)*	1994–2000	–0.49 (–0.61 to –0.36)*	2000–2005	0.54 (0.36 to 0.72)*	2005–2010	–1.33 (–1.53 to –1.13)*	2010–2019	–0.76 (–0.83 to –0.68)*	2019–2021	0.44 (–0.36 to 1.24)	1990–2021	–0.01 (–0.08 to 0.06)
Finland	1990–1993	1.57 (1.38 to 1.76)*	1993–1996	–0.61 (–0.91 to –0.31)*	1996–1999	–4.08 (–4.37 to –3.79)*	1999–2011	–0.69 (–0.71 to –0.67)*	2011–2019	–1.8 (–1.84 to –1.76)*	2019–2021	0.10 (–0.25 to 0.45)	1990–2021	–1.04 (–1.08 to –0.99)*
France	1990–1997	–0.43 (–0.47 to –0.39)*	1997–2001	–0.89 (–1.04 to –0.75)*	2001–2009	–1.42 (–1.46 to –1.39)*	2009–2012	–0.44 (–0.72 to –0.16)*	2012–2016	–0.10 (–0.25 to –0.04)	2016–2021	–0.30 (–0.24 to –0.37)*	1990–2021	–0.59 (–0.63 to –0.55)*
Germany	1990–1995	–1.99 (–2.15 to –1.82)*	1995–2000	0.76 (0.62 to 0.89)*	2000–2003	–0.97 (–1.23 to –0.70)*	2003–2010	–1.09 (–1.13 to –1.04)*	2010–2015	0.83 (0.75 to 0.92)*	2015–2021	0.23 (0.17 to 0.28)*	1990–2021	–0.36 (–0.41 to –0.32)*
Greece	1990–1994	3.06 (2.74 to 3.37)*	1994–2000	–0.81 (–1 to –0.62)*	2000–2005	1.70 (1.43 to 1.97)*	2005–2010	–1.35 (–1.61 to –1.09)*	2010–2015	–4.17 (–4.45 to –3.89)*	2015–2021	0.39 (0.21 to 0.58)*	1990–2021	–0.33 (–0.42 to –0.23)*
Ireland	1990–1993	0.17 (–0.44 to 0.78)	1993–1996	–0.88 (–2.04 to 0.30)	1996–2000	–4.9 (–5.42 to –4.38)*	2000–2011	–1.45 (–1.53 to –1.37)*	2011–2019	–0.27 (–0.40 to –0.13)*	2019–2021	7.99 (6.78 to 9.21)*	1990–2021	–0.81 (–0.96 to –0.65)*
Italy	1990–1995	–0.98 (–1.07 to –0.89)*	1995–2000	–0.34 (–0.46 to –0.21)*	2000–2004	–1.19 (–1.39 to –1.00)*	2004–2014	–0.88 (–0.92 to –0.85)*	2014–2019	–0.23 (–0.36 to –0.10)*	2019–2021	4.11 (3.69 to 4.54)*	1990–2021	–0.43 (–0.48 to –0.38)*
Luxembourg	1990–1994	–1.71 (–1.82 to –1.6)*	1994–1997	–0.62 (–0.86 to –0.37)*	1997–2009	–0.24 (–0.26 to –0.23)*	2009–2015	–0.65 (–0.69 to –0.62)*	2015–2019	0.48 (0.4 to 0.56)*	2019–2021	0.16 (–0.04 to 0.36)	1990–2021	–0.43 (–0.46 to –0.40)*
Netherlands	1990–1993	1.21 (0.92 to 1.49)*	1993–1998	0.45 (0.28 to 0.63)*	1998–2001	–0.33 (–0.9 to –0.24)	2001–2005	–3.09 (–3.46 to –2.71)*	2005–2010	–1.58 (–1.88 to –1.28)*	2010–2021	0.15 (0.08 to 0.21)*	1990–2021	–0.45 (–0.54 to –0.36)*
Norway	1990–1995	–0.57 (–0.63 to –0.52)*	1995–2000	–0.71 (–0.79 to –0.64)*	2000–2005	–0.24 (–0.31 to –0.17)*	2005–2010	–0.39 (–0.5 to –0.27)*	2010–2014	–0.09 (–0.06 to –0.12)*	2014–2021	0.15 (0.08 to 0.21)*	1990–2021	–0.52 (–0.55 to –0.49)*
Portugal	1990–1997	–0.77 (–0.8 to –0.74)*	1997–2000	–0.25 (–0.37 to –0.12)*	2000–2005	0.55 (0.52 to 0.59)*	2005–2010	–0.41 (–0.44 to –0.37)*	2010–2018	–0.07 (–0.08 to –0.05)*	2018–2021	0.10 (0.03 to 0.17)*	1990–2021	–0.18 (–0.20 to –0.17)*
Spain	1990–1995	–1.66 (–1.96 to –1.35)*	1995–2000	2.64 (2.36 to 2.92)*	2000–2005	–1.78 (–2.04 to –1.52)*	2005–2010	–0.13 (–0.36 to 0.1)*	2010–2015	–1.72 (–1.99 to –1.45)*	2015–2021	0.05 (–0.13 to 0.23)	1990–2021	–0.43 (–0.52 to –0.33)*
Sweden	1990–1995	1.78 (1.47 to 2.08)*	1995–2000	–2.23 (–2.56 to –1.90)*	2000–2005	0.90 (0.58 to 1.22)*	2005–2010	2.18 (1.85 to 2.50)*	2010–2019	0.02 (–0.10 to 0.13)	2019–2021	13.54 (11.93 to 15.17)*	1990–2021	1.24 (1.11 to 1.38)*
UK	1990–2000	0.30 (0.26 to 0.35)*	2000–2003	–1.05 (–1.56 to –0.54)*	2003–2010	–1.75 (–1.83 to –1.67)*	2010–2013	0.84 (0.34 to 1.33)*	2013–2019	1.33 (1.21 to 1.45)*	2019–2021	–0.06 (–0.64 to 0.52)	1990–2021	–0.07 (–0.15 to 0.01)
USA	1990–1994	2.87 (2.64 to 3.09)*	1994–2000	0.31 (0.16 to 0.47)*	2000–2005	1.56 (1.44 to 1.68)*	2005–2010	–0.72 (–0.78 to –0.67)*	2010–2019	0.30 (0.29 to 0.32)*	2019–2021	2.36 (2.16 to 2.56)*	1990–2021	0.80 (0.75 to 0.84)*

*Significantly different from 0 (*p* < 0.001).

### 3.5 AF/AFL Mortality Trend

**Fig. 4.**
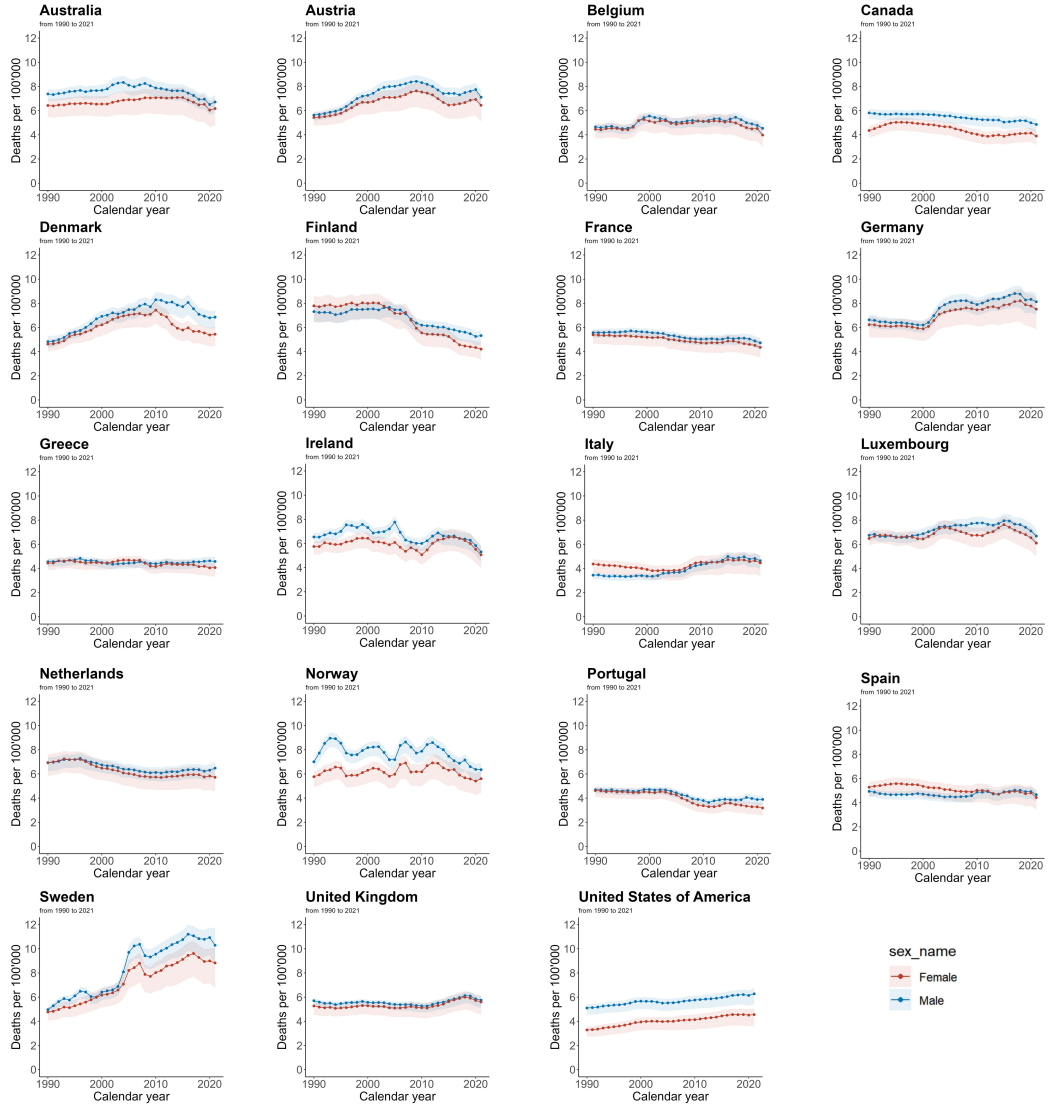
**Trends in age-standardized mortality rates per 100,000 for AF/AFL in EU15+ countries between 1990 and 2021**. Blue squares indicate males and red squares indicate females.

Fig. 4 shows the AF/AFL mortality rate data for each EU15+ country from 1990 to 2021, disaggregated by gender. Among women, a decrease in ASMR was seen in 12 countries, with Finland (–46.0%) and Portugal (–31.1%) showing the largest decreases, while Sweden (+85.0%) and the USA (+38.8%) had the largest increases. In men, during the same period, 11 countries experienced a decline in ASMR, with Finland (–27.2%) and Ireland (–18.9%) showing the largest decreases. However, Sweden (+107.3%) and Denmark (+42.0%) experienced the greatest increases in male ASMR.

### 3.6 Joint Point Analysis of AF/AFL Mortality

Tables 3,4 summarize the joint point analysis results of male and female AF/AFL ASMR from 1990 to 2021. From the AAPC, it is observed that, for most countries, both male and female mortality rates showed a general decreasing trend. Finland showed the largest decrease in female (–1.93%, 95% CI: –2.39%, –1.47%) and male mortality rates (–1.06%, 95% CI: –1.43%, –0.69%). Conversely, Sweden experienced the largest increase in mortality rates for both sexes, with females showing a +1.97% increase (95% CI: 1.49%, 2.46%) and males a +2.38% increase (95% CI: 1.27%, 3.50%). According to the average percentage change, Finland exhibited the most significant decrease in female mortality rates during the period 2007–2010 (–8.05%), while Ireland showed the largest decline in male mortality rates from 2005–2008 (–7.92%). Additionally, Sweden showed the most rapid increase in mortality rates during 2003–2006, with female mortality increasing by 9.87% and male mortality by 13.48%.

**Table 3.** **Joinpoint analysis for AF/AFL disease age-standardized mortality rates in EU15+ countries for years 1990–2021 in males**.

Country	Trend1		Trend2		Trend3		Trend4		Trend5		Trend6		AAPC	
	Year	APC (95% CI)	Year	APC (95% CI)	Year	APC (95% CI)	Year	APC (95% CI)	Year	APC (95% CI)	Year	APC (95% CI)	Year	AAPC (95% CI)
Australia	1990–1995	0.75 (0.09 to 1.41)*	1995–2000	0.15 (–0.87 to 1.17)	2000–2003	2.57 (–1.15 to 6.44)	2003–2008	–0.45 (–1.67 to 0.78)	2008–2015	–0.94 (–1.62 to –0.26)*	2015–2021	–2.37 (–3.12 to –1.61)*	1990–2021	–0.36 (–0.82 to 0.10)
Austria	1990–1995	1.57 (0.71 to 2.44)*	1995–1998	4.81 (0.74 to 9.04)*	1998–2003	2.51 (1.09 to 3.95)*	2003–2010	0.98 (0.18 to 1.79)*	2010–2014	–3.12 (–5.57 to –0.62)*	2014–2021	0.01 (–0.70 to 0.74)	1990–2021	0.92 (0.35 to 1.50)
Belgium	1990–1993	0.21 (–1.51 to 1.96)	1993–1996	–1.06 (–4.60 to 2.60)	1996–2000	5.75 (3.93 to 7.61)*	2000–2004	–2.85 (–4.58 to –1.10)*	2004–2016	0.53 (0.27 to 0.80)*	2016–2021	–3.06 (–4.08 to –2.04)*	1990–2021	–0.03 (–0.53 to 0.47)
Canada	1990–1992	–0.94 (–2.06 to 0.20)	1992–1999	0.03 (–0.17 to 0.22)	1999–2002	–0.35 (–1.54 to 0.85)	2002–2015	–0.79 (–0.87 to –0.71)*	2015–2019	0.30 (–0.46 to 1.06)	2019–2021	–3.09 (–4.71 to –1.45)*	1990–2021	–0.58 (–0.77 to –0.39)*
Denmark	1990–1996	3.35 (2.56 to 4.16)*	1996–2000	4.58 (2.43 to 6.78)*	2000–2011	1.55 (1.16 to 1.93)*	2011–2016	–0.84 (–2.55 to 0.91)	2016–2019	–4.35 (–9.43 to 1.02)	2019–2021	–0.28 (–5.79 to 5.55)	1990–2021	1.18 (0.46 to 1.91)
Finland	1990–1994	–0.86 (–1.75 to 0.04)	1994–1998	1.49 (0.12 to 2.88)*	1998–2006	0.03 (–0.32 to 0.39)	2006–2009	–5.79 (–8.45 to –3.05)*	2009–2014	–0.99 (–1.94 to –0.04)*	2014–2021	–1.83 (–2.26 to –1.39)*	1990–2021	–1.06 (–1.43 to –0.69)*
France	1990–1994	0.11 (–0.30 to 0.52)	1994–1997	0.69 (–0.58 to 1.99)	1997–2002	–0.62 (–1.04 to –0.20)*	2002–2009	–1.41 (–1.65 to –1.16)*	2009–2018	0.24 (0.06 to 0.42)*	2018–2021	–2.48 (–3.42 to –1.54)*	1990–2021	–0.51 (–0.69 to –0.34)*
Germany	1990–2000	–0.70 (–0.87 to –0.53)*	2000–2004	6.56 (5.37 to 7.76)*	2004–2007	1.34 (–1.03 to 3.78)	2007–2010	–1.42 (–3.83 to 1.04)	2010–2017	1.49 (1.06 to 1.93)*	2017–2021	–2.00 (–2.91 to –1.09)*	1990–2021	0.66 (0.29 to 1.03)*
Greece	1990–1993	0.29 (–0.92 to 1.52)	1993–1996	1.4 (–1.00 to 3.85)	1996–2002	–1.64 (–2.19 to –1.09)*	2002–2007	0.8 (–0.02 to 1.62)	2007–2010	–0.86 (–3.54 to 1.90)	2010–2021	0.41 (0.21 to 0.61)*	1990–2021	0.03 (–0.35 to 0.41)
Ireland	1990–1999	1.80 (1.20 to 2.40)*	1999–2002	–3.89 (–10.30 to 2.98)	2002–2005	3.94 (–3.58 to 12.06)	2005–2008	–7.92 (–15.03 to –0.22)*	2008–2015	2.11 (0.59 to 3.65)*	2015–2021	–3.26 (–5.03 to –1.47)*	1990–2021	–0.46 (–1.70 to 0.79)
Italy	1990–1993	–1.13 (–2.90 to 0.67)	1993–2001	0.12 (–0.39 to 0.64)	2001–2006	1.91 (0.62 to 3.22)*	2006–2009	4.17 (–0.16 to 8.69)	2009–2016	2.35 (1.56 to 3.14)*	2016–2021	–0.95 (–2.08 to 0.19)	1990–2021	1.00 (0.47 to 1.53)*
Luxembourg	1990–1997	–0.49 (–0.83 to –0.14)*	1997–2004	1.89 (1.36 to 2.43)*	2004–2010	0.61 (–0.12 to 1.35)	2010–2013	–0.61 (–3.86 to 2.76)	2013–2016	1.72 (–1.69 to 5.25)	2016–2021	–3.09 (–3.97 to –2.21)*	1990–2021	0.03 (–0.45 to 0.51)
Netherlands	1990–1996	0.79 (0.52 to 1.06)*	1996–2000	–1.86 (–2.65 to –1.07)*	2000–2010	–1.10 (–1.26 to –0.95)*	2010–2016	0.84 (0.41 to 1.28)*	2016–2019	–0.49 (–2.36 to 1.41)	2019–2021	1.57 (–0.35 to 3.52)	1990–2021	–0.23 (–0.48 to 0.01)
Norway	1990–1993	9.34 (3.93 to 15.04)*	1993–1997	–4.96 (–9.92 to 0.26)	1997–2001	2.66 (–2.75 to 8.37)	2001–2004	–3.38 (–14.34 to 8.99)	2004–2012	1.51 (–0.25 to 3.30)	2012–2021	–3.46 (–4.80 to –2.10)*	1990–2021	–0.42 (–1.98 to 1.16)
Portugal	1990–1997	–0.42 (–0.80 to –0.03)*	1997–2000	1.02 (–1.87 to 4.00)	2000–2003	–0.14 (–3.13 to 2.94)	2003–2011	–3.03 (–3.50 to –2.57)*	2011–2014	2.09 (–1.99 to 6.33)	2014–2021	0.11 (–0.49 to 0.71)	1990–2021	–0.58 (–1.13 to –0.03)
Spain	1990–1994	–1.51 (–2.60 to –0.42)*	1994–1999	0.38 (–0.74 to 1.51)	1999–2007	–0.84 (–1.35 to –0.32)*	2007–2010	2.70 (–1.51 to 7.09)	2010–2019	0.47 (–0.01 to 0.95)	2019–2021	–2.97 (–7.44 to 1.71)	1990–2021	–0.15 (–0.69 to 0.39)
Sweden	1990–1996	3.67 (2.47 to 4.89)*	1996–2002	0.04 (–1.66 to 1.77)	2002–2006	13.48 (8.87 to 18.27)*	2006–2009	–4.09 (–11.87 to 4.37)	2009–2016	2.64 (1.08 to 4.23)*	2016–2021	–1.03 (–3.51 to 1.50)	1990–2021	2.38 (1.27 to 3.50)*
UK	1990–1994	–1.17 (–1.63 to –0.71)*	1994–1999	0.82 (0.36 to 1.29)*	1999–2005	–0.63 (–1.00 to –0.27)*	2005–2011	–0.51 (–0.94 to –0.07)*	2011–2018	2.19 (1.82 to 2.57)*	2018–2021	–1.99 (–3.09 to –0.89)*	1990–2021	0.05 (–0.13 to 0.24)
USA	1990–1997	0.89 (0.72 to 1.07)*	1997–2000	1.65 (0.30 to 3.02)*	2000–2005	–0.68 (–1.11 to –0.25)*	2005–2015	0.82 (0.69 to 0.95)*	2015–2018	1.14 (–0.41 to 2.70)*	2018–2021	0.22 (–0.59 to 1.04)*	1990–2021	0.65 (0.43 to 0.86)*

*Significantly different from 0 (*p* < 0.001).

**Table 4.** **Joinpoint analysis for AF/AFL disease age-standardized mortality rates in EU15+ countries for years 1990–2021 in females**.

Country	Trend1		Trend2		Trend3		Trend4		Trend5		Trend6		AAPC	
	Year	APC (95% CI)	Year	APC (95% CI)	Year	APC (95% CI)	Year	APC (95% CI)	Year	APC (95% CI)	Year	APC (95% CI)	Year	AAPC (95% CI)
Australia	1990–1996	0.53 (0.12 to 0.94)*	1996–2001	–0.15 (–0.97 to 0.66)	2001–2004	1.58 (–1.21 to 4.45)	2004–2009	0.54 (–0.38 to 1.48)	2009–2015	0.02 (–0.69 to 0.73)	2015–2021	–2.49 (–3.06 to –1.91)*	1990–2021	–0.17 (–0.52 to 0.19)*
Austria	1990–1995	1.24 (0.73 to 1.75)*	1995–1998	4.39 (1.93 to 6.91)*	1998–2010	1.35 (1.17 to 1.54)*	2010–2015	–3.40 (–4.3 to –2.49)*	2015–2019	1.46 (–0.12 to 3.07)	2019–2021	–2.07 (–5.37 to 1.34)	1990–2021	0.63 (0.24 to 1.02)*
Belgium	1990–1996	–0.07 (–0.93 to 0.79)	1996–1999	5.82 (0.36 to 11.58)*	1999–2005	–1.26 (–2.45 to –0.06)*	2005–2010	1.01 (–0.83 to 2.89)	2010–2016	–0.43 (–1.81 to 0.98)	2016–2021	–3.78 (–5.24 to –2.30)*	1990–2021	–0.25 (–0.94 to 0.44)*
Canada	1990–1995	3.12 (2.89 to 3.36)*	1995–2005	–0.94 (–1.04 to –0.85)*	2005–2011	–2.84 (–3.10 to –2.58)*	2011–2015	0.12 (–0.52 to 0.76)	2015–2019	1.50 (0.84 to 2.16)*	2019–2021	–2.71 (–4.05 to –1.35)*	1990–2021	–0.34 (–0.49 to –0.19)*
Denmark	1990–1992	1.43 (–2.19 to 5.18)*	1992–1995	4.19 (0.69 to 7.82)*	1995–2004	3.01 (2.58 to 3.45)*	2004–2011	0.45 (–0.26 to 1.16)	2011–2014	–6.29 (–10.29 to –2.12)*	2014–2021	–1.37 (–2.01 to –0.73)*	1990–2021	0.51 (–0.07 to 1.10)*
Finland	1990–2001	0.37 (0.18 to 0.56)*	2001–2007	–2.11 (–2.74 to –1.48)*	2007–2010	–8.05 (–10.80 to –5.22)*	2010–2014	–0.35 (–1.91 to 1.25)	2014–2017	–5.39 (–8.35 to –2.33)*	2017–2021	–2.11 (–3.12 to –1.09)*	1990–2021	–1.93 (–2.39 to –1.47)*
France	1990–1996	–0.27 (–0.49 to –0.06)*	1996–2003	–0.45 (–0.67 to –0.22)*	2003–2006	–1.50 (–2.94 to –0.05)*	2006–2011	–0.88 (–1.36 to –0.39)*	2011–2016	0.77 (0.23 to 1.31)*	2016–2021	–2.07 (–2.46 to –1.68)*	1990–2021	–0.65 (–0.84 to –0.47)*
Germany	1990–1997	–0.25 (–0.58 to 0.08)	1997–2000	–1.3 (–3.73 to 1.19)	2000–2004	6.01 (4.67 to 7.38)*	2004–2014	0.39 (0.15 to 0.64)*	2014–2018	1.67 (0.20 to 3.17)*	2018–2021	–2.91 (–4.49 to –1.30)*	1990–2021	0.63 (0.26 to 0.99)*
Greece	1990–1993	1.55 (0.09 to 3.04)*	1993–2000	–0.63 (–1.13 to –0.13)*	2000–2006	1.16 (0.51 to 1.81)*	2006–2010	–3.36 (–4.92 to –1.79)*	2010–2013	2.07 (–1.54 to 5.81)	2013–2021	–1.03 (–1.45 to –0.61)*	1990–2021	–0.28 (–0.72 to 0.15)*
Ireland	1990–1999	1.21 (0.75 to 1.68)*	1999–2005	–1.33 (–2.60 to –0.05)*	2005–2010	–2.62 (–4.65 to –0.54)*	2010–2013	7.05 (–0.29 to 14.92)	2013–2017	0.91 (–2.72 to 4.67)	2017–2021	–5.80 (–8.42 to –3.10)*	1990–2021	–0.33 (–1.25 to 0.60)*
Italy	1990–1998	–0.89 (–1.16 to –0.62)*	1998–2001	–2.00 (–4.62 to 0.70)	2001–2006	0.09 (–0.82 to 1.02)	2006–2009	4.99 (1.79 to 8.28)*	2009–2017	0.74 (0.31 to 1.18)*	2017–2021	–1.08 (–2.16 to 0.01)	1990–2021	0.11 (–0.32 to 0.54)*
Luxembourg	1990–1992	2.03 (–0.32 to 4.42)	1992–2000	–0.58 (–0.93 to –0.22)*	2000–2004	3.50 (1.97 to 5.07)*	2004–2011	–1.6 (–2.13 to –1.06)*	2011–2015	3.67 (1.98 to 5.37)*	2015–2021	–3.28 (–3.92 to –2.64)*	1990–2021	–0.12 (–0.47 to 0.23)*
Netherlands	1990–1993	1.4 (0.51 to 2.29)*	1993–1996	–0.04 (–1.74 to 1.68)	1996–1999	–2.81 (–4.52 to –1.08)*	1999–2009	–1.49 (–1.66 to –1.33)*	2009–2017	0.54 (0.26 to 0.82)*	2017–2021	–0.90 (–1.56 to –0.23)*	1990–2021	–0.61 (–0.86 to –0.35)*
Norway	1990–1994	3.47 (0.16 to 6.88)*	1994–1997	–4.41 (–14.30 to 6.61)	1997–2001	2.92 (–2.61 to 8.77)	2001–2004	–2.1 (–12.43 to 9.45)	2004–2013	1.21 (–0.19 to 2.64)	2013–2021	–2.93 (–4.43 to –1.40)*	1990–2021	–0.25 (–1.90 to 1.43)*
Portugal	1990–1994	–0.60 (–1.15 to –0.05)*	1994–2004	–0.12 (–0.29 to 0.06)	2004–2009	–5.21 (–5.89 to –4.52)*	2009–2012	–1.27 (–3.69 to 1.20)	2012–2015	2.94 (0.29 to 5.67)*	2015–2021	–2.01 (–2.49 to –1.54)*	1990–2021	–1.21 (–1.56 to –0.85)*
Spain	1990–1996	0.94 (0.52 to 1.37)*	1996–2008	–1.16 (–1.35 to –0.97)*	2008–2011	1.01 (–2.17 to 4.30)	2011–2014	–2.15 (–5.41 to 1.22)	2014–2017	1.93 (–1.55 to 5.54)	2017–2021	–2.34 (–3.52 to –1.14)*	1990–2021	–0.50 (–1.05 to 0.04)*
Sweden	1990–1995	2.20 (1.56 to 2.84)*	1995–2003	2.74 (2.33 to 3.16)*	2003–2006	9.87 (6.35 to 13.50)*	2006–2009	–3.75 (–6.90 to –0.50)*	2009–2017	2.64 (2.14 to 3.14)*	2017–2021	–2.36 (–3.60 to –1.10)*	1990–2021	1.97 (1.49 to 2.46)*
UK	1990–1994	–0.85 (–1.40 to –0.30)*	1994–2000	0.78 (0.39 to 1.18)*	2000–2004	–0.80 (–1.73 to 0.13)	2004–2011	–0.07 (–0.43 to 0.28)	2011–2018	2.34 (1.95 to 2.72)*	2018–2021	–2.3 (–3.47 to –1.11)*	1990–2021	0.22 (0.00 to 0.43)*
USA	1990–1996	1.66 (1.46 to 1.85)*	1996–2000	2.46 (1.87 to 3.06)*	2000–2006	0.16 (–0.10 to 0.43)	2006–2011	0.86 (0.45 to 1.27)*	2011–2017	1.42 (1.13 to 1.72)*	2017–2021	–0.11 (–0.54 to 0.31)	1990–2021	1.07 (0.93 to 1.20)*

*Significantly different from 0 (*p* < 0.001).

### 3.7 AF/AFL MII Trend

**Supplementary Fig. 1 (Supplementary Material 1)** presents the MII (Mortality-to-Incidence Ratio) data for both men and women in EU15+ countries in 1990 and 2021. In 1990, the median MII for women was 0.095, and for men, it was 0.067. The highest MII values for women were observed in the UK (0.127), Austria (0.118), and Australia (0.117), while Canada (0.054), the USA (0.058), and Italy (0.060) had the lowest values. For men in 1990, the highest MII values were in the Netherlands (0.089), the UK (0.088), and Luxembourg (0.087), while Italy (0.045), Canada (0.046), and the USA (0.051) had the lowest values. In 2021, the median MII for women was 0.092, and for men, it was 0.072. The highest MII values for women in 2021 were seen in the UK (0.138), Germany (0.135), and Luxembourg (0.122), while Portugal (0.057), the USA (0.062), and Canada (0.065) had the lowest values. For men in 2021, the highest MII values were in the Netherlands (0.092), the UK (0.090), and Luxembourg (0.083), while Canada (0.047), Austria (0.054), and Spain (0.056) had the lowest values. In 1990, the MII for women was higher than for men in all EU15+ countries. In 2021, except for Portugal, where the male MII was higher, the MII for women remained higher than for men.

The MII trends for each country, categorized by gender, are shown in **Supplementary Fig. 2 (Supplementary Material 1)**. It is observed that the trend changes for MII in both men and women were not uniform across countries. Among women, 12 countries experienced an upward trend in MII, with the largest increase in Germany (+35.62%). Conversely, 7 countries showed a downward trend, with the largest decrease in Austria (–32.58%). Among men, 10 countries saw an increase in MII, with the largest increase in Sweden (+39.89%), while the remaining 9 countries showed a decrease, with Austria again showing the largest decrease (–30.66%).

**Supplementary Tables 1,2 (Supplementary Material 1)** present the joint point analysis results for MII data of men and women in EU15+ countries during the observation period. The trend changes for each country were not uniform across the different trend intervals, and the average percentage change values indicate the following key observations: From 1996 to 1999, the MII for women in Ireland increased the fastest (+8.83%), while from 2019 to 2021, it decreased the slowest (–16.07%). Among men, from 2003 to 2006, Sweden saw the fastest increase in MII (+15.00%), and from 2019 to 2021, Italy experienced the largest decrease in MII (–11.15%). These inflection points were all statistically significant. According to the AAPC values, most EU15+ countries exhibited an overall upward trend in MII for both men and women. The fastest increase in MII was observed in Germany for women (+1.00%, 95% CI: 0.58%, 1.41%) and Italy for men (+1.15%, 95% CI: 0.60%, 1.71%). The largest decrease in MII was seen in Austria, with a –1.06% decrease (95% CI: –1.66%, –0.45%) in men and –1.15% (95% CI: –1.61%, –0.70%) in women.

### 3.8 Net Drift and Local Drifts Analysis

Net Drift represents the overall annual percentage change in incidence rates from 1992 to 2021. Local Drifts reflect the annual percentage change in incidence rates for specific age groups relative to the net drift. Table 5 and **Supplementary Material 3** summarizes the Net Drift and Local Drifts for each age group across the EU15+ countries.During the observation period, Luxembourg exhibited a constant Net Drift with no gender differences. Specifically, the Net Drift for females was –0.46% (95% CI: –1.62%, 0.70%, *p* = 0.43), for males it was 0.00% (95% CI: –1.24%, 1.26%, *p* = 1.00), and for both genders combined, it was –0.19% (95% CI: –0.85%, 0.47%, *p* = 0.57). Additionally, Denmark showed a stable Net Drift for women at –0.19% (95% CI: –0.47%, 0.09%, *p* = 0.18), while Australia showed a stable Net Drift for both genders combined at –0.06% (95% CI: –0.14%, 0.02%, *p* = 0.12). Aside from these three countries, all other countries had statistically significant Net Drift (*p* < 0.05). Among them, 13 countries showed a decreasing trend with no gender differences, while 3 countries (Austria, Sweden, and the USA) showed an increasing trend. Regarding gender-specific annual percentage changes, the range of Net Drift was as follows: For females: Ireland showed the most significant decrease at –1.32% (95% CI: –1.73%, –0.90%), while Austria showed the highest increase at 1.63% (95% CI: 1.37%, 1.89%). For males: Finland showed the most significant decrease at –1.17% (95% CI: –1.47%, –0.86%), while Austria showed the highest increase at 1.85% (95% CI: 1.60%, 2.09%). For both genders: Finland showed the most significant decrease at –1.28% (95% CI: –1.44%, –1.12%), while Austria showed the highest increase at 1.82% (95% CI: 1.69%, 1.96%). Local Drifts also reflect the differences in incidence trends for specific age groups. The trend changes for each country were not uniform, with most countries showing local drift values mainly below 0, indicating improvements in AF/AFL incidence. Specifically, excluding countries where time trends were the same for each age group (Finland, Ireland, Italy, Luxembourg, and Norway, with *p* > 0.05), 12 countries showed a single downward trend for the elderly group (aged 75 and above). The most significant decrease in this group was observed in UK males at –3.55% (95% CI: –5.08%, –2.00%). For the younger age groups, countries like Australia, Austria, Belgium, Canada, France, Sweden, and the USA exhibited single upward trends in local drift. Among these, the most significant increase was seen in Belgian males, with an increase of 1.86% (95% CI: 1.19%, 2.54%).

**Table 5.** **The net drifts for EU15+ in female and male both (%)**.

Country	NetDrift_Female	NetDrift_Male	NetDrift_Both
Australia	0.16 (0.03, 0.29)	–0.16 (–0.30, –0.02)	–0.06 (–0.14, 0.02)
Austria	1.63 (1.37, 1.89)	1.85 (1.60, 2.09)	1.82 (1.69, 1.96)
Belgium	–0.38 (–0.59, –0.16)	–0.49 (–0.77, –0.20)	–0.51 (–0.69, –0.32)
Canada	–0.87 (–1.02, –0.72)	–0.26 (–0.38, –0.13)	–0.44 (–0.56, –0.32)
Denmark	–0.19 (–0.47, 0.09)	–0.73 (–0.99, –0.47)	–0.58 (–0.73, –0.43)
Finland	–1.30 (–1.63, –0.98)	–1.17 (–1.47, –0.86)	–1.28 (–1.44, –1.12)
France	–0.81 (–0.91, –0.71)	–0.62 (–0.71, –0.52)	–0.72 (–0.77, –0.67)
Germany	–0.46 (–0.81, –0.12)	–0.26 (–0.42, –0.10)	–0.42 (–0.61, –0.22)
Greece	–1.18 (–1.42, –0.93)	–0.33 (–0.54, –0.13)	–0.64 (–0.78, –0.50)
Ireland	–1.32 (–1.73, –0.90)	–0.94 (–1.38, –0.49)	–1.11 (–1.35, –0.87)
Italy	–0.63 (–0.88, –0.39)	–0.63 (–0.87, –0.40)	–0.67 (–0.84, –0.49)
Luxembourg	–0.46 (–1.62, 0.70)	–0.00 (–1.24, 1.26)	–0.19 (–0.85, 0.47)
Netherlands	–0.99 (–1.18, –0.80)	–0.75 (–0.92, –0.58)	–0.91 (–1.01, –0.81)
Norway	–0.61 (–0.96, –0.25)	–0.36 (–0.61, –0.11)	–0.43 (–0.60, –0.25)
Portugal	–0.56 (–0.81, –0.31)	–0.56 (–0.85, –0.28)	–0.58 (–0.74, –0.42)
Spain	–0.67 (–0.84, –0.50)	0.25 (0.11, 0.38)	–0.18 (–0.29, –0.07)
Sweden	0.60 (0.41, 0.79)	0.67 (0.51, 0.82)	0.66 (0.56, 0.77)
UK	–0.35 (–0.46, –0.25)	–0.46 (–0.56, –0.36)	–0.39 (–0.45, –0.33)
USA	0.24 (0.14, 0.34)	0.40 (0.24, 0.55)	0.27 (0.16, 0.38)

Net drifts: the overall annual percentage change in the age-standardized rate based on period and birth cohort. All of net drifts were statistically significant (*p* < 0.05). Luxembourg, Denmark females and Australia both was excluded because these data didn’t meet the age–period–cohort (APC) model’s requirements.

### 3.9 APC Analysis of AF/AFL Incidence Trends

**Fig. 5.**
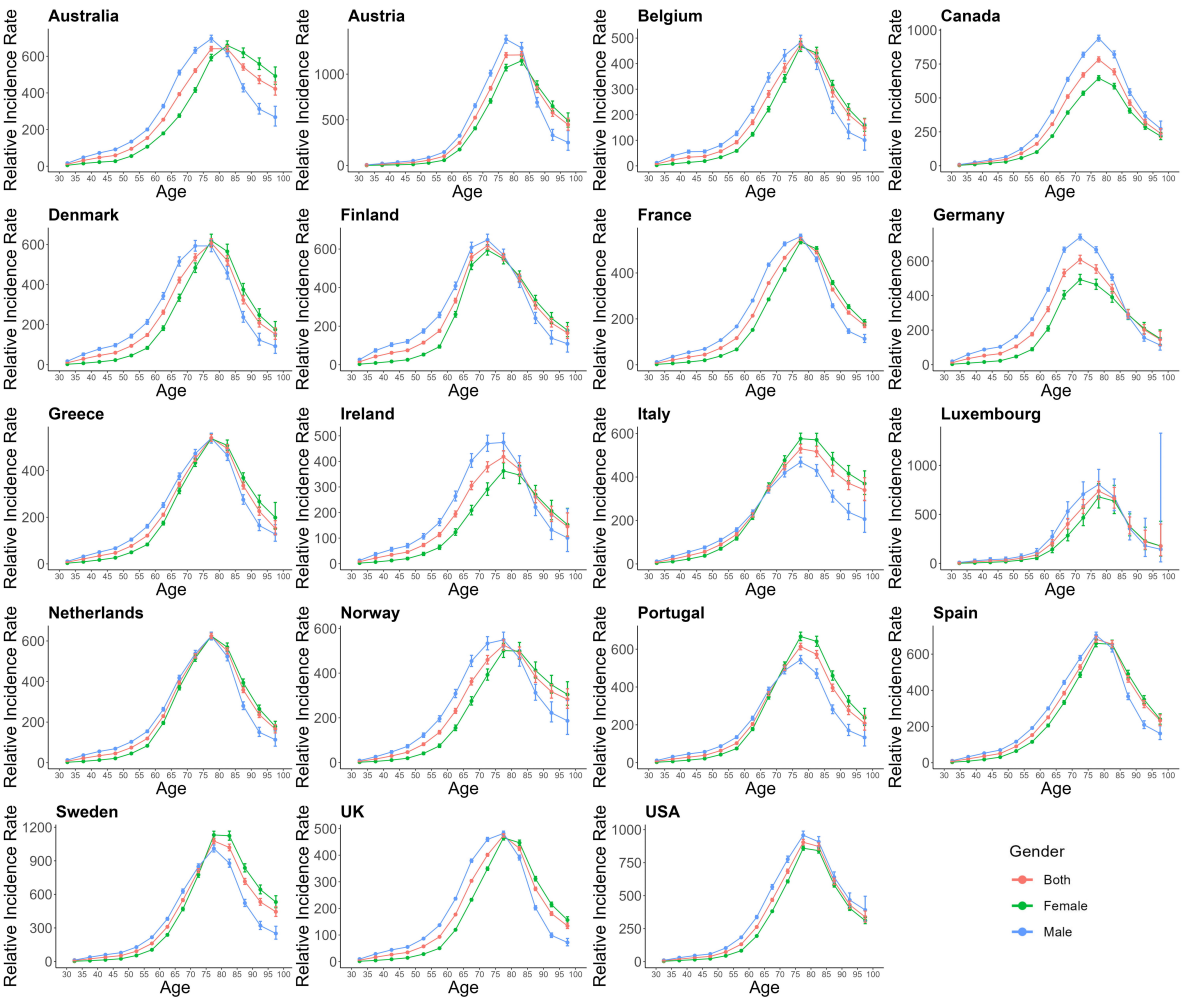
**The age effects on AF/AFL for EU15+ in female and male both**. Red lines indicate both, green lines indicate females, and Blue lines indicate males.

**Fig. 6.**
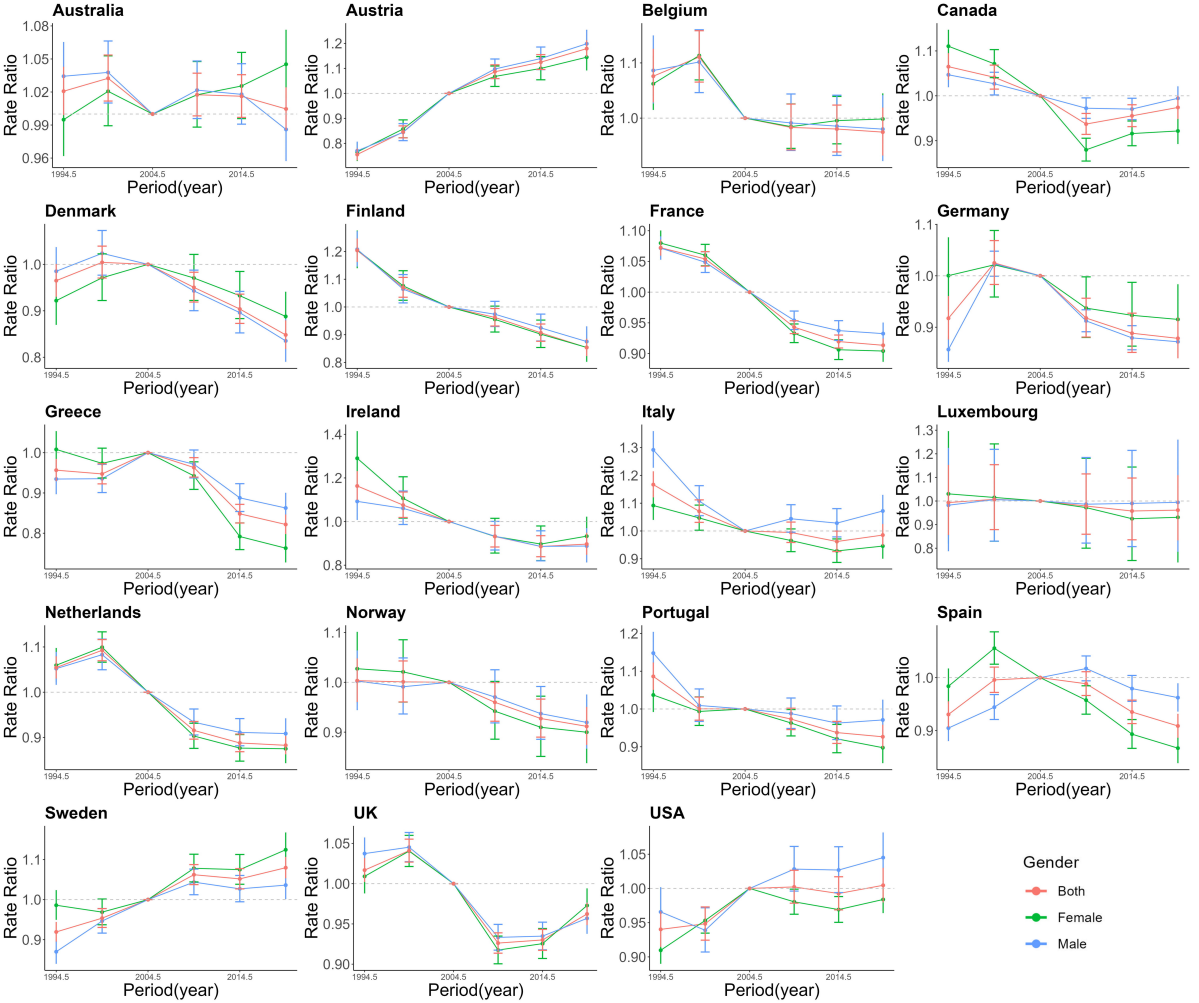
**The period effects on AF/AFL for EU15+ in female and male both**. Red lines indicate both, green lines indicate females, and Blue lines indicate males.

**Fig. 7.**
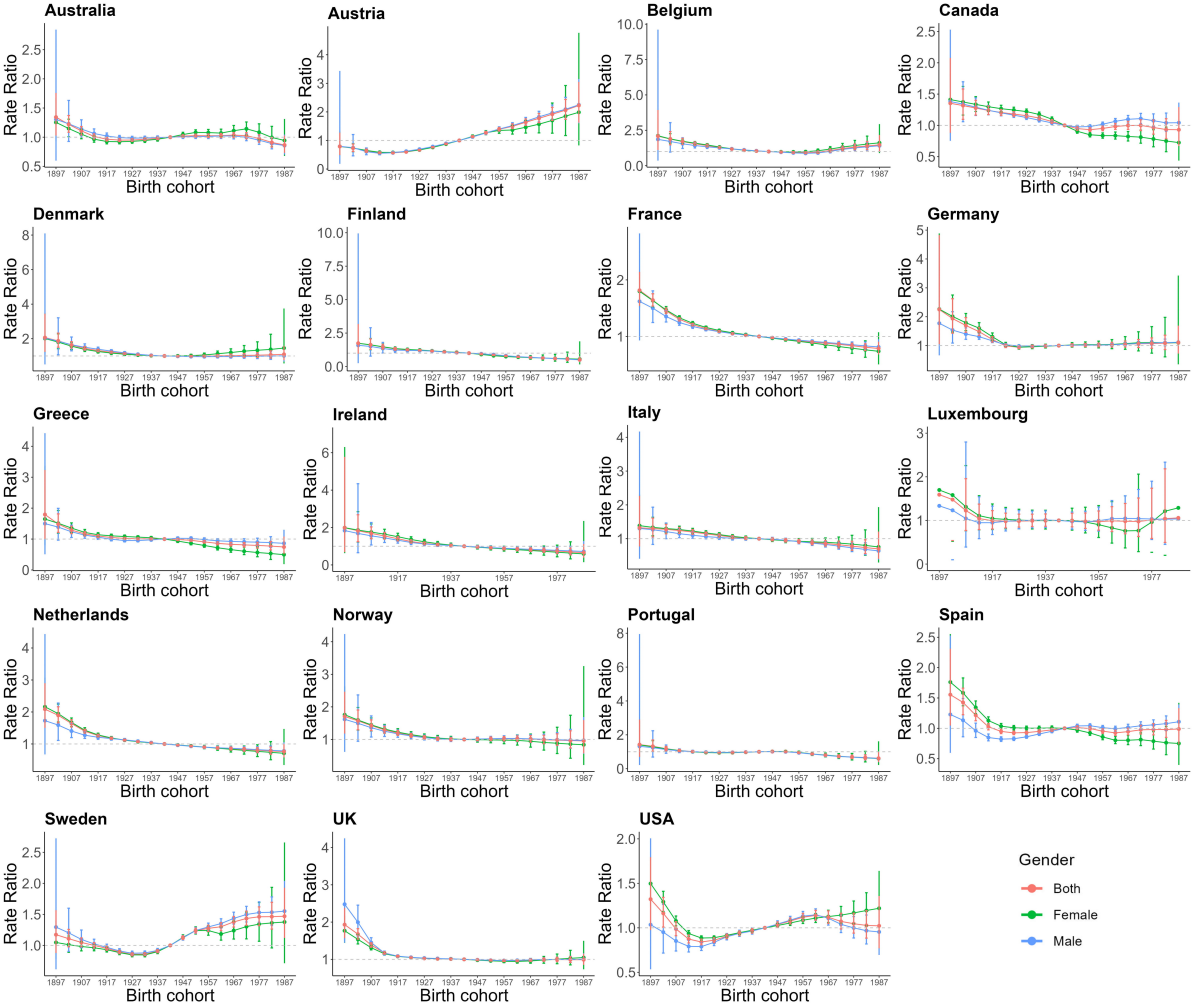
**The cohort effects on AF/AFL for EU15+ in female and male both**. Red lines indicate both, green lines indicate females, and Blue lines indicate males.

Figs. 5,6,7 provides the estimates of the effects of age, period, and cohort on the incidence of AF/AFL. According to the Wald chi-squared test results, 10 countries show statistically significant results. These countries (Austria, Belgium, Canada, Denmark, France, Greece, Spain, Sweden, UK, and USA) will be analyzed further.

From Fig. 5, we can observe the age-specific longitudinal curves of AF/AFL incidence by gender. In all countries, the incidence of AF/AFL generally increases with age. However, the highest risk of AF/AFL occurs in the age groups of 70–74 years and 75–79 years in all countries, followed by a decrease in risk with further age. A comparison of AF/AFL incidence rates across EU15+ countries for each age group shows notable age differences. Except for Canada and USA, where the male incidence rates are higher than those of females across all age groups, in the other 8 countries, the incidence of AF/AFL in the elderly population (aged 75 and above) shows a shift in gender differences. Specifically, in Denmark, Greece, Sweden, and UK, the shift occurs in the 75–79 years age group, while in France, Spain, and Belgium, the shift occurs in those aged below 80 years, and in Austria, the shift is observed in the 80–85 years age group. After these age groups, female incidence rates exceed male incidence rates, marking a reversal from previously being lower.

From Fig. 6, The trends in the period effects of AF/AFL incidence across all countries from 1990 to 2021 are not uniform. Seven countries show a general decline in period effects, whereas Austria, Sweden, and USA show an increasing trend. Specifically, France exhibits the most significant overall decline, while Austria shows the most noticeable increase. In more recent years, gender differences in the relative risk of AF/AFL incidence appear. In six countries, the relative risk for women is lower than that for men, while in Denmark and Sweden, women have a higher relative risk than men. In Belgium and UK, gender differences exhibit a fluctuating trend, with women in these countries showing an increasing risk of AF/AFL in recent years.

From Fig. 7, the cohort effects on AF/AFL incidence show that in seven countries, individuals born earlier (e.g., in the 1900s) have a higher incidence compared to those born later (e.g., in the 1980s). However, the overall cohort effect shows a decreasing trend in incidence. Conversely, Austria and Sweden exhibit an opposite trend, with incidence rates increasing in more recent birth cohorts. We also observe that the trends in cohort-related incidence rates are generally consistent for both males and females. The only exception is USA, where the cohort incidence for women born after 1967 shows a significant upward trend in contrast to males.

## 4. Discussion

Most studies on trends in the burden of disease for AF/AFL have primarily focused on global comparisons, while few in-depth, localized analyses have been conducted in high-income countries. The aim of this study was to analyze trends in the disease burden of AF/AFL in high-income countries and explore differences in trends to inform public health policy. The study analyzed gender-specific trends in ASIR, ASMR, and MII for AF/AFL in EU15+ countries from 1990 to 2021, revealing several key findings: (i) 11 out of 19 EU15+ countries showed that ASIR and ASMR for both males and females exhibited decreasing trends; (ii) ASIR, ASMR, and MII in certain countries exhibited varying trends, indicating variations in treatment outcomes and disease burden across countries; (iii) Gender differences in AF/AFL were observed across all countries studied, with males exhibiting higher ASIR and ASMR than females overall, but age-specific gender differences were also present, with older women generally exhibiting higher ASIR and ASMR than men.

This study reveals divergent trends in ASIR and ASMR of atrial fibrillation/heart failure across EU15+ countries from 1990 to 2021, with most nations demonstrating declining patterns. The observed ASIR reduction may primarily stem from enhanced primary prevention and diagnostic practices. Earlier detection through expanded screening programs and heightened disease awareness in high-income settings may also decrease the interval between AF onset and clinical diagnosis, thereby attenuating “new case” accumulation in later stages. Conversely, ASMR declines likely reflect advancements in secondary prevention and therapeutic interventions. Improved long-term prognosis for diagnosed patients has been achieved through increased adoption of rhythm control strategies, including radiofrequency ablation and novel antiarrhythmic agents (e.g., dronedarone, vernakalant) [5]. Optimized anticoagulation protocols [7, 21], particularly the widespread implementation of non-vitamin K antagonist oral anticoagulants (NOACs) guided by the 2020 ESC guidelines, have demonstrated superior efficacy in reducing thromboembolic complications compared to vitamin K antagonists (VKAs) [22, 23, 24, 25]. Dronedarone, incorporated into clinical practice since the 2010 ESC guideline update [21], enables pharmacological cardioversion of recent-onset atrial fibrillation within 24 hours. Its unique mechanism involving blockade of early-activated K+ channels and frequency-dependent Na+ channels minimizes ventricular repolarization effects, substantially reducing Torsades de Pointes (TdP) risk [26, 27]. Multicenter trials [28] demonstrate that dronedarone treatment significantly prolongs time to atrial fibrillation recurrence in paroxysmal or persistent cases. Therapeutic adoption patterns show strong correlation with national healthcare reimbursement policies. Countries including Norway [29], Australia [30], Canada [31], and Finland [32] have observed increased NOACs utilization following relaxation of reimbursement restrictions.

While pulmonary vein isolation cannot prevent initial atrial fibrillation onset, its expanded application since the 2010s [21] has contributed to ASMR reduction through decreased arrhythmia recurrence and stroke risk. Declining MII in nations with expanded ablation access corroborate this trend, suggesting improved rhythm control mitigates disease progression despite rising diagnostic rates.

European initiatives promoting anticoagulation therapy and patient education have yielded significant impacts. Countries like Belgium, Italy, the Netherlands, and Spain have established formal anticoagulation clinics implementing systematic management strategies. These programs facilitate timely anticoagulation initiation, comorbidity assessment, and treatment continuity through patient-carried anticoagulation documentation. Enhanced physician adherence to clinical guidelines and optimized treatment protocols have improved secondary prevention effectiveness, though their ASIR impact remains indirect [33, 34]. Conversely, individuals of lower socioeconomic status may be at higher risk for AF, particularly in low-income countries or less developed regions, where disease screening and treatment remain more challenging [33, 34].

Notable gender disparities persist in AF/AFL epidemiology across EU15+ nations, with males generally exhibiting higher ASIR and ASMR – consistent across age strata and aligned with previous findings [35]. However, incidence patterns reverse in senior populations (75–79 and 80+ years), where female rates surpass male counterparts. This epidemiological shift may reflect greater challenges in AF/AFL prevention and control in older women, such as the loss of estrogen protection after menopause, increased shortening of the atrial effective refractory period (ERP) during atrial pacing, and a moderate positive association between AF knowledge and life satisfaction, as well as a moderate negative association between AF knowledge and anxiety and depression, which are generally lower in men than in women. Women are more likely to develop left atrial appendage hypoplasia and atrial structural remodeling during the progression of AF, which increases their risk of stroke [36, 37, 38, 39]. European Heart Rhythm Association studies [40, 41] reveal gender-specific clinical presentations: women demonstrate higher hypertension/valvular heart disease comorbidity rates, more frequent atypical symptoms (dyspnea, chest pain, dizziness), and lower quality-of-life scores. Treatment disparities persist, with older women receiving fewer rhythm control interventions (catheter ablation, cardioversion) compared to pharmacological management.

Cross-national variations in AF/AFL burden highlight critical health system determinants. Finland’s significant ASIR/ASMR reductions align with its comprehensive Finnish anticoagulation in atrial fibrillation (FinACAF) registry, integrating primary care data, medication records, and socioeconomic parameters to optimize early detection and management [42]. Conversely, Sweden’s rising trends may reflect fragmented primary care documentation, where community-treated cases remain underrepresented in hospital registries [43]. Wealthier nations exhibit pronounced survivor effects, with extended longevity increasing AF diagnosis probability and severe sequelae. The observed ASIR-ASMR decoupling in Austria, Sweden, and the United States suggests therapeutic advances (particularly NOACs and ablation) may buffer mortality impacts despite growing disease burden.

This study employed age-period-cohort analysis to examine AF/AFL incidence trends across EU15+ countries. Ten nations demonstrated statistical significance: Austria, Belgium, Canada, Denmark, France, Greece, Spain, Sweden, the United Kingdom, and the United States. Over three decades, incidence rates showed a general decline across most countries except Austria, Sweden, and the United States, accompanied by a progressive shift in disease burden toward older populations (≥80 years). Sweden exhibited the highest ASIR values (males: 136.71/100,000; females: 109.47/100,000), while the lowest rates were observed in the United Kingdom and Belgium. These disparities appear attributable to intercountry variations in healthcare systems, demographic structures, socioeconomic factors, and environmental determinants, reflecting distinct national approaches to AF/AFL management.

The net drift analysis revealed an overall decreasing trend in EU15+ countries, with an increasing trend in some countries, such as Austria, Sweden, and the USA. This may be attributed to differences in the proportion of elderly populations, screening methods in healthcare systems, and variations in how patient data are collected in the GBD database. Additionally, the higher incidence of AF may be attributed to a more integrated AF care network, increased awareness, and better prevention efforts among healthcare professionals and high-risk individuals in these countries [44]. Local drift demonstrated changes in incidence rates across different age groups, with a general decline in incidence rates among older patients (≥75 years) in most countries, particularly a pronounced decline in men in the UK.

Some cohort studies have reported a higher incidence of anticoagulation prescriptions following the introduction of direct oral anticoagulants (DOACs) in AF/AFL patients aged ≥75 years in the UK, with success in managing AF/AFL risk in older age groups [45]. However, younger age groups (30–44 years) in countries such as Australia, Belgium, and Canada showed an increased incidence. This trend may be linked to lifestyle changes (e.g., obesity, smoking, alcohol consumption, poor dietary habits) in younger age groups, suggesting that preventive interventions may be insufficient for this population.

APC modeling of AF/AFL incidence reveals a rapid increase in incidence with age, particularly peaking in the 70–85 year age group, which may be associated with age-related deterioration of cardiovascular health and structural changes in the heart. However, there is a steady downward trend in incidence above the 75–85 year age group, possibly reflecting survival bias, where high-risk individuals have already experienced disease or death at younger ages. Additionally, a lack of definitive screening in higher age groups may contribute to the underestimation of incidence. Trends in the period effect reveal a downward trend in AF/AFL incidence across the seven countries from 1990 to 2021, with the most pronounced decline in France and the most notable increase in Austria. This difference in AF outcomes may relate to the distribution of risk factors across Europe, particularly the prevalence of modifiable risk factors (e.g., obesity, high alcohol intake, smoking, and physical inactivity) and common comorbidities (e.g., hypertension, diabetes mellitus, and coronary artery disease), which vary across regions.

Another possible explanation is that poorer healthcare systems often lack adequate diagnostic capacity and have limited access to screening tests, such as electrocardiograms, which may lead to errors in morbidity statistics, thus affecting the accurate assessment of disease burden. [46, 47] Cohort effects show higher morbidity in earlier birth cohorts (e.g., 1900s) compared with later cohorts (e.g., 1980s), likely associated with improved health interventions, socioeconomic conditions, health awareness, and changes in lifestyle habits over time. Earlier-born cohorts may face higher health risks due to past limitations in medical and socioeconomic conditions, contributing to the higher prevalence of AF/AFL. Future research should further explore the specific risk factors for AF/AFL in different countries, particularly the correlation with lifestyle, genetic diversity, and healthcare systems. A deeper understanding of these factors could help refine preventive measures and improve the management and treatment outcomes of AF/AFL.

## 5. Conclusions

This investigation presents a comprehensive evaluation of AF/AFL burden trajectories in high-income nations, systematically analyzing sex-stratified disparities in ASIR, ASMR, and MII from 1990 to 2021. The findings demonstrate substantial intercountry heterogeneity within EU15+ nations: while most exhibited declining ASIR and ASMR trends—strongly associated with therapeutic innovations including optimized anticoagulation protocols, antiarrhythmic agents, and radiofrequency ablation—However, variations between countries, such as the rising trends in Sweden and the USA, highlight the influence of healthcare systems, socioeconomic factors, and the accuracy of data reporting on disease burden assessments.

Sex-specific analysis revealed consistently elevated ASIR and ASMR among males overall, with a notable inversion in geriatric populations (≥75 years), particularly pronounced in females. These findings suggest that the progression of AF/AFL and its management may differ by gender, necessitating tailored approaches to prevention and treatment.

Despite therapeutic advancements, persistent challenges emerge in comorbid condition management and equitable access to novel therapies, particularly in resource-constrained settings. While widespread NOACs adoption and guideline-directed therapy implementation have reduced mortality burdens, enduring healthcare disparities necessitate targeted interventions to address system-level inequities.

## Availability of Data and Materials

The authors confirm that this study analyzed publicly available datasets. These data can be found here: the Global Burden of Disease (GBD) study (https://vizhub.healthdata.org/gbd-results/, accessed on 23 August 2024).

## Supplementary Material

Supplementary material associated with this article can be found, in the online version, at https://doi.org/10.31083/RCM36427.
