# Supplementary material for: Temporal Trend Analysis of Atrial Fibrillation/Flutter Disease Burden in High-Income Countries Between 1990 and 2021
Source: Rev Cardiovasc Med. 2025 Jul 25;26(7):36427. doi: 10.31083/RCM36427 (PMC12326443; doi:10.31083/RCM36427)
Supplement: Supplementary file 1 [file 2153-8174-26-7-36427-s1.zip › Supplementary Material 2.docx]

Table S3.Joinpoint analysis for Atrial fibrillation/flutter disease age-standardized incidence rates in EU15+ countries for years 1990–2021 in males.

| location | Model | Segment | Segment Start | Segment End | APC | APC 95% LCL | APC 95% UCL | P-Value |
| --- | --- | --- | --- | --- | --- | --- | --- | --- |
| Australia | 5 | 0 | 1990 | 2000 | 0.10 | 0.08 | 0.12 | 0 |
| Australia | 5 | 1 | 2000 | 2005 | -0.94 | -1.00 | -0.88 | 0 |
| Australia | 5 | 2 | 2005 | 2010 | 1.14 | 1.08 | 1.21 | 0 |
| Australia | 5 | 3 | 2010 | 2015 | -0.25 | -0.31 | -0.18 | 0 |
| Australia | 5 | 4 | 2015 | 2019 | 0.40 | 0.27 | 0.53 | 0 |
| Australia | 5 | 5 | 2019 | 2021 | -5.04 | -5.68 | -4.41 | 0 |
| Austria | 5 | 0 | 1990 | 2000 | 1.81 | 1.76 | 1.85 | 0 |
| Austria | 5 | 1 | 2000 | 2005 | 4.48 | 4.35 | 4.61 | 0 |
| Austria | 5 | 2 | 2005 | 2009 | 1.87 | 1.73 | 2.01 | 0 |
| Austria | 5 | 3 | 2009 | 2015 | 1.04 | 0.99 | 1.10 | 0 |
| Austria | 5 | 4 | 2015 | 2018 | 1.96 | 1.70 | 2.21 | 0 |
| Austria | 5 | 5 | 2018 | 2021 | 0.44 | 0.31 | 0.58 | 0 |
| Belgium | 5 | 0 | 1990 | 1995 | -0.02 | -0.07 | 0.04 | 0.577 |
| Belgium | 5 | 1 | 1995 | 2000 | -0.34 | -0.42 | -0.25 | 0 |
| Belgium | 5 | 2 | 2000 | 2005 | -2.83 | -2.90 | -2.75 | 0 |
| Belgium | 5 | 3 | 2005 | 2009 | 0.70 | 0.60 | 0.81 | 0 |
| Belgium | 5 | 4 | 2009 | 2019 | -0.18 | -0.20 | -0.16 | 0 |
| Belgium | 5 | 5 | 2019 | 2021 | 1.27 | 1.04 | 1.50 | 0 |
| Canada | 5 | 0 | 1990 | 1994 | -1.21 | -1.25 | -1.18 | 0 |
| Canada | 5 | 1 | 1994 | 2001 | -0.65 | -0.67 | -0.63 | 0 |
| Canada | 5 | 2 | 2001 | 2009 | -0.90 | -0.92 | -0.89 | 0 |
| Canada | 5 | 3 | 2009 | 2015 | -0.04 | -0.08 | -0.01 | 0.014 |
| Canada | 5 | 4 | 2015 | 2019 | 1.98 | 1.90 | 2.05 | 0 |
| Canada | 5 | 5 | 2019 | 2021 | -4.62 | -4.77 | -4.48 | 0 |
| Denmark | 5 | 0 | 1990 | 1993 | 5.98 | 5.24 | 6.72 | 0 |
| Denmark | 5 | 1 | 1993 | 1996 | 1.09 | 0.19 | 2.01 | 0.021 |
| Denmark | 5 | 2 | 1996 | 2003 | -0.46 | -0.62 | -0.30 | 0 |
| Denmark | 5 | 3 | 2003 | 2009 | -1.15 | -1.40 | -0.89 | 0 |
| Denmark | 5 | 4 | 2009 | 2019 | -0.86 | -0.97 | -0.75 | 0 |
| Denmark | 5 | 5 | 2019 | 2021 | -0.31 | -1.68 | 1.08 | 0.638 |
| Finland | 5 | 0 | 1990 | 1992 | 1.57 | 1.01 | 2.13 | 0 |
| Finland | 5 | 1 | 1992 | 1995 | 0.24 | -0.28 | 0.77 | 0.34 |
| Finland | 5 | 2 | 1995 | 2000 | -3.13 | -3.28 | -2.98 | 0 |
| Finland | 5 | 3 | 2000 | 2011 | -0.44 | -0.48 | -0.40 | 0 |
| Finland | 5 | 4 | 2011 | 2019 | -1.63 | -1.70 | -1.57 | 0 |
| Finland | 5 | 5 | 2019 | 2021 | 0.25 | -0.22 | 0.72 | 0.271 |
| France | 5 | 0 | 1990 | 1996 | -0.11 | -0.17 | -0.06 | 0 |
| France | 5 | 1 | 1996 | 2000 | -0.75 | -0.90 | -0.59 | 0 |
| France | 5 | 2 | 2000 | 2007 | -0.99 | -1.04 | -0.94 | 0 |
| France | 5 | 3 | 2007 | 2010 | -0.69 | -1.00 | -0.38 | 0 |
| France | 5 | 4 | 2010 | 2017 | -0.06 | -0.11 | 0.00 | 0.038 |
| France | 5 | 5 | 2017 | 2021 | 0.18 | 0.08 | 0.28 | 0.002 |
| Germany | 5 | 0 | 1990 | 1995 | 0.16 | -0.07 | 0.39 | 0.153 |
| Germany | 5 | 1 | 1995 | 1999 | 5.20 | 4.89 | 5.52 | 0 |
| Germany | 5 | 2 | 1999 | 2002 | -0.29 | -0.64 | 0.07 | 0.103 |
| Germany | 5 | 3 | 2002 | 2009 | -1.72 | -1.78 | -1.66 | 0 |
| Germany | 5 | 4 | 2009 | 2014 | -0.21 | -0.32 | -0.09 | 0.001 |
| Germany | 5 | 5 | 2014 | 2021 | 0.27 | 0.22 | 0.33 | 0 |
| Greece | 5 | 0 | 1990 | 1994 | 0.78 | 0.67 | 0.90 | 0 |
| Greece | 5 | 1 | 1994 | 2000 | -0.27 | -0.33 | -0.21 | 0 |
| Greece | 5 | 2 | 2000 | 2005 | 2.46 | 2.36 | 2.55 | 0 |
| Greece | 5 | 3 | 2005 | 2011 | -0.97 | -1.04 | -0.89 | 0 |
| Greece | 5 | 4 | 2011 | 2014 | -2.74 | -3.15 | -2.32 | 0 |
| Greece | 5 | 5 | 2014 | 2021 | -0.03 | -0.10 | 0.04 | 0.413 |
| Ireland | 5 | 0 | 1990 | 1995 | 0.27 | 0.14 | 0.40 | 0.001 |
| Ireland | 5 | 1 | 1995 | 2003 | -1.13 | -1.17 | -1.08 | 0 |
| Ireland | 5 | 2 | 2003 | 2010 | -1.28 | -1.33 | -1.23 | 0 |
| Ireland | 5 | 3 | 2010 | 2014 | -0.84 | -0.98 | -0.70 | 0 |
| Ireland | 5 | 4 | 2014 | 2019 | -0.02 | -0.11 | 0.07 | 0.666 |
| Ireland | 5 | 5 | 2019 | 2021 | 1.99 | 1.52 | 2.47 | 0 |
| Italy | 5 | 0 | 1990 | 1995 | -0.54 | -0.80 | -0.28 | 0 |
| Italy | 5 | 1 | 1995 | 2000 | -3.33 | -3.68 | -2.98 | 0 |
| Italy | 5 | 2 | 2000 | 2005 | -1.37 | -1.72 | -1.01 | 0 |
| Italy | 5 | 3 | 2005 | 2010 | 1.50 | 1.12 | 1.87 | 0 |
| Italy | 5 | 4 | 2010 | 2019 | -0.63 | -0.76 | -0.50 | 0 |
| Italy | 5 | 5 | 2019 | 2021 | 10.25 | 8.96 | 11.55 | 0 |
| Luxembourg | 5 | 0 | 1990 | 1992 | 0.02 | -0.24 | 0.28 | 0.894 |
| Luxembourg | 5 | 1 | 1992 | 1995 | 0.40 | 0.19 | 0.61 | 0.001 |
| Luxembourg | 5 | 2 | 1995 | 1999 | 0.92 | 0.83 | 1.00 | 0 |
| Luxembourg | 5 | 3 | 1999 | 2002 | -0.01 | -0.17 | 0.15 | 0.889 |
| Luxembourg | 5 | 4 | 2002 | 2010 | -0.25 | -0.27 | -0.23 | 0 |
| Luxembourg | 5 | 5 | 2010 | 2021 | 0.07 | 0.06 | 0.09 | 0 |
| Netherlands | 5 | 0 | 1990 | 1993 | 1.20 | 0.94 | 1.47 | 0 |
| Netherlands | 5 | 1 | 1993 | 1998 | 0.72 | 0.56 | 0.87 | 0 |
| Netherlands | 5 | 2 | 1998 | 2001 | -0.12 | -0.62 | 0.38 | 0.61 |
| Netherlands | 5 | 3 | 2001 | 2005 | -2.73 | -3.01 | -2.45 | 0 |
| Netherlands | 5 | 4 | 2005 | 2012 | -0.82 | -0.94 | -0.71 | 0 |
| Netherlands | 5 | 5 | 2012 | 2021 | 0.03 | -0.04 | 0.09 | 0.387 |
| Norway | 5 | 0 | 1990 | 1995 | -0.12 | -0.23 | 0.00 | 0.044 |
| Norway | 5 | 1 | 1995 | 2000 | -0.70 | -0.85 | -0.54 | 0 |
| Norway | 5 | 2 | 2000 | 2005 | 0.55 | 0.39 | 0.71 | 0 |
| Norway | 5 | 3 | 2005 | 2010 | -0.78 | -0.94 | -0.62 | 0 |
| Norway | 5 | 4 | 2010 | 2014 | -0.33 | -0.58 | -0.08 | 0.014 |
| Norway | 5 | 5 | 2014 | 2021 | 0.01 | -0.06 | 0.08 | 0.753 |
| Portugal | 5 | 0 | 1990 | 1995 | -0.54 | -0.66 | -0.42 | 0 |
| Portugal | 5 | 1 | 1995 | 2000 | -2.69 | -2.82 | -2.56 | 0 |
| Portugal | 5 | 2 | 2000 | 2004 | 0.92 | 0.77 | 1.08 | 0 |
| Portugal | 5 | 3 | 2004 | 2007 | 0.12 | -0.18 | 0.42 | 0.412 |
| Portugal | 5 | 4 | 2007 | 2015 | -0.14 | -0.18 | -0.10 | 0 |
| Portugal | 5 | 5 | 2015 | 2021 | 0.43 | 0.37 | 0.48 | 0 |
| Spain | 5 | 0 | 1990 | 1992 | -4.58 | -7.60 | -1.46 | 0.007 |
| Spain | 5 | 1 | 1992 | 1995 | -1.89 | -3.72 | -0.03 | 0.047 |
| Spain | 5 | 2 | 1995 | 2004 | 1.74 | 1.56 | 1.92 | 0 |
| Spain | 5 | 3 | 2004 | 2010 | 0.58 | 0.30 | 0.85 | 0 |
| Spain | 5 | 4 | 2010 | 2018 | -0.85 | -1.05 | -0.64 | 0 |
| Spain | 5 | 5 | 2018 | 2021 | 1.70 | 0.92 | 2.49 | 0 |
| Sweden | 5 | 0 | 1990 | 1994 | 2.55 | 2.37 | 2.74 | 0 |
| Sweden | 5 | 1 | 1994 | 2000 | 0.85 | 0.74 | 0.97 | 0 |
| Sweden | 5 | 2 | 2000 | 2006 | 1.14 | 1.03 | 1.24 | 0 |
| Sweden | 5 | 3 | 2006 | 2010 | 1.38 | 1.15 | 1.61 | 0 |
| Sweden | 5 | 4 | 2010 | 2019 | -0.03 | -0.08 | 0.02 | 0.272 |
| Sweden | 5 | 5 | 2019 | 2021 | 7.09 | 6.39 | 7.80 | 0 |
| United Kingdom | 5 | 0 | 1990 | 1998 | 0.19 | 0.14 | 0.24 | 0 |
| United Kingdom | 5 | 1 | 1998 | 2001 | -0.15 | -0.54 | 0.25 | 0.444 |
| United Kingdom | 5 | 2 | 2001 | 2010 | -1.16 | -1.20 | -1.12 | 0 |
| United Kingdom | 5 | 3 | 2010 | 2013 | 0.71 | 0.34 | 1.08 | 0.001 |
| United Kingdom | 5 | 4 | 2013 | 2019 | 0.95 | 0.86 | 1.04 | 0 |
| United Kingdom | 5 | 5 | 2019 | 2021 | 0.03 | -0.40 | 0.46 | 0.886 |
| United States of America | 5 | 0 | 1990 | 1994 | -2.98 | -3.15 | -2.81 | 0 |
| United States of America | 5 | 1 | 1994 | 2001 | 0.26 | 0.17 | 0.35 | 0 |
| United States of America | 5 | 2 | 2001 | 2004 | 2.93 | 2.62 | 3.24 | 0 |
| United States of America | 5 | 3 | 2004 | 2008 | 0.70 | 0.62 | 0.78 | 0 |
| United States of America | 5 | 4 | 2008 | 2019 | 0.19 | 0.18 | 0.20 | 0 |
| United States of America | 5 | 5 | 2019 | 2021 | 2.64 | 2.47 | 2.80 | 0 |

| location | Joinpoint Model | AAPC Index | Start Obs | End Obs | AAPC | AAPC C.I. Low | AAPC C.I. High | P-Value |
| --- | --- | --- | --- | --- | --- | --- | --- | --- |
| Australia | 5 | Full Range | 1990 | 2021 | -0.258 | -0.304 | -0.213 | 0 |
| Austria | 5 | Full Range | 1990 | 2021 | 1.974 | 1.934 | 2.014 | 0 |
| Belgium | 5 | Full Range | 1990 | 2021 | -0.403 | -0.43 | -0.377 | 0 |
| Canada | 5 | Full Range | 1990 | 2021 | -0.598 | -0.614 | -0.582 | 0 |
| Denmark | 5 | Full Range | 1990 | 2021 | 0.041 | -0.104 | 0.187 | 0.579 |
| Finland | 5 | Full Range | 1990 | 2021 | -0.95 | -1.02 | -0.881 | 0 |
| France | 5 | Full Range | 1990 | 2021 | -0.4 | -0.439 | -0.36 | 0 |
| Germany | 5 | Full Range | 1990 | 2021 | 0.289 | 0.226 | 0.352 | 0 |
| Greece | 5 | Full Range | 1990 | 2021 | -0.023 | -0.071 | 0.025 | 0.345 |
| Ireland | 5 | Full Range | 1990 | 2021 | -0.522 | -0.564 | -0.479 | 0 |
| Italy | 5 | Full Range | 1990 | 2021 | -0.171 | -0.298 | -0.043 | 0.009 |
| Luxembourg | 5 | Full Range | 1990 | 2021 | 0.117 | 0.087 | 0.147 | 0 |
| Netherlands | 5 | Full Range | 1990 | 2021 | -0.316 | -0.388 | -0.245 | 0 |
| Norway | 5 | Full Range | 1990 | 2021 | -0.208 | -0.263 | -0.154 | 0 |
| Portugal | 5 | Full Range | 1990 | 2021 | -0.35 | -0.394 | -0.306 | 0 |
| Spain | 5 | Full Range | 1990 | 2021 | 0.068 | -0.208 | 0.344 | 0.63 |
| Sweden | 5 | Full Range | 1990 | 2021 | 1.328 | 1.267 | 1.389 | 0 |
| United Kingdom | 5 | Full Range | 1990 | 2021 | -0.052 | -0.111 | 0.007 | 0.087 |
| United States of America | 5 | Full Range | 1990 | 2021 | 0.273 | 0.231 | 0.315 | 0 |

Table S4.Joinpoint analysis for Atrial fibrillation/flutter disease age-standardized incidence rates in EU15+ countries for years 1990–2021 in females.

| location | Model | Segment | Segment Start | Segment End | APC | APC 95% LCL | APC 95% UCL | P-Value |
| --- | --- | --- | --- | --- | --- | --- | --- | --- |
| Australia | 5 | 0 | 1990 | 1995 | 0.536 | 0.464 | 0.608 | 0 |
| Australia | 5 | 1 | 1995 | 2000 | 0.216 | 0.121 | 0.311 | 0 |
| Australia | 5 | 2 | 2000 | 2005 | -0.679 | -0.765 | -0.592 | 0 |
| Australia | 5 | 3 | 2005 | 2010 | 0.964 | 0.877 | 1.051 | 0 |
| Australia | 5 | 4 | 2010 | 2018 | 0.378 | 0.34 | 0.416 | 0 |
| Australia | 5 | 5 | 2018 | 2021 | 1.064 | 0.714 | 1.416 | 0 |
| Austria | 5 | 0 | 1990 | 2000 | 1.566 | 1.529 | 1.603 | 0 |
| Austria | 5 | 1 | 2000 | 2004 | 4.248 | 4.077 | 4.419 | 0 |
| Austria | 5 | 2 | 2004 | 2007 | 2.298 | 2.033 | 2.563 | 0 |
| Austria | 5 | 3 | 2007 | 2015 | 1.37 | 1.338 | 1.402 | 0 |
| Austria | 5 | 4 | 2015 | 2018 | 2.078 | 1.874 | 2.281 | 0 |
| Austria | 5 | 5 | 2018 | 2021 | 0.376 | 0.272 | 0.479 | 0 |
| Belgium | 3 | 0 | 1990 | 2000 | -0.22 | -0.27 | -0.169 | 0 |
| Belgium | 3 | 1 | 2000 | 2005 | -3.545 | -3.726 | -3.364 | 0 |
| Belgium | 3 | 2 | 2005 | 2019 | -0.11 | -0.139 | -0.081 | 0 |
| Belgium | 3 | 3 | 2019 | 2021 | 0.556 | -0.003 | 1.119 | 0.051 |
| Canada | 5 | 0 | 1990 | 1994 | -1.292 | -1.679 | -0.902 | 0 |
| Canada | 5 | 1 | 1994 | 2001 | -0.404 | -0.617 | -0.191 | 0.001 |
| Canada | 5 | 2 | 2001 | 2005 | -1.864 | -2.489 | -1.235 | 0 |
| Canada | 5 | 3 | 2005 | 2010 | -3.523 | -3.949 | -3.096 | 0 |
| Canada | 5 | 4 | 2010 | 2014 | 2.111 | 1.338 | 2.889 | 0 |
| Canada | 5 | 5 | 2014 | 2021 | -0.512 | -0.715 | -0.308 | 0 |
| Denmark | 5 | 0 | 1990 | 1994 | 3.192 | 2.961 | 3.422 | 0 |
| Denmark | 5 | 1 | 1994 | 2000 | -0.487 | -0.61 | -0.364 | 0 |
| Denmark | 5 | 2 | 2000 | 2005 | 0.54 | 0.36 | 0.72 | 0 |
| Denmark | 5 | 3 | 2005 | 2010 | -1.331 | -1.533 | -1.13 | 0 |
| Denmark | 5 | 4 | 2010 | 2019 | -0.755 | -0.831 | -0.679 | 0 |
| Denmark | 5 | 5 | 2019 | 2021 | 0.438 | -0.358 | 1.24 | 0.26 |
| Finland | 5 | 0 | 1990 | 1993 | 1.572 | 1.382 | 1.761 | 0 |
| Finland | 5 | 1 | 1993 | 1996 | -0.607 | -0.908 | -0.305 | 0.001 |
| Finland | 5 | 2 | 1996 | 1999 | -4.082 | -4.371 | -3.791 | 0 |
| Finland | 5 | 3 | 1999 | 2011 | -0.693 | -0.714 | -0.671 | 0 |
| Finland | 5 | 4 | 2011 | 2019 | -1.799 | -1.842 | -1.756 | 0 |
| Finland | 5 | 5 | 2019 | 2021 | 0.097 | -0.252 | 0.448 | 0.562 |
| France | 5 | 0 | 1990 | 1997 | -0.431 | -0.47 | -0.393 | 0 |
| France | 5 | 1 | 1997 | 2001 | -0.894 | -1.038 | -0.751 | 0 |
| France | 5 | 2 | 2001 | 2009 | -1.423 | -1.46 | -1.386 | 0 |
| France | 5 | 3 | 2009 | 2012 | -0.443 | -0.723 | -0.163 | 0.004 |
| France | 5 | 4 | 2012 | 2016 | -0.102 | -0.245 | 0.04 | 0.147 |
| France | 5 | 5 | 2016 | 2021 | 0.301 | 0.237 | 0.365 | 0 |
| Germany | 4 | 0 | 1990 | 1995 | -2.007 | -2.214 | -1.799 | 0 |
| Germany | 4 | 1 | 1995 | 2000 | 0.798 | 0.629 | 0.968 | 0 |
| Germany | 4 | 2 | 2000 | 2010 | -1.057 | -1.088 | -1.026 | 0 |
| Germany | 4 | 3 | 2010 | 2015 | 0.817 | 0.705 | 0.929 | 0 |
| Germany | 4 | 4 | 2015 | 2021 | 0.229 | 0.16 | 0.299 | 0 |
| Greece | 5 | 0 | 1990 | 1994 | 3.057 | 2.74 | 3.374 | 0 |
| Greece | 5 | 1 | 1994 | 2000 | -0.805 | -0.995 | -0.615 | 0 |
| Greece | 5 | 2 | 2000 | 2005 | 1.702 | 1.432 | 1.971 | 0 |
| Greece | 5 | 3 | 2005 | 2010 | -1.35 | -1.605 | -1.093 | 0 |
| Greece | 5 | 4 | 2010 | 2015 | -4.169 | -4.453 | -3.885 | 0 |
| Greece | 5 | 5 | 2015 | 2021 | 0.392 | 0.205 | 0.58 | 0 |
| Ireland | 4 | 0 | 1990 | 1996 | -0.409 | -0.595 | -0.222 | 0 |
| Ireland | 4 | 1 | 1996 | 2000 | -5.025 | -5.502 | -4.545 | 0 |
| Ireland | 4 | 2 | 2000 | 2011 | -1.444 | -1.516 | -1.373 | 0 |
| Ireland | 4 | 3 | 2011 | 2019 | -0.271 | -0.394 | -0.147 | 0 |
| Ireland | 4 | 4 | 2019 | 2021 | 7.996 | 6.882 | 9.122 | 0 |
| Italy | 2 | 0 | 1990 | 2015 | -0.827 | -0.864 | -0.791 | 0 |
| Italy | 2 | 1 | 2015 | 2019 | -0.191 | -1.126 | 0.752 | 0.679 |
| Italy | 2 | 2 | 2019 | 2021 | 4.082 | 2.123 | 6.079 | 0 |
| Luxembourg | 5 | 0 | 1990 | 1994 | -1.712 | -1.822 | -1.603 | 0 |
| Luxembourg | 5 | 1 | 1994 | 1997 | -0.618 | -0.864 | -0.371 | 0 |
| Luxembourg | 5 | 2 | 1997 | 2009 | -0.242 | -0.255 | -0.229 | 0 |
| Luxembourg | 5 | 3 | 2009 | 2015 | -0.654 | -0.69 | -0.617 | 0 |
| Luxembourg | 5 | 4 | 2015 | 2019 | 0.483 | 0.404 | 0.561 | 0 |
| Luxembourg | 5 | 5 | 2019 | 2021 | 0.157 | -0.044 | 0.358 | 0.117 |
| Netherlands | 5 | 0 | 1990 | 1993 | 1.207 | 0.921 | 1.494 | 0 |
| Netherlands | 5 | 1 | 1993 | 1998 | 0.453 | 0.275 | 0.631 | 0 |
| Netherlands | 5 | 2 | 1998 | 2001 | -0.331 | -0.902 | 0.243 | 0.237 |
| Netherlands | 5 | 3 | 2001 | 2005 | -3.086 | -3.458 | -2.713 | 0 |
| Netherlands | 5 | 4 | 2005 | 2010 | -1.578 | -1.878 | -1.276 | 0 |
| Netherlands | 5 | 5 | 2010 | 2021 | 0.146 | 0.08 | 0.213 | 0 |
| Norway | 5 | 0 | 1990 | 1995 | -0.574 | -0.627 | -0.522 | 0 |
| Norway | 5 | 1 | 1995 | 2000 | -0.713 | -0.788 | -0.639 | 0 |
| Norway | 5 | 2 | 2000 | 2005 | -0.24 | -0.314 | -0.167 | 0 |
| Norway | 5 | 3 | 2005 | 2010 | -1.51 | -1.582 | -1.438 | 0 |
| Norway | 5 | 4 | 2010 | 2014 | -0.385 | -0.499 | -0.27 | 0 |
| Norway | 5 | 5 | 2014 | 2021 | 0.094 | 0.063 | 0.124 | 0 |
| Portugal | 5 | 0 | 1990 | 1997 | -0.769 | -0.796 | -0.742 | 0 |
| Portugal | 5 | 1 | 1997 | 2000 | -0.249 | -0.374 | -0.123 | 0.001 |
| Portugal | 5 | 2 | 2000 | 2005 | 0.552 | 0.519 | 0.586 | 0 |
| Portugal | 5 | 3 | 2005 | 2010 | -0.408 | -0.442 | -0.374 | 0 |
| Portugal | 5 | 4 | 2010 | 2018 | -0.065 | -0.08 | -0.049 | 0 |
| Portugal | 5 | 5 | 2018 | 2021 | 0.101 | 0.03 | 0.173 | 0.009 |
| Spain | 5 | 0 | 1990 | 1995 | -1.658 | -1.962 | -1.353 | 0 |
| Spain | 5 | 1 | 1995 | 2000 | 2.641 | 2.363 | 2.92 | 0 |
| Spain | 5 | 2 | 2000 | 2005 | -1.783 | -2.04 | -1.524 | 0 |
| Spain | 5 | 3 | 2005 | 2010 | -0.127 | -0.355 | 0.102 | 0.256 |
| Spain | 5 | 4 | 2010 | 2015 | -1.716 | -1.985 | -1.446 | 0 |
| Spain | 5 | 5 | 2015 | 2021 | 0.051 | -0.126 | 0.229 | 0.55 |
| Sweden | 4 | 0 | 1990 | 1994 | 2.456 | 1.612 | 3.308 | 0 |
| Sweden | 4 | 1 | 1994 | 2001 | -1.683 | -2.027 | -1.337 | 0 |
| Sweden | 4 | 2 | 2001 | 2011 | 1.625 | 1.447 | 1.804 | 0 |
| Sweden | 4 | 3 | 2011 | 2019 | -0.029 | -0.297 | 0.24 | 0.825 |
| Sweden | 4 | 4 | 2019 | 2021 | 13.611 | 10.552 | 16.754 | 0 |
| United Kingdom | 5 | 0 | 1990 | 2000 | 0.301 | 0.256 | 0.347 | 0 |
| United Kingdom | 5 | 1 | 2000 | 2003 | -1.052 | -1.559 | -0.542 | 0.001 |
| United Kingdom | 5 | 2 | 2003 | 2010 | -1.753 | -1.833 | -1.671 | 0 |
| United Kingdom | 5 | 3 | 2010 | 2013 | 0.836 | 0.343 | 1.332 | 0.003 |
| United Kingdom | 5 | 4 | 2013 | 2019 | 1.331 | 1.211 | 1.452 | 0 |
| United Kingdom | 5 | 5 | 2019 | 2021 | -0.061 | -0.639 | 0.52 | 0.824 |
| United States of America | 5 | 0 | 1990 | 1994 | 2.865 | 2.638 | 3.092 | 0 |
| United States of America | 5 | 1 | 1994 | 2000 | 0.312 | 0.159 | 0.466 | 0.001 |
| United States of America | 5 | 2 | 2000 | 2005 | 1.56 | 1.44 | 1.679 | 0 |
| United States of America | 5 | 3 | 2005 | 2010 | -0.723 | -0.779 | -0.667 | 0 |
| United States of America | 5 | 4 | 2010 | 2019 | 0.304 | 0.286 | 0.322 | 0 |
| United States of America | 5 | 5 | 2019 | 2021 | 2.358 | 2.161 | 2.556 | 0 |

| location | Joinpoint Model | AAPC Index | Start Obs | End Obs | AAPC | AAPC C.I. Low | AAPC C.I. High | P-Value |
| --- | --- | --- | --- | --- | --- | --- | --- | --- |
| Australia | 5 | Full Range | 1990 | 2021 | 0.366 | 0.325 | 0.407 | 0 |
| Austria | 5 | Full Range | 1990 | 2021 | 1.861 | 1.822 | 1.901 | 0 |
| Belgium | 3 | Full Range | 1990 | 2021 | -0.665 | -0.713 | -0.616 | 0 |
| Canada | 5 | Full Range | 1990 | 2021 | -0.923 | -1.076 | -0.769 | 0 |
| Denmark | 5 | Full Range | 1990 | 2021 | -0.01 | -0.084 | 0.064 | 0.787 |
| Finland | 5 | Full Range | 1990 | 2021 | -1.037 | -1.085 | -0.989 | 0 |
| France | 5 | Full Range | 1990 | 2021 | -0.589 | -0.627 | -0.552 | 0 |
| Germany | 4 | Full Range | 1990 | 2021 | -0.365 | -0.412 | -0.319 | 0 |
| Greece | 5 | Full Range | 1990 | 2021 | -0.326 | -0.418 | -0.233 | 0 |
| Ireland | 4 | Full Range | 1990 | 2021 | -0.831 | -0.931 | -0.731 | 0 |
| Italy | 2 | Full Range | 1990 | 2021 | -0.436 | -0.601 | -0.27 | 0 |
| Luxembourg | 5 | Full Range | 1990 | 2021 | -0.43 | -0.461 | -0.4 | 0 |
| Netherlands | 5 | Full Range | 1990 | 2021 | -0.451 | -0.543 | -0.359 | 0 |
| Norway | 5 | Full Range | 1990 | 2021 | -0.52 | -0.545 | -0.494 | 0 |
| Portugal | 5 | Full Range | 1990 | 2021 | -0.182 | -0.198 | -0.166 | 0 |
| Spain | 5 | Full Range | 1990 | 2021 | -0.428 | -0.523 | -0.333 | 0 |
| Sweden | 4 | Full Range | 1990 | 2021 | 1.274 | 1.049 | 1.499 | 0 |
| United Kingdom | 5 | Full Range | 1990 | 2021 | -0.072 | -0.15 | 0.006 | 0.071 |
| United States of America | 5 | Full Range | 1990 | 2021 | 0.799 | 0.755 | 0.844 | 0 |

Table S5. Joinpoint analysis for Atrial fibrillation/flutter disease age-standardized mortality rates in EU15+ countries for years 1990–2021 in males.

| location | Model | Segment | Segment Start | Segment End | APC | APC 95% LCL | APC 95% UCL | P-Value |
| --- | --- | --- | --- | --- | --- | --- | --- | --- |
| Australia | 5 | 0 | 1990 | 1995 | 0.7524 | 0.0949 | 1.4143 | 0.027573 |
| Australia | 5 | 1 | 1995 | 2000 | 0.1459 | -0.8703 | 1.1725 | 0.764769 |
| Australia | 5 | 2 | 2000 | 2003 | 2.5748 | -1.1453 | 6.435 | 0.163075 |
| Australia | 5 | 3 | 2003 | 2008 | -0.4524 | -1.6659 | 0.7761 | 0.442993 |
| Australia | 5 | 4 | 2008 | 2015 | -0.9398 | -1.6151 | -0.2599 | 0.010087 |
| Australia | 5 | 5 | 2015 | 2021 | -2.3666 | -3.1221 | -1.6052 | 0.000009 |
| Austria | 5 | 0 | 1990 | 1995 | 1.5728 | 0.7123 | 2.4406 | 0.001394 |
| Austria | 5 | 1 | 1995 | 1998 | 4.8116 | 0.7434 | 9.044 | 0.023084 |
| Austria | 5 | 2 | 1998 | 2003 | 2.5081 | 1.0896 | 3.9465 | 0.001782 |
| Austria | 5 | 3 | 2003 | 2010 | 0.9828 | 0.1795 | 1.7924 | 0.019694 |
| Austria | 5 | 4 | 2010 | 2014 | -3.1249 | -5.5699 | -0.6165 | 0.018296 |
| Austria | 5 | 5 | 2014 | 2021 | 0.0144 | -0.704 | 0.738 | 0.966587 |
| Belgium | 5 | 0 | 1990 | 1993 | 0.2094 | -1.5128 | 1.9617 | 0.800536 |
| Belgium | 5 | 1 | 1993 | 1996 | -1.0632 | -4.5961 | 2.6007 | 0.540393 |
| Belgium | 5 | 2 | 1996 | 2000 | 5.7548 | 3.9271 | 7.6146 | 0.000006 |
| Belgium | 5 | 3 | 2000 | 2004 | -2.8546 | -4.5817 | -1.0963 | 0.003637 |
| Belgium | 5 | 4 | 2004 | 2016 | 0.5331 | 0.268 | 0.799 | 0.000643 |
| Belgium | 5 | 5 | 2016 | 2021 | -3.0625 | -4.0777 | -2.0365 | 0.000014 |
| Canada | 5 | 0 | 1990 | 1992 | -0.936 | -2.0551 | 0.1959 | 0.098039 |
| Canada | 5 | 1 | 1992 | 1999 | 0.0274 | -0.1656 | 0.2209 | 0.766201 |
| Canada | 5 | 2 | 1999 | 2002 | -0.3502 | -1.5384 | 0.8523 | 0.542394 |
| Canada | 5 | 3 | 2002 | 2015 | -0.7899 | -0.8706 | -0.7091 | 0 |
| Canada | 5 | 4 | 2015 | 2019 | 0.2988 | -0.4576 | 1.0609 | 0.414068 |
| Canada | 5 | 5 | 2019 | 2021 | -3.0935 | -4.7062 | -1.4537 | 0.00118 |
| Denmark | 5 | 0 | 1990 | 1996 | 3.3547 | 2.5574 | 4.1582 | 0 |
| Denmark | 5 | 1 | 1996 | 2000 | 4.5844 | 2.4311 | 6.783 | 0.000352 |
| Denmark | 5 | 2 | 2000 | 2011 | 1.546 | 1.1633 | 1.9301 | 0 |
| Denmark | 5 | 3 | 2011 | 2016 | -0.8355 | -2.5547 | 0.9139 | 0.322699 |
| Denmark | 5 | 4 | 2016 | 2019 | -4.3492 | -9.4339 | 1.0209 | 0.103214 |
| Denmark | 5 | 5 | 2019 | 2021 | -0.2812 | -5.7912 | 5.551 | 0.917306 |
| Finland | 5 | 0 | 1990 | 1994 | -0.8574 | -1.7502 | 0.0435 | 0.060585 |
| Finland | 5 | 1 | 1994 | 1998 | 1.4926 | 0.123 | 2.881 | 0.034564 |
| Finland | 5 | 2 | 1998 | 2006 | 0.0334 | -0.3242 | 0.3924 | 0.844989 |
| Finland | 5 | 3 | 2006 | 2009 | -5.7891 | -8.4468 | -3.0544 | 0.000475 |
| Finland | 5 | 4 | 2009 | 2014 | -0.9925 | -1.9378 | -0.038 | 0.042579 |
| Finland | 5 | 5 | 2014 | 2021 | -1.8271 | -2.2594 | -1.393 | 0 |
| France | 5 | 0 | 1990 | 1994 | 0.1112 | -0.2959 | 0.52 | 0.569739 |
| France | 5 | 1 | 1994 | 1997 | 0.6943 | -0.5824 | 1.9875 | 0.265868 |
| France | 5 | 2 | 1997 | 2002 | -0.6206 | -1.0394 | -0.2001 | 0.006712 |
| France | 5 | 3 | 2002 | 2009 | -1.4061 | -1.6512 | -1.1604 | 0 |
| France | 5 | 4 | 2009 | 2018 | 0.2385 | 0.0597 | 0.4177 | 0.01231 |
| France | 5 | 5 | 2018 | 2021 | -2.4842 | -3.4161 | -1.5433 | 0.000052 |
| Germany | 5 | 0 | 1990 | 2000 | -0.7026 | -0.8715 | -0.5335 | 0 |
| Germany | 5 | 1 | 2000 | 2004 | 6.5594 | 5.3743 | 7.7579 | 0 |
| Germany | 5 | 2 | 2004 | 2007 | 1.3443 | -1.0302 | 3.7758 | 0.248572 |
| Germany | 5 | 3 | 2007 | 2010 | -1.4238 | -3.8306 | 1.0433 | 0.235278 |
| Germany | 5 | 4 | 2010 | 2017 | 1.4927 | 1.0619 | 1.9254 | 0.000002 |
| Germany | 5 | 5 | 2017 | 2021 | -2.0014 | -2.906 | -1.0884 | 0.000316 |
| Greece | 5 | 0 | 1990 | 1993 | 0.2934 | -0.9199 | 1.5215 | 0.615408 |
| Greece | 5 | 1 | 1993 | 1996 | 1.3956 | -0.9993 | 3.8485 | 0.235515 |
| Greece | 5 | 2 | 1996 | 2002 | -1.642 | -2.1862 | -1.0948 | 0.000013 |
| Greece | 5 | 3 | 2002 | 2007 | 0.7961 | -0.0246 | 1.6235 | 0.056414 |
| Greece | 5 | 4 | 2007 | 2010 | -0.8607 | -3.543 | 1.8962 | 0.511981 |
| Greece | 5 | 5 | 2010 | 2021 | 0.4112 | 0.2087 | 0.6141 | 0.000593 |
| Ireland | 5 | 0 | 1990 | 1999 | 1.8006 | 1.2016 | 2.4031 | 0.000011 |
| Ireland | 5 | 1 | 1999 | 2002 | -3.8858 | -10.2952 | 2.9816 | 0.239807 |
| Ireland | 5 | 2 | 2002 | 2005 | 3.9433 | -3.5825 | 12.0565 | 0.290014 |
| Ireland | 5 | 3 | 2005 | 2008 | -7.9189 | -15.0251 | -0.2186 | 0.044782 |
| Ireland | 5 | 4 | 2008 | 2015 | 2.1109 | 0.5903 | 3.6546 | 0.009585 |
| Ireland | 5 | 5 | 2015 | 2021 | -3.2643 | -5.0282 | -1.4677 | 0.001593 |
| Italy | 5 | 0 | 1990 | 1993 | -1.1349 | -2.9048 | 0.6674 | 0.198092 |
| Italy | 5 | 1 | 1993 | 2001 | 0.1235 | -0.3924 | 0.6422 | 0.617917 |
| Italy | 5 | 2 | 2001 | 2006 | 1.909 | 0.6159 | 3.2187 | 0.006522 |
| Italy | 5 | 3 | 2006 | 2009 | 4.1714 | -0.1626 | 8.6936 | 0.058288 |
| Italy | 5 | 4 | 2009 | 2016 | 2.3461 | 1.5609 | 3.1374 | 0.000012 |
| Italy | 5 | 5 | 2016 | 2021 | -0.9503 | -2.0824 | 0.1949 | 0.096976 |
| Luxembourg | 5 | 0 | 1990 | 1997 | -0.4864 | -0.8349 | -0.1366 | 0.009698 |
| Luxembourg | 5 | 1 | 1997 | 2004 | 1.894 | 1.3631 | 2.4278 | 0.000001 |
| Luxembourg | 5 | 2 | 2004 | 2010 | 0.6107 | -0.1197 | 1.3464 | 0.095142 |
| Luxembourg | 5 | 3 | 2010 | 2013 | -0.6055 | -3.8633 | 2.7626 | 0.703116 |
| Luxembourg | 5 | 4 | 2013 | 2016 | 1.7194 | -1.6883 | 5.2451 | 0.303122 |
| Luxembourg | 5 | 5 | 2016 | 2021 | -3.0934 | -3.9695 | -2.2092 | 0.000002 |
| Netherlands | 5 | 0 | 1990 | 1996 | 0.7918 | 0.5235 | 1.0608 | 0.000014 |
| Netherlands | 5 | 1 | 1996 | 2000 | -1.8633 | -2.6525 | -1.0678 | 0.000169 |
| Netherlands | 5 | 2 | 2000 | 2010 | -1.1045 | -1.2608 | -0.948 | 0 |
| Netherlands | 5 | 3 | 2010 | 2016 | 0.8432 | 0.4132 | 1.2751 | 0.000792 |
| Netherlands | 5 | 4 | 2016 | 2019 | -0.4908 | -2.3566 | 1.4106 | 0.587711 |
| Netherlands | 5 | 5 | 2019 | 2021 | 1.5661 | -0.3525 | 3.5216 | 0.102908 |
| Norway | 5 | 0 | 1990 | 1993 | 9.3414 | 3.9268 | 15.0381 | 0.001939 |
| Norway | 5 | 1 | 1993 | 1997 | -4.9647 | -9.9195 | 0.2626 | 0.060812 |
| Norway | 5 | 2 | 1997 | 2001 | 2.657 | -2.754 | 8.3691 | 0.318328 |
| Norway | 5 | 3 | 2001 | 2004 | -3.3775 | -14.3435 | 8.9923 | 0.552329 |
| Norway | 5 | 4 | 2004 | 2012 | 1.509 | -0.2461 | 3.295 | 0.087145 |
| Norway | 5 | 5 | 2012 | 2021 | -3.4574 | -4.7952 | -2.1008 | 0.000077 |
| Portugal | 5 | 0 | 1990 | 1997 | -0.4158 | -0.8026 | -0.0275 | 0.037488 |
| Portugal | 5 | 1 | 1997 | 2000 | 1.0213 | -1.8684 | 3.9962 | 0.467034 |
| Portugal | 5 | 2 | 2000 | 2003 | -0.1406 | -3.1281 | 2.939 | 0.922646 |
| Portugal | 5 | 3 | 2003 | 2011 | -3.0324 | -3.496 | -2.5666 | 0 |
| Portugal | 5 | 4 | 2011 | 2014 | 2.0851 | -1.987 | 6.3264 | 0.29697 |
| Portugal | 5 | 5 | 2014 | 2021 | 0.11 | -0.4868 | 0.7104 | 0.700613 |
| Spain | 5 | 0 | 1990 | 1994 | -1.5143 | -2.5989 | -0.4176 | 0.010201 |
| Spain | 5 | 1 | 1994 | 1999 | 0.377 | -0.7397 | 1.5063 | 0.484439 |
| Spain | 5 | 2 | 1999 | 2007 | -0.8363 | -1.3469 | -0.323 | 0.003448 |
| Spain | 5 | 3 | 2007 | 2010 | 2.6982 | -1.5143 | 7.091 | 0.195497 |
| Spain | 5 | 4 | 2010 | 2019 | 0.4698 | -0.0107 | 0.9527 | 0.0547 |
| Spain | 5 | 5 | 2019 | 2021 | -2.9733 | -7.4424 | 1.7115 | 0.192575 |
| Sweden | 5 | 0 | 1990 | 1996 | 3.6746 | 2.4746 | 4.8886 | 0.000008 |
| Sweden | 5 | 1 | 1996 | 2002 | 0.0377 | -1.6619 | 1.7666 | 0.963249 |
| Sweden | 5 | 2 | 2002 | 2006 | 13.4764 | 8.8744 | 18.273 | 0.00001 |
| Sweden | 5 | 3 | 2006 | 2009 | -4.0929 | -11.8676 | 4.3676 | 0.308725 |
| Sweden | 5 | 4 | 2009 | 2016 | 2.6434 | 1.0794 | 4.2316 | 0.002511 |
| Sweden | 5 | 5 | 2016 | 2021 | -1.0334 | -3.5074 | 1.504 | 0.395583 |
| United Kingdom | 5 | 0 | 1990 | 1994 | -1.1738 | -1.6338 | -0.7117 | 0.000074 |
| United Kingdom | 5 | 1 | 1994 | 1999 | 0.8219 | 0.3592 | 1.2867 | 0.001768 |
| United Kingdom | 5 | 2 | 1999 | 2005 | -0.6315 | -0.9966 | -0.265 | 0.002283 |
| United Kingdom | 5 | 3 | 2005 | 2011 | -0.5065 | -0.9369 | -0.0743 | 0.024661 |
| United Kingdom | 5 | 4 | 2011 | 2018 | 2.1946 | 1.8239 | 2.5665 | 0 |
| United Kingdom | 5 | 5 | 2018 | 2021 | -1.9922 | -3.0854 | -0.8866 | 0.001661 |
| United States of America | 5 | 0 | 1990 | 1997 | 0.8924 | 0.7151 | 1.0699 | 0 |
| United States of America | 5 | 1 | 1997 | 2000 | 1.6486 | 0.2956 | 3.0199 | 0.020065 |
| United States of America | 5 | 2 | 2000 | 2005 | -0.6777 | -1.1066 | -0.2469 | 0.004393 |
| United States of America | 5 | 3 | 2005 | 2015 | 0.8216 | 0.6888 | 0.9546 | 0 |
| United States of America | 5 | 4 | 2015 | 2018 | 1.1368 | -0.4063 | 2.7038 | 0.137938 |
| United States of America | 5 | 5 | 2018 | 2021 | 0.224 | -0.5861 | 1.0408 | 0.565441 |

| location | Joinpoint Model | AAPC Index | Start Obs | End Obs | AAPC | AAPC C.I. Low | AAPC C.I. High | P-Value |
| --- | --- | --- | --- | --- | --- | --- | --- | --- |
| Australia | 5 | Full Range | 1990 | 2021 | -0.3588 | -0.8174 | 0.1018 | 0.126645 |
| Austria | 5 | Full Range | 1990 | 2021 | 0.9247 | 0.3513 | 1.5014 | 0.001544 |
| Belgium | 5 | Full Range | 1990 | 2021 | -0.0308 | -0.5292 | 0.4701 | 0.903943 |
| Canada | 5 | Full Range | 1990 | 2021 | -0.5835 | -0.7733 | -0.3934 | 0 |
| Denmark | 5 | Full Range | 1990 | 2021 | 1.1845 | 0.4628 | 1.9114 | 0.001263 |
| Finland | 5 | Full Range | 1990 | 2021 | -1.06 | -1.4272 | -0.6915 | 0 |
| France | 5 | Full Range | 1990 | 2021 | -0.5118 | -0.688 | -0.3354 | 0 |
| Germany | 5 | Full Range | 1990 | 2021 | 0.6586 | 0.2923 | 1.0264 | 0.000418 |
| Greece | 5 | Full Range | 1990 | 2021 | 0.0319 | -0.3488 | 0.414 | 0.869841 |
| Ireland | 5 | Full Range | 1990 | 2021 | -0.4592 | -1.6964 | 0.7937 | 0.470805 |
| Italy | 5 | Full Range | 1990 | 2021 | 0.9965 | 0.4688 | 1.5269 | 0.000207 |
| Luxembourg | 5 | Full Range | 1990 | 2021 | 0.0308 | -0.4497 | 0.5137 | 0.900183 |
| Netherlands | 5 | Full Range | 1990 | 2021 | -0.2329 | -0.4782 | 0.013 | 0.063337 |
| Norway | 5 | Full Range | 1990 | 2021 | -0.4211 | -1.9769 | 1.1595 | 0.59947 |
| Portugal | 5 | Full Range | 1990 | 2021 | -0.5778 | -1.1262 | -0.0265 | 0.039998 |
| Spain | 5 | Full Range | 1990 | 2021 | -0.1538 | -0.6931 | 0.3885 | 0.577561 |
| Sweden | 5 | Full Range | 1990 | 2021 | 2.3821 | 1.2733 | 3.5031 | 0.000023 |
| United Kingdom | 5 | Full Range | 1990 | 2021 | 0.0542 | -0.1291 | 0.2378 | 0.562222 |
| United States of America | 5 | Full Range | 1990 | 2021 | 0.6463 | 0.4327 | 0.8603 | 0 |

Table S6. Joinpoint analysis for Atrial fibrillation/flutter disease age-standardized mortality rates in EU15+ countries for years 1990–2021 in females.

| location | Model | Segment | Segment Start | Segment End | APC | APC 95% LCL | APC 95% UCL | P-Value |
| --- | --- | --- | --- | --- | --- | --- | --- | --- |
| Australia | 5 | 0 | 1990 | 1996 | 0.5308 | 0.1221 | 0.9412 | 0.0143 |
| Australia | 5 | 1 | 1996 | 2001 | -0.1542 | -0.9665 | 0.6648 | 0.692909 |
| Australia | 5 | 2 | 2001 | 2004 | 1.5786 | -1.2115 | 4.4476 | 0.249259 |
| Australia | 5 | 3 | 2004 | 2009 | 0.5446 | -0.3843 | 1.4823 | 0.231433 |
| Australia | 5 | 4 | 2009 | 2015 | 0.0191 | -0.6873 | 0.7304 | 0.955024 |
| Australia | 5 | 5 | 2015 | 2021 | -2.4869 | -3.0628 | -1.9076 | 0 |
| Austria | 5 | 0 | 1990 | 1995 | 1.2382 | 0.7282 | 1.7507 | 0.000109 |
| Austria | 5 | 1 | 1995 | 1998 | 4.3913 | 1.9278 | 6.9144 | 0.001621 |
| Austria | 5 | 2 | 1998 | 2010 | 1.3538 | 1.1696 | 1.5382 | 0 |
| Austria | 5 | 3 | 2010 | 2015 | -3.3968 | -4.2969 | -2.4883 | 0.000001 |
| Austria | 5 | 4 | 2015 | 2019 | 1.4626 | -0.1237 | 3.0741 | 0.068343 |
| Austria | 5 | 5 | 2019 | 2021 | -2.0719 | -5.3715 | 1.3427 | 0.212554 |
| Belgium | 5 | 0 | 1990 | 1996 | -0.0723 | -0.9266 | 0.7893 | 0.859826 |
| Belgium | 5 | 1 | 1996 | 1999 | 5.8233 | 0.3649 | 11.5786 | 0.037792 |
| Belgium | 5 | 2 | 1999 | 2005 | -1.2585 | -2.4452 | -0.0574 | 0.041234 |
| Belgium | 5 | 3 | 2005 | 2010 | 1.0132 | -0.8299 | 2.8905 | 0.261495 |
| Belgium | 5 | 4 | 2010 | 2016 | -0.4253 | -1.8108 | 0.9799 | 0.526623 |
| Belgium | 5 | 5 | 2016 | 2021 | -3.778 | -5.2369 | -2.2968 | 0.000078 |
| Canada | 5 | 0 | 1990 | 1995 | 3.1241 | 2.8925 | 3.3563 | 0 |
| Canada | 5 | 1 | 1995 | 2005 | -0.9446 | -1.0399 | -0.8492 | 0 |
| Canada | 5 | 2 | 2005 | 2011 | -2.8397 | -3.1018 | -2.5768 | 0 |
| Canada | 5 | 3 | 2011 | 2015 | 0.1197 | -0.5162 | 0.7597 | 0.694713 |
| Canada | 5 | 4 | 2015 | 2019 | 1.4965 | 0.8409 | 2.1565 | 0.000198 |
| Canada | 5 | 5 | 2019 | 2021 | -2.7102 | -4.0507 | -1.351 | 0.00074 |
| Denmark | 5 | 0 | 1990 | 1992 | 1.4265 | -2.1925 | 5.1793 | 0.419045 |
| Denmark | 5 | 1 | 1992 | 1995 | 4.1933 | 0.6888 | 7.8197 | 0.0218 |
| Denmark | 5 | 2 | 1995 | 2004 | 3.0143 | 2.5775 | 3.4529 | 0 |
| Denmark | 5 | 3 | 2004 | 2011 | 0.4485 | -0.2602 | 1.1622 | 0.197897 |
| Denmark | 5 | 4 | 2011 | 2014 | -6.2929 | -10.2895 | -2.1183 | 0.006233 |
| Denmark | 5 | 5 | 2014 | 2021 | -1.3681 | -2.0052 | -0.7268 | 0.000398 |
| Finland | 5 | 0 | 1990 | 2001 | 0.3739 | 0.1839 | 0.5642 | 0.000775 |
| Finland | 5 | 1 | 2001 | 2007 | -2.1127 | -2.7415 | -1.4799 | 0.000004 |
| Finland | 5 | 2 | 2007 | 2010 | -8.0511 | -10.7966 | -5.221 | 0.000029 |
| Finland | 5 | 3 | 2010 | 2014 | -0.345 | -1.9146 | 1.2497 | 0.649306 |
| Finland | 5 | 4 | 2014 | 2017 | -5.3866 | -8.3482 | -2.3293 | 0.002091 |
| Finland | 5 | 5 | 2017 | 2021 | -2.1105 | -3.1245 | -1.0859 | 0.000553 |
| France | 5 | 0 | 1990 | 1996 | -0.2732 | -0.4851 | -0.0609 | 0.015123 |
| France | 5 | 1 | 1996 | 2003 | -0.4462 | -0.6705 | -0.2214 | 0.000733 |
| France | 5 | 2 | 2003 | 2006 | -1.503 | -2.9392 | -0.0455 | 0.044107 |
| France | 5 | 3 | 2006 | 2011 | -0.8787 | -1.3616 | -0.3935 | 0.001566 |
| France | 5 | 4 | 2011 | 2016 | 0.7673 | 0.2293 | 1.3083 | 0.008216 |
| France | 5 | 5 | 2016 | 2021 | -2.0691 | -2.4615 | -1.675 | 0 |
| Germany | 5 | 0 | 1990 | 1997 | -0.2498 | -0.5777 | 0.0793 | 0.1264 |
| Germany | 5 | 1 | 1997 | 2000 | -1.3015 | -3.7331 | 1.1915 | 0.280581 |
| Germany | 5 | 2 | 2000 | 2004 | 6.0144 | 4.6706 | 7.3754 | 0 |
| Germany | 5 | 3 | 2004 | 2014 | 0.3913 | 0.147 | 0.6362 | 0.003823 |
| Germany | 5 | 4 | 2014 | 2018 | 1.6721 | 0.1985 | 3.1674 | 0.028629 |
| Germany | 5 | 5 | 2018 | 2021 | -2.9114 | -4.4941 | -1.3025 | 0.001634 |
| Greece | 5 | 0 | 1990 | 1993 | 1.5518 | 0.0879 | 3.037 | 0.039093 |
| Greece | 5 | 1 | 1993 | 2000 | -0.6289 | -1.1283 | -0.1271 | 0.017493 |
| Greece | 5 | 2 | 2000 | 2006 | 1.1592 | 0.5118 | 1.8108 | 0.001653 |
| Greece | 5 | 3 | 2006 | 2010 | -3.3643 | -4.915 | -1.7883 | 0.000416 |
| Greece | 5 | 4 | 2010 | 2013 | 2.069 | -1.5401 | 5.8103 | 0.244074 |
| Greece | 5 | 5 | 2013 | 2021 | -1.033 | -1.4545 | -0.6097 | 0.000111 |
| Ireland | 5 | 0 | 1990 | 1999 | 1.2113 | 0.749 | 1.6758 | 0.00005 |
| Ireland | 5 | 1 | 1999 | 2005 | -1.3308 | -2.595 | -0.0502 | 0.042705 |
| Ireland | 5 | 2 | 2005 | 2010 | -2.6191 | -4.6507 | -0.5441 | 0.017029 |
| Ireland | 5 | 3 | 2010 | 2013 | 7.0483 | -0.2854 | 14.9215 | 0.058748 |
| Ireland | 5 | 4 | 2013 | 2017 | 0.9098 | -2.7163 | 4.671 | 0.605569 |
| Ireland | 5 | 5 | 2017 | 2021 | -5.7961 | -8.4204 | -3.0966 | 0.00042 |
| Italy | 5 | 0 | 1990 | 1998 | -0.8896 | -1.1611 | -0.6172 | 0.000005 |
| Italy | 5 | 1 | 1998 | 2001 | -2.0002 | -4.6234 | 0.6952 | 0.133321 |
| Italy | 5 | 2 | 2001 | 2006 | 0.0933 | -0.8236 | 1.0187 | 0.831824 |
| Italy | 5 | 3 | 2006 | 2009 | 4.9878 | 1.7927 | 8.2831 | 0.004324 |
| Italy | 5 | 4 | 2009 | 2017 | 0.7413 | 0.3071 | 1.1774 | 0.002397 |
| Italy | 5 | 5 | 2017 | 2021 | -1.0835 | -2.1614 | 0.0062 | 0.051147 |
| Luxembourg | 5 | 0 | 1990 | 1992 | 2.0258 | -0.3171 | 4.4239 | 0.085636 |
| Luxembourg | 5 | 1 | 1992 | 2000 | -0.5752 | -0.9291 | -0.2201 | 0.00358 |
| Luxembourg | 5 | 2 | 2000 | 2004 | 3.5049 | 1.968 | 5.065 | 0.000189 |
| Luxembourg | 5 | 3 | 2004 | 2011 | -1.5975 | -2.1294 | -1.0627 | 0.000013 |
| Luxembourg | 5 | 4 | 2011 | 2015 | 3.6655 | 1.9839 | 5.3748 | 0.000289 |
| Luxembourg | 5 | 5 | 2015 | 2021 | -3.2781 | -3.9169 | -2.6351 | 0 |
| Netherlands | 5 | 0 | 1990 | 1993 | 1.3952 | 0.5128 | 2.2853 | 0.004133 |
| Netherlands | 5 | 1 | 1993 | 1996 | -0.0419 | -1.7371 | 1.6825 | 0.959021 |
| Netherlands | 5 | 2 | 1996 | 1999 | -2.8149 | -4.5167 | -1.0828 | 0.003609 |
| Netherlands | 5 | 3 | 1999 | 2009 | -1.4913 | -1.6567 | -1.3257 | 0 |
| Netherlands | 5 | 4 | 2009 | 2017 | 0.5437 | 0.2642 | 0.8239 | 0.000852 |
| Netherlands | 5 | 5 | 2017 | 2021 | -0.9002 | -1.5645 | -0.2315 | 0.01178 |
| Norway | 5 | 0 | 1990 | 1994 | 3.4659 | 0.1563 | 6.8849 | 0.041144 |
| Norway | 5 | 1 | 1994 | 1997 | -4.4119 | -14.297 | 6.6134 | 0.392193 |
| Norway | 5 | 2 | 1997 | 2001 | 2.9238 | -2.6111 | 8.7732 | 0.28396 |
| Norway | 5 | 3 | 2001 | 2004 | -2.1018 | -12.4317 | 9.4467 | 0.690451 |
| Norway | 5 | 4 | 2004 | 2013 | 1.214 | -0.1949 | 2.6429 | 0.086438 |
| Norway | 5 | 5 | 2013 | 2021 | -2.9262 | -4.4255 | -1.4033 | 0.001013 |
| Portugal | 5 | 0 | 1990 | 1994 | -0.6013 | -1.1464 | -0.0532 | 0.033671 |
| Portugal | 5 | 1 | 1994 | 2004 | -0.1181 | -0.2933 | 0.0575 | 0.172091 |
| Portugal | 5 | 2 | 2004 | 2009 | -5.2055 | -5.8892 | -4.5168 | 0 |
| Portugal | 5 | 3 | 2009 | 2012 | -1.2741 | -3.6862 | 1.1985 | 0.286611 |
| Portugal | 5 | 4 | 2012 | 2015 | 2.9443 | 0.289 | 5.67 | 0.031821 |
| Portugal | 5 | 5 | 2015 | 2021 | -2.0117 | -2.4853 | -1.5357 | 0 |
| Spain | 5 | 0 | 1990 | 1996 | 0.9433 | 0.5174 | 1.371 | 0.000267 |
| Spain | 5 | 1 | 1996 | 2008 | -1.1625 | -1.3517 | -0.973 | 0 |
| Spain | 5 | 2 | 2008 | 2011 | 1.0104 | -2.1741 | 4.2986 | 0.513699 |
| Spain | 5 | 3 | 2011 | 2014 | -2.1507 | -5.4125 | 1.2235 | 0.191798 |
| Spain | 5 | 4 | 2014 | 2017 | 1.9338 | -1.55 | 5.5409 | 0.258715 |
| Spain | 5 | 5 | 2017 | 2021 | -2.3391 | -3.5241 | -1.1395 | 0.000886 |
| Sweden | 5 | 0 | 1990 | 1995 | 2.1978 | 1.5559 | 2.8438 | 0.000002 |
| Sweden | 5 | 1 | 1995 | 2003 | 2.7448 | 2.3317 | 3.1596 | 0 |
| Sweden | 5 | 2 | 2003 | 2006 | 9.8667 | 6.3502 | 13.4994 | 0.000018 |
| Sweden | 5 | 3 | 2006 | 2009 | -3.7538 | -6.9008 | -0.5003 | 0.026882 |
| Sweden | 5 | 4 | 2009 | 2017 | 2.6388 | 2.1449 | 3.135 | 0 |
| Sweden | 5 | 5 | 2017 | 2021 | -2.3569 | -3.5951 | -1.1028 | 0.001199 |
| United Kingdom | 5 | 0 | 1990 | 1994 | -0.8494 | -1.3955 | -0.3002 | 0.004943 |
| United Kingdom | 5 | 1 | 1994 | 2000 | 0.7807 | 0.387 | 1.1759 | 0.000721 |
| United Kingdom | 5 | 2 | 2000 | 2004 | -0.804 | -1.7341 | 0.1348 | 0.087728 |
| United Kingdom | 5 | 3 | 2004 | 2011 | -0.0744 | -0.4266 | 0.279 | 0.65951 |
| United Kingdom | 5 | 4 | 2011 | 2018 | 2.3375 | 1.9545 | 2.7219 | 0 |
| United Kingdom | 5 | 5 | 2018 | 2021 | -2.2995 | -3.4699 | -1.1149 | 0.000919 |
| United States of America | 5 | 0 | 1990 | 1996 | 1.655 | 1.4586 | 1.8519 | 0 |
| United States of America | 5 | 1 | 1996 | 2000 | 2.4625 | 1.8722 | 3.0564 | 0 |
| United States of America | 5 | 2 | 2000 | 2006 | 0.1628 | -0.0991 | 0.4254 | 0.20516 |
| United States of America | 5 | 3 | 2006 | 2011 | 0.8591 | 0.4543 | 1.2654 | 0.000395 |
| United States of America | 5 | 4 | 2011 | 2017 | 1.423 | 1.1257 | 1.7211 | 0 |
| United States of America | 5 | 5 | 2017 | 2021 | -0.1149 | -0.5389 | 0.3109 | 0.572984 |

| location | Joinpoint Model | AAPC Index | Start Obs | End Obs | AAPC | AAPC C.I. Low | AAPC C.I. High | P-Value |
| --- | --- | --- | --- | --- | --- | --- | --- | --- |
| Australia | 5 | Full Range | 1990 | 2021 | -0.1668 | -0.5224 | 0.1901 | 0.359122 |
| Austria | 5 | Full Range | 1990 | 2021 | 0.6318 | 0.2421 | 1.023 | 0.001466 |
| Belgium | 5 | Full Range | 1990 | 2021 | -0.2521 | -0.9407 | 0.4412 | 0.475062 |
| Canada | 5 | Full Range | 1990 | 2021 | -0.3371 | -0.487 | -0.1871 | 0.000011 |
| Denmark | 5 | Full Range | 1990 | 2021 | 0.5134 | -0.0728 | 1.1031 | 0.086199 |
| Finland | 5 | Full Range | 1990 | 2021 | -1.93 | -2.3895 | -1.4683 | 0 |
| France | 5 | Full Range | 1990 | 2021 | -0.6546 | -0.8424 | -0.4665 | 0 |
| Germany | 5 | Full Range | 1990 | 2021 | 0.6263 | 0.2594 | 0.9947 | 0.000809 |
| Greece | 5 | Full Range | 1990 | 2021 | -0.2813 | -0.7155 | 0.1547 | 0.205712 |
| Ireland | 5 | Full Range | 1990 | 2021 | -0.3317 | -1.2544 | 0.5997 | 0.483877 |
| Italy | 5 | Full Range | 1990 | 2021 | 0.11 | -0.3187 | 0.5406 | 0.615484 |
| Luxembourg | 5 | Full Range | 1990 | 2021 | -0.1192 | -0.4673 | 0.2302 | 0.503286 |
| Netherlands | 5 | Full Range | 1990 | 2021 | -0.6059 | -0.8631 | -0.348 | 0.000004 |
| Norway | 5 | Full Range | 1990 | 2021 | -0.2465 | -1.897 | 1.4318 | 0.771865 |
| Portugal | 5 | Full Range | 1990 | 2021 | -1.2074 | -1.5609 | -0.8527 | 0 |
| Spain | 5 | Full Range | 1990 | 2021 | -0.5028 | -1.0457 | 0.043 | 0.070946 |
| Sweden | 5 | Full Range | 1990 | 2021 | 1.9734 | 1.4906 | 2.4585 | 0 |
| United Kingdom | 5 | Full Range | 1990 | 2021 | 0.2163 | 0.0049 | 0.4281 | 0.044869 |
| United States of America | 5 | Full Range | 1990 | 2021 | 1.0653 | 0.9341 | 1.1968 | 0 |

Table S7. Joinpoint analysis for Atrial fibrillation/flutter disease mortality incidence index (MII) in EU15+ countries for years 1990–2021 in males.

| location_name | Model | Segment | Segment Start | Segment End | APC | APC 95% LCL | APC 95% UCL | P-Value |
| --- | --- | --- | --- | --- | --- | --- | --- | --- |
| Australia | 5 | 0 | 1990 | 2000 | 0.347 | 0.106 | 0.589 | 0.008 |
| Australia | 5 | 1 | 2000 | 2004 | 3.028 | 1.534 | 4.544 | 0.001 |
| Australia | 5 | 2 | 2004 | 2012 | -1.558 | -1.963 | -1.152 | 0 |
| Australia | 5 | 3 | 2012 | 2015 | 0.05 | -3.1 | 3.304 | 0.974 |
| Australia | 5 | 4 | 2015 | 2018 | -3.638 | -6.953 | -0.206 | 0.039 |
| Australia | 5 | 5 | 2018 | 2021 | 1.189 | -1.593 | 4.05 | 0.38 |
| Austria | 5 | 0 | 1990 | 1995 | -0.036 | -1.423 | 1.37 | 0.957 |
| Austria | 5 | 1 | 1995 | 1999 | 2.775 | -0.216 | 5.856 | 0.067 |
| Austria | 5 | 2 | 1999 | 2006 | -2.058 | -2.976 | -1.13 | 0 |
| Austria | 5 | 3 | 2006 | 2010 | -0.798 | -3.061 | 1.518 | 0.471 |
| Austria | 5 | 4 | 2010 | 2014 | -4.099 | -6.353 | -1.792 | 0.002 |
| Austria | 5 | 5 | 2014 | 2021 | -1.301 | -1.96 | -0.639 | 0.001 |
| Belgium | 5 | 0 | 1990 | 1996 | -0.57 | -1.276 | 0.14 | 0.107 |
| Belgium | 5 | 1 | 1996 | 1999 | 7.13 | 2.777 | 11.668 | 0.003 |
| Belgium | 5 | 2 | 1999 | 2007 | 0.899 | 0.379 | 1.422 | 0.002 |
| Belgium | 5 | 3 | 2007 | 2010 | -0.635 | -4.264 | 3.131 | 0.72 |
| Belgium | 5 | 4 | 2010 | 2016 | 1.106 | 0.25 | 1.969 | 0.015 |
| Belgium | 5 | 5 | 2016 | 2021 | -3.539 | -4.394 | -2.676 | 0 |
| Canada | 5 | 0 | 1990 | 1999 | 0.77 | 0.69 | 0.85 | 0 |
| Canada | 5 | 1 | 1999 | 2005 | 0.289 | 0.087 | 0.49 | 0.008 |
| Canada | 5 | 2 | 2005 | 2009 | -0.104 | -0.576 | 0.371 | 0.648 |
| Canada | 5 | 3 | 2009 | 2014 | -0.504 | -0.824 | -0.183 | 0.004 |
| Canada | 5 | 4 | 2014 | 2019 | -1.614 | -1.947 | -1.28 | 0 |
| Canada | 5 | 5 | 2019 | 2021 | 1.618 | 0.513 | 2.734 | 0.007 |
| Denmark | 5 | 0 | 1990 | 1992 | -4.738 | -10.754 | 1.684 | 0.134 |
| Denmark | 5 | 1 | 1992 | 1996 | 1.595 | -0.589 | 3.826 | 0.142 |
| Denmark | 5 | 2 | 1996 | 1999 | 5.671 | 1.276 | 10.256 | 0.014 |
| Denmark | 5 | 3 | 1999 | 2011 | 2.62 | 2.281 | 2.96 | 0 |
| Denmark | 5 | 4 | 2011 | 2016 | -0.297 | -1.994 | 1.429 | 0.717 |
| Denmark | 5 | 5 | 2016 | 2021 | -2.225 | -3.534 | -0.898 | 0.003 |
| Finland | 5 | 0 | 1990 | 1995 | -1.145 | -1.932 | -0.351 | 0.008 |
| Finland | 5 | 1 | 1995 | 1999 | 5.227 | 3.472 | 7.011 | 0 |
| Finland | 5 | 2 | 1999 | 2006 | 0.656 | 0.108 | 1.207 | 0.022 |
| Finland | 5 | 3 | 2006 | 2009 | -5.678 | -8.74 | -2.514 | 0.002 |
| Finland | 5 | 4 | 2009 | 2018 | 0.159 | -0.208 | 0.527 | 0.371 |
| Finland | 5 | 5 | 2018 | 2021 | -1.687 | -3.289 | -0.059 | 0.043 |
| France | 5 | 0 | 1990 | 1994 | 0.15 | -0.286 | 0.588 | 0.475 |
| France | 5 | 1 | 1994 | 1997 | 1.056 | -0.355 | 2.486 | 0.132 |
| France | 5 | 2 | 1997 | 2003 | 0.192 | -0.124 | 0.51 | 0.215 |
| France | 5 | 3 | 2003 | 2008 | -0.614 | -1.062 | -0.163 | 0.011 |
| France | 5 | 4 | 2008 | 2018 | 0.28 | 0.145 | 0.415 | 0 |
| France | 5 | 5 | 2018 | 2021 | -2.73 | -3.472 | -1.982 | 0 |
| Germany | 5 | 0 | 1990 | 1995 | -0.991 | -2.381 | 0.419 | 0.154 |
| Germany | 5 | 1 | 1995 | 2000 | -5.017 | -6.189 | -3.829 | 0 |
| Germany | 5 | 2 | 2000 | 2004 | 8.584 | 6.879 | 10.317 | 0 |
| Germany | 5 | 3 | 2004 | 2007 | 2.349 | -0.997 | 5.808 | 0.157 |
| Germany | 5 | 4 | 2007 | 2017 | 1.069 | 0.76 | 1.378 | 0 |
| Germany | 5 | 5 | 2017 | 2021 | -2.045 | -3.265 | -0.809 | 0.003 |
| Greece | 5 | 0 | 1990 | 1996 | 0.404 | -0.024 | 0.834 | 0.063 |
| Greece | 5 | 1 | 1996 | 1999 | -0.67 | -2.786 | 1.492 | 0.516 |
| Greece | 5 | 2 | 1999 | 2004 | -2.943 | -3.636 | -2.245 | 0 |
| Greece | 5 | 3 | 2004 | 2010 | 0.674 | 0.06 | 1.293 | 0.033 |
| Greece | 5 | 4 | 2010 | 2014 | 2.442 | 0.862 | 4.046 | 0.005 |
| Greece | 5 | 5 | 2014 | 2021 | 0.623 | 0.111 | 1.137 | 0.02 |
| Ireland | 5 | 0 | 1990 | 1999 | 2.171 | 1.188 | 3.164 | 0 |
| Ireland | 5 | 1 | 1999 | 2002 | -2.295 | -9.017 | 4.924 | 0.498 |
| Ireland | 5 | 2 | 2002 | 2005 | 5.089 | -2.425 | 13.182 | 0.174 |
| Ireland | 5 | 3 | 2005 | 2008 | -6.829 | -13.708 | 0.598 | 0.068 |
| Ireland | 5 | 4 | 2008 | 2014 | 3.617 | 1.848 | 5.417 | 0.001 |
| Ireland | 5 | 5 | 2014 | 2021 | -2.684 | -3.891 | -1.461 | 0 |
| Italy | 5 | 0 | 1990 | 1995 | -0.056 | -0.934 | 0.83 | 0.894 |
| Italy | 5 | 1 | 1995 | 2004 | 3.461 | 3.022 | 3.902 | 0 |
| Italy | 5 | 2 | 2004 | 2007 | 1.118 | -2.786 | 5.178 | 0.556 |
| Italy | 5 | 3 | 2007 | 2015 | 2.893 | 2.331 | 3.459 | 0 |
| Italy | 5 | 4 | 2015 | 2019 | 0.666 | -1.444 | 2.821 | 0.514 |
| Italy | 5 | 5 | 2019 | 2021 | -11.15 | -15.007 | -7.119 | 0 |
| Luxembourg | 5 | 0 | 1990 | 1999 | -0.675 | -0.952 | -0.397 | 0 |
| Luxembourg | 5 | 1 | 1999 | 2003 | 2.625 | 1.282 | 3.985 | 0.001 |
| Luxembourg | 5 | 2 | 2003 | 2010 | 0.93 | 0.47 | 1.393 | 0.001 |
| Luxembourg | 5 | 3 | 2010 | 2013 | -0.731 | -3.399 | 2.011 | 0.575 |
| Luxembourg | 5 | 4 | 2013 | 2016 | 1.623 | -1.501 | 4.846 | 0.289 |
| Luxembourg | 5 | 5 | 2016 | 2021 | -3.142 | -3.988 | -2.289 | 0 |
| Netherlands | 5 | 0 | 1990 | 1996 | -0.156 | -0.53 | 0.219 | 0.388 |
| Netherlands | 5 | 1 | 1996 | 2000 | -2.309 | -3.373 | -1.233 | 0 |
| Netherlands | 5 | 2 | 2000 | 2006 | 1.092 | 0.529 | 1.658 | 0.001 |
| Netherlands | 5 | 3 | 2006 | 2009 | -0.412 | -3.061 | 2.309 | 0.749 |
| Netherlands | 5 | 4 | 2009 | 2016 | 0.893 | 0.403 | 1.386 | 0.001 |
| Netherlands | 5 | 5 | 2016 | 2021 | 0.031 | -0.606 | 0.672 | 0.919 |
| Norway | 5 | 0 | 1990 | 1993 | 9.389 | 3.017 | 16.154 | 0.006 |
| Norway | 5 | 1 | 1993 | 1997 | -4.571 | -10.131 | 1.332 | 0.117 |
| Norway | 5 | 2 | 1997 | 2001 | 3.271 | -2.768 | 9.684 | 0.273 |
| Norway | 5 | 3 | 2001 | 2004 | -4.308 | -15.703 | 8.626 | 0.471 |
| Norway | 5 | 4 | 2004 | 2012 | 2.105 | 0.375 | 3.864 | 0.02 |
| Norway | 5 | 5 | 2012 | 2021 | -3.376 | -4.541 | -2.197 | 0 |
| Portugal | 5 | 0 | 1990 | 1996 | 0.348 | -0.564 | 1.27 | 0.43 |
| Portugal | 5 | 1 | 1996 | 2000 | 3.764 | 1.667 | 5.905 | 0.002 |
| Portugal | 5 | 2 | 2000 | 2003 | -1.238 | -4.544 | 2.183 | 0.448 |
| Portugal | 5 | 3 | 2003 | 2011 | -3.105 | -3.56 | -2.647 | 0 |
| Portugal | 5 | 4 | 2011 | 2014 | 2.459 | -1.193 | 6.246 | 0.174 |
| Portugal | 5 | 5 | 2014 | 2021 | -0.26 | -0.803 | 0.285 | 0.324 |
| Spain | 5 | 0 | 1990 | 1995 | 1.872 | 0.961 | 2.791 | 0.001 |
| Spain | 5 | 1 | 1995 | 2007 | -1.976 | -2.171 | -1.781 | 0 |
| Spain | 5 | 2 | 2007 | 2011 | 2.451 | 1.031 | 3.892 | 0.002 |
| Spain | 5 | 3 | 2011 | 2014 | 0.027 | -3.018 | 3.167 | 0.986 |
| Spain | 5 | 4 | 2014 | 2018 | 2.482 | 0.726 | 4.269 | 0.009 |
| Spain | 5 | 5 | 2018 | 2021 | -4.087 | -5.669 | -2.479 | 0 |
| Sweden | 5 | 0 | 1990 | 2003 | 0.683 | 0.193 | 1.177 | 0.01 |
| Sweden | 5 | 1 | 2003 | 2006 | 15 | 5.604 | 25.231 | 0.003 |
| Sweden | 5 | 2 | 2006 | 2009 | -6.368 | -14.315 | 2.316 | 0.135 |
| Sweden | 5 | 3 | 2009 | 2016 | 2.634 | 1.034 | 4.26 | 0.003 |
| Sweden | 5 | 4 | 2016 | 2019 | -1.043 | -9.972 | 8.771 | 0.816 |
| Sweden | 5 | 5 | 2019 | 2021 | -8.251 | -19.682 | 4.806 | 0.188 |
| United Kingdom | 5 | 0 | 1990 | 1993 | -1.726 | -2.754 | -0.688 | 0.003 |
| United Kingdom | 5 | 1 | 1993 | 2005 | 0.299 | 0.174 | 0.424 | 0 |
| United Kingdom | 5 | 2 | 2005 | 2008 | 1.065 | -0.698 | 2.859 | 0.219 |
| United Kingdom | 5 | 3 | 2008 | 2011 | -0.086 | -1.872 | 1.732 | 0.92 |
| United Kingdom | 5 | 4 | 2011 | 2018 | 1.226 | 0.898 | 1.556 | 0 |
| United Kingdom | 5 | 5 | 2018 | 2021 | -2.337 | -3.365 | -1.299 | 0 |
| United States of America | 5 | 0 | 1990 | 1993 | 4.543 | 3.184 | 5.92 | 0 |
| United States of America | 5 | 1 | 1993 | 2000 | 1.375 | 0.936 | 1.816 | 0 |
| United States of America | 5 | 2 | 2000 | 2005 | -2.848 | -3.383 | -2.311 | 0 |
| United States of America | 5 | 3 | 2005 | 2014 | 0.506 | 0.373 | 0.64 | 0 |
| United States of America | 5 | 4 | 2014 | 2018 | 1.064 | 0.445 | 1.687 | 0.002 |
| United States of America | 5 | 5 | 2018 | 2021 | -1.537 | -2.184 | -0.886 | 0 |

| location | Joinpoint Model | AAPC Index | Start Obs | End Obs | AAPC | AAPC C.I. Low | AAPC C.I. High | P-Value |
| --- | --- | --- | --- | --- | --- | --- | --- | --- |
| Australia | 5 | Full Range | 1990 | 2021 | -0.148 | -0.679 | 0.386 | 0.587 |
| Austria | 5 | Full Range | 1990 | 2021 | -1.056 | -1.662 | -0.446 | 0.001 |
| Belgium | 5 | Full Range | 1990 | 2021 | 0.358 | -0.206 | 0.924 | 0.214 |
| Canada | 5 | Full Range | 1990 | 2021 | 0.025 | -0.093 | 0.143 | 0.682 |
| Denmark | 5 | Full Range | 1990 | 2021 | 1.02 | 0.324 | 1.721 | 0.004 |
| Finland | 5 | Full Range | 1990 | 2021 | -0.065 | -0.493 | 0.365 | 0.766 |
| France | 5 | Full Range | 1990 | 2021 | -0.119 | -0.298 | 0.061 | 0.194 |
| Germany | 5 | Full Range | 1990 | 2021 | 0.374 | -0.108 | 0.857 | 0.128 |
| Greece | 5 | Full Range | 1990 | 2021 | 0.113 | -0.22 | 0.447 | 0.507 |
| Ireland | 5 | Full Range | 1990 | 2021 | 0.269 | -0.965 | 1.517 | 0.671 |
| Italy | 5 | Full Range | 1990 | 2021 | 1.152 | 0.597 | 1.71 | 0 |
| Luxembourg | 5 | Full Range | 1990 | 2021 | -0.083 | -0.52 | 0.356 | 0.71 |
| Netherlands | 5 | Full Range | 1990 | 2021 | 0.044 | -0.284 | 0.374 | 0.791 |
| Norway | 5 | Full Range | 1990 | 2021 | -0.206 | -1.875 | 1.492 | 0.811 |
| Portugal | 5 | Full Range | 1990 | 2021 | -0.214 | -0.765 | 0.34 | 0.448 |
| Spain | 5 | Full Range | 1990 | 2021 | -0.246 | -0.679 | 0.19 | 0.268 |
| Sweden | 5 | Full Range | 1990 | 2021 | 0.936 | -0.701 | 2.599 | 0.264 |
| United Kingdom | 5 | Full Range | 1990 | 2021 | 0.087 | -0.185 | 0.361 | 0.53 |
| United States of America | 5 | Full Range | 1990 | 2021 | 0.406 | 0.21 | 0.603 | 0 |

Table S8. Joinpoint analysis for Atrial fibrillation/flutter disease mortality incidence index (MII) in EU15+ countries for years 1990–2021 in females.

| location_name | Model | Segment | Segment Start | Segment End | APC | APC 95% LCL | APC 95% UCL | P-Value |
| --- | --- | --- | --- | --- | --- | --- | --- | --- |
| Australia | 5 | 0 | 1990 | 2001 | -0.095 | -0.262 | 0.073 | 0.247 |
| Australia | 5 | 1 | 2001 | 2004 | 2.489 | -0.063 | 5.107 | 0.055 |
| Australia | 5 | 2 | 2004 | 2008 | -0.057 | -1.368 | 1.271 | 0.928 |
| Australia | 5 | 3 | 2008 | 2012 | -0.672 | -1.989 | 0.662 | 0.299 |
| Australia | 5 | 4 | 2012 | 2015 | -0.129 | -2.93 | 2.753 | 0.924 |
| Australia | 5 | 5 | 2015 | 2021 | -3.104 | -3.759 | -2.445 | 0 |
| Austria | 5 | 0 | 1990 | 1994 | -0.489 | -1.98 | 1.026 | 0.5 |
| Austria | 5 | 1 | 1994 | 1999 | 1.943 | 0.46 | 3.448 | 0.014 |
| Austria | 5 | 2 | 1999 | 2006 | -2.303 | -3.022 | -1.579 | 0 |
| Austria | 5 | 3 | 2006 | 2010 | 0.317 | -1.661 | 2.336 | 0.739 |
| Austria | 5 | 4 | 2010 | 2015 | -4.784 | -6.011 | -3.542 | 0 |
| Austria | 5 | 5 | 2015 | 2021 | -0.675 | -1.393 | 0.049 | 0.065 |
| Belgium | 5 | 0 | 1990 | 1996 | 0.198 | -0.643 | 1.046 | 0.624 |
| Belgium | 5 | 1 | 1996 | 1999 | 5.388 | 0.171 | 10.877 | 0.044 |
| Belgium | 5 | 2 | 1999 | 2006 | 2.064 | 1.23 | 2.904 | 0 |
| Belgium | 5 | 3 | 2006 | 2015 | 0.412 | -0.112 | 0.938 | 0.115 |
| Belgium | 5 | 4 | 2015 | 2019 | -2.908 | -5.286 | -0.471 | 0.023 |
| Belgium | 5 | 5 | 2019 | 2021 | -5.641 | -10.276 | -0.766 | 0.027 |
| Canada | 5 | 0 | 1990 | 1994 | 5.081 | 4.428 | 5.737 | 0 |
| Canada | 5 | 1 | 1994 | 2003 | 0.002 | -0.216 | 0.221 | 0.983 |
| Canada | 5 | 2 | 2003 | 2009 | 0.92 | 0.437 | 1.405 | 0.001 |
| Canada | 5 | 3 | 2009 | 2015 | -1.877 | -2.399 | -1.352 | 0 |
| Canada | 5 | 4 | 2015 | 2019 | 2.559 | 1.345 | 3.789 | 0 |
| Canada | 5 | 5 | 2019 | 2021 | -2.673 | -5.016 | -0.272 | 0.032 |
| Denmark | 5 | 0 | 1990 | 1992 | -2.734 | -7.792 | 2.602 | 0.286 |
| Denmark | 5 | 1 | 1992 | 1997 | 2.681 | 1.32 | 4.061 | 0.001 |
| Denmark | 5 | 2 | 1997 | 2001 | 4.025 | 1.822 | 6.275 | 0.001 |
| Denmark | 5 | 3 | 2001 | 2011 | 1.598 | 1.169 | 2.029 | 0 |
| Denmark | 5 | 4 | 2011 | 2014 | -5.496 | -10.124 | -0.629 | 0.03 |
| Denmark | 5 | 5 | 2014 | 2021 | -0.826 | -1.557 | -0.089 | 0.031 |
| Finland | 5 | 0 | 1990 | 1995 | -0.736 | -1.787 | 0.326 | 0.16 |
| Finland | 5 | 1 | 1995 | 2000 | 4.094 | 2.757 | 5.448 | 0 |
| Finland | 5 | 2 | 2000 | 2007 | -1.316 | -2.014 | -0.613 | 0.001 |
| Finland | 5 | 3 | 2007 | 2010 | -7.524 | -11.437 | -3.439 | 0.002 |
| Finland | 5 | 4 | 2010 | 2014 | 0.752 | -1.474 | 3.028 | 0.486 |
| Finland | 5 | 5 | 2014 | 2021 | -2.158 | -2.777 | -1.535 | 0 |
| France | 5 | 0 | 1990 | 2000 | 0.185 | 0.086 | 0.284 | 0.001 |
| France | 5 | 1 | 2000 | 2003 | 0.998 | -0.302 | 2.316 | 0.123 |
| France | 5 | 2 | 2003 | 2006 | -0.023 | -1.316 | 1.287 | 0.97 |
| France | 5 | 3 | 2006 | 2013 | 0.287 | 0.061 | 0.515 | 0.016 |
| France | 5 | 4 | 2013 | 2016 | 1.167 | -0.23 | 2.582 | 0.096 |
| France | 5 | 5 | 2016 | 2021 | -2.434 | -2.742 | -2.125 | 0 |
| Germany | 5 | 0 | 1990 | 1996 | 1.481 | 0.669 | 2.3 | 0.001 |
| Germany | 5 | 1 | 1996 | 2000 | -2.294 | -3.801 | -0.763 | 0.006 |
| Germany | 5 | 2 | 2000 | 2003 | 7.776 | 4.906 | 10.723 | 0 |
| Germany | 5 | 3 | 2003 | 2007 | 2.95 | 1.557 | 4.362 | 0 |
| Germany | 5 | 4 | 2007 | 2018 | 0.387 | 0.159 | 0.616 | 0.003 |
| Germany | 5 | 5 | 2018 | 2021 | -2.348 | -4.043 | -0.623 | 0.011 |
| Greece | 5 | 0 | 1990 | 1996 | -1.594 | -2.063 | -1.123 | 0 |
| Greece | 5 | 1 | 1996 | 1999 | 0.898 | -1.739 | 3.605 | 0.483 |
| Greece | 5 | 2 | 1999 | 2007 | -0.315 | -0.665 | 0.037 | 0.076 |
| Greece | 5 | 3 | 2007 | 2010 | -2.042 | -4.664 | 0.651 | 0.126 |
| Greece | 5 | 4 | 2010 | 2014 | 6.512 | 4.921 | 8.127 | 0 |
| Greece | 5 | 5 | 2014 | 2021 | -1.1 | -1.568 | -0.63 | 0 |
| Ireland | 5 | 0 | 1990 | 1996 | 1.101 | -0.094 | 2.311 | 0.068 |
| Ireland | 5 | 1 | 1996 | 1999 | 8.829 | 1.872 | 16.261 | 0.016 |
| Ireland | 5 | 2 | 1999 | 2010 | -0.323 | -0.818 | 0.175 | 0.186 |
| Ireland | 5 | 3 | 2010 | 2014 | 6.326 | 2.86 | 9.908 | 0.001 |
| Ireland | 5 | 4 | 2014 | 2019 | -1.264 | -3.401 | 0.92 | 0.234 |
| Ireland | 5 | 5 | 2019 | 2021 | -16.067 | -22.498 | -9.102 | 0 |
| Italy | 5 | 0 | 1990 | 1998 | -0.16 | -0.688 | 0.372 | 0.531 |
| Italy | 5 | 1 | 1998 | 2001 | -1.608 | -6.158 | 3.164 | 0.477 |
| Italy | 5 | 2 | 2001 | 2006 | 1.282 | -0.269 | 2.857 | 0.099 |
| Italy | 5 | 3 | 2006 | 2009 | 5.947 | 0.727 | 11.438 | 0.028 |
| Italy | 5 | 4 | 2009 | 2018 | 1.364 | 0.792 | 1.94 | 0 |
| Italy | 5 | 5 | 2018 | 2021 | -4.2 | -6.773 | -1.556 | 0.004 |
| Luxembourg | 5 | 0 | 1990 | 1993 | 3.233 | 1.178 | 5.328 | 0.004 |
| Luxembourg | 5 | 1 | 1993 | 2000 | -0.104 | -0.637 | 0.432 | 0.686 |
| Luxembourg | 5 | 2 | 2000 | 2004 | 3.613 | 2.112 | 5.136 | 0 |
| Luxembourg | 5 | 3 | 2004 | 2011 | -1.23 | -1.738 | -0.719 | 0 |
| Luxembourg | 5 | 4 | 2011 | 2015 | 4.38 | 2.95 | 5.83 | 0 |
| Luxembourg | 5 | 5 | 2015 | 2021 | -3.673 | -4.211 | -3.133 | 0 |
| Netherlands | 5 | 0 | 1990 | 1993 | 0.234 | -0.447 | 0.919 | 0.476 |
| Netherlands | 5 | 1 | 1993 | 1996 | -0.645 | -1.97 | 0.698 | 0.32 |
| Netherlands | 5 | 2 | 1996 | 2000 | -2.8 | -3.464 | -2.131 | 0 |
| Netherlands | 5 | 3 | 2000 | 2005 | 1.191 | 0.682 | 1.702 | 0 |
| Netherlands | 5 | 4 | 2005 | 2017 | 0.552 | 0.419 | 0.686 | 0 |
| Netherlands | 5 | 5 | 2017 | 2021 | -1.318 | -1.958 | -0.675 | 0.001 |
| Norway | 5 | 0 | 1990 | 1994 | 4.046 | 0.293 | 7.94 | 0.036 |
| Norway | 5 | 1 | 1994 | 1997 | -3.726 | -14.582 | 8.509 | 0.509 |
| Norway | 5 | 2 | 1997 | 2001 | 3.563 | -2.401 | 9.892 | 0.228 |
| Norway | 5 | 3 | 2001 | 2004 | -1.94 | -12.84 | 10.322 | 0.728 |
| Norway | 5 | 4 | 2004 | 2013 | 2.33 | 0.973 | 3.705 | 0.002 |
| Norway | 5 | 5 | 2013 | 2021 | -3.072 | -4.397 | -1.727 | 0 |
| Portugal | 5 | 0 | 1990 | 2002 | 0.3 | 0.172 | 0.427 | 0 |
| Portugal | 5 | 1 | 2002 | 2005 | -2.74 | -4.373 | -1.08 | 0.003 |
| Portugal | 5 | 2 | 2005 | 2009 | -5.031 | -5.885 | -4.17 | 0 |
| Portugal | 5 | 3 | 2009 | 2012 | -0.998 | -2.882 | 0.922 | 0.283 |
| Portugal | 5 | 4 | 2012 | 2015 | 3.001 | 0.998 | 5.043 | 0.006 |
| Portugal | 5 | 5 | 2015 | 2021 | -2.025 | -2.391 | -1.657 | 0 |
| Spain | 5 | 0 | 1990 | 1995 | 2.987 | 1.884 | 4.101 | 0 |
| Spain | 5 | 1 | 1995 | 2000 | -3.398 | -4.538 | -2.244 | 0 |
| Spain | 5 | 2 | 2000 | 2005 | 0.503 | -0.715 | 1.737 | 0.394 |
| Spain | 5 | 3 | 2005 | 2008 | -0.855 | -4.434 | 2.858 | 0.626 |
| Spain | 5 | 4 | 2008 | 2018 | 0.983 | 0.591 | 1.378 | 0 |
| Spain | 5 | 5 | 2018 | 2021 | -3.105 | -5.526 | -0.621 | 0.018 |
| Sweden | 5 | 0 | 1990 | 1994 | 0.111 | -2.462 | 2.752 | 0.929 |
| Sweden | 5 | 1 | 1994 | 2003 | 3.858 | 3.049 | 4.673 | 0 |
| Sweden | 5 | 2 | 2003 | 2006 | 7.456 | 0.012 | 15.455 | 0.05 |
| Sweden | 5 | 3 | 2006 | 2009 | -6.131 | -12.603 | 0.821 | 0.079 |
| Sweden | 5 | 4 | 2009 | 2018 | 2.377 | 1.542 | 3.219 | 0 |
| Sweden | 5 | 5 | 2018 | 2021 | -10.749 | -14.678 | -6.639 | 0 |
| United Kingdom | 5 | 0 | 1990 | 1994 | -1.106 | -1.708 | -0.501 | 0.001 |
| United Kingdom | 5 | 1 | 1994 | 2005 | 0.429 | 0.295 | 0.564 | 0 |
| United Kingdom | 5 | 2 | 2005 | 2008 | 2.882 | 1.191 | 4.602 | 0.002 |
| United Kingdom | 5 | 3 | 2008 | 2011 | 0.225 | -1.469 | 1.949 | 0.782 |
| United Kingdom | 5 | 4 | 2011 | 2018 | 1.113 | 0.808 | 1.419 | 0 |
| United Kingdom | 5 | 5 | 2018 | 2021 | -2.826 | -3.784 | -1.859 | 0 |
| United States of America | 5 | 0 | 1990 | 1994 | -1.384 | -2.165 | -0.597 | 0.002 |
| United States of America | 5 | 1 | 1994 | 2000 | 2.023 | 1.455 | 2.595 | 0 |
| United States of America | 5 | 2 | 2000 | 2005 | -1.523 | -2.062 | -0.982 | 0 |
| United States of America | 5 | 3 | 2005 | 2008 | 1.627 | 0.318 | 2.953 | 0.018 |
| United States of America | 5 | 4 | 2008 | 2017 | 1.172 | 1.028 | 1.316 | 0 |
| United States of America | 5 | 5 | 2017 | 2021 | -1.291 | -1.717 | -0.863 | 0 |

| location_name | Joinpoint Model | AAPC Index | Start Obs | End Obs | AAPC | AAPC C.I. Low | AAPC C.I. High | P-Value |
| --- | --- | --- | --- | --- | --- | --- | --- | --- |
| Australia | 5 | Full Range | 1990 | 2021 | -0.512 | -0.935 | -0.087 | 0.018 |
| Austria | 5 | Full Range | 1990 | 2021 | -1.153 | -1.608 | -0.697 | 0 |
| Belgium | 5 | Full Range | 1990 | 2021 | 0.372 | -0.3 | 1.048 | 0.278 |
| Canada | 5 | Full Range | 1990 | 2021 | 0.604 | 0.345 | 0.863 | 0 |
| Denmark | 5 | Full Range | 1990 | 2021 | 0.536 | -0.131 | 1.207 | 0.116 |
| Finland | 5 | Full Range | 1990 | 2021 | -0.92 | -1.478 | -0.359 | 0.001 |
| France | 5 | Full Range | 1990 | 2021 | -0.067 | -0.284 | 0.151 | 0.547 |
| Germany | 5 | Full Range | 1990 | 2021 | 0.997 | 0.584 | 1.412 | 0 |
| Greece | 5 | Full Range | 1990 | 2021 | 0.059 | -0.353 | 0.472 | 0.78 |
| Ireland | 5 | Full Range | 1990 | 2021 | 0.373 | -0.575 | 1.33 | 0.442 |
| Italy | 5 | Full Range | 1990 | 2021 | 0.546 | -0.183 | 1.281 | 0.142 |
| Luxembourg | 5 | Full Range | 1990 | 2021 | 0.292 | -0.058 | 0.644 | 0.102 |
| Netherlands | 5 | Full Range | 1990 | 2021 | -0.173 | -0.369 | 0.022 | 0.083 |
| Norway | 5 | Full Range | 1990 | 2021 | 0.27 | -1.494 | 2.066 | 0.766 |
| Portugal | 5 | Full Range | 1990 | 2021 | -1.021 | -1.334 | -0.707 | 0 |
| Spain | 5 | Full Range | 1990 | 2021 | -0.075 | -0.583 | 0.437 | 0.775 |
| Sweden | 5 | Full Range | 1990 | 2021 | 0.781 | -0.298 | 1.873 | 0.157 |
| United Kingdom | 5 | Full Range | 1990 | 2021 | 0.278 | 0.025 | 0.531 | 0.031 |
| United States of America | 5 | Full Range | 1990 | 2021 | 0.288 | 0.08 | 0.495 | 0.007 |
