# Supplementary material for: Temporal Trend Analysis of Atrial Fibrillation/Flutter Disease Burden in High-Income Countries Between 1990 and 2021
Source: Rev Cardiovasc Med. 2025 Jul 25;26(7):36427. doi: 10.31083/RCM36427 (PMC12326443; doi:10.31083/RCM36427)
Supplement: Supplementary file 1 [file 2153-8174-26-7-36427-s1.zip › Supplementary Material 3.docx]

Table S9. The Local drifts for EU15+ in female and male both. (%)

| Country | Age | Female_localdrifts | Male_localdrifts | Both_localdrifts |
| --- | --- | --- | --- | --- |
| Australia | 30-34 | -0.58(-1.58 to 0.44) | -0.76(-1.37 to -0.15) | -0.73(-1.25 to -0.21) |
| Australia | 35-39 | -0.19(-0.76 to 0.38) | -0.51(-0.85 to -0.16) | -0.43(-0.72 to -0.14) |
| Australia | 40-44 | 0.13(-0.29 to 0.55) | -0.20(-0.46 to 0.05) | -0.14(-0.35 to 0.08) |
| Australia | 45-49 | 0.30(-0.04 to 0.63) | 0.00(-0.20 to 0.21) | 0.03(-0.14 to 0.21) |
| Australia | 50-54 | 0.34(0.07 to 0.61) | 0.09(-0.09 to 0.27) | 0.11(-0.04 to 0.26) |
| Australia | 55-59 | 0.45(0.23 to 0.67) | 0.10(-0.05 to 0.26) | 0.18(0.05 to 0.31) |
| Australia | 60-64 | 0.61(0.42 to 0.79) | 0.11(-0.03 to 0.25) | 0.27(0.16 to 0.38) |
| Australia | 65-69 | 0.63(0.47 to 0.79) | 0.11(-0.01 to 0.24) | 0.33(0.23 to 0.43) |
| Australia | 70-74 | 0.50(0.35 to 0.65) | 0.07(-0.06 to 0.20) | 0.30(0.20 to 0.39) |
| Australia | 75-79 | 0.31(0.16 to 0.46) | -0.05(-0.21 to 0.11) | 0.16(0.05 to 0.27) |
| Australia | 80-84 | 0.03(-0.14 to 0.21) | -0.26(-0.48 to -0.03) | -0.10(-0.23 to 0.04) |
| Australia | 85-89 | -0.38(-0.62 to -0.14) | -0.56(-0.96 to -0.16) | -0.52(-0.72 to -0.31) |
| Australia | 90-94 | -0.85(-1.25 to -0.45) | -0.90(-1.73 to -0.05) | -1.00(-1.35 to -0.64) |
| Australia | 95-99 | -1.29(-2.14 to -0.43) | -1.14(-3.38 to 1.16) | -1.43(-2.22 to -0.64) |
| Austria | 30-34 | 1.53(-1.19 to 4.32) | 1.56(0.50 to 2.63) | 1.59(0.60 to 2.58) |
| Austria | 35-39 | 1.33(-0.17 to 2.84) | 1.65(1.06 to 2.26) | 1.61(1.05 to 2.17) |
| Austria | 40-44 | 1.14(0.11 to 2.17) | 1.79(1.34 to 2.24) | 1.67(1.26 to 2.07) |
| Austria | 45-49 | 1.17(0.43 to 1.91) | 1.93(1.57 to 2.29) | 1.78(1.46 to 2.10) |
| Austria | 50-54 | 1.44(0.92 to 1.97) | 2.06(1.77 to 2.35) | 1.96(1.70 to 2.21) |
| Austria | 55-59 | 1.87(1.48 to 2.26) | 2.18(1.93 to 2.43) | 2.16(1.95 to 2.37) |
| Austria | 60-64 | 2.40(2.11 to 2.68) | 2.34(2.13 to 2.56) | 2.44(2.27 to 2.61) |
| Austria | 65-69 | 2.65(2.43 to 2.87) | 2.43(2.24 to 2.62) | 2.62(2.48 to 2.76) |
| Austria | 70-74 | 2.58(2.39 to 2.76) | 2.42(2.24 to 2.61) | 2.62(2.49 to 2.75) |
| Austria | 75-79 | 2.26(2.07 to 2.45) | 2.32(2.09 to 2.56) | 2.39(2.24 to 2.53) |
| Austria | 80-84 | 1.67(1.45 to 1.89) | 2.01(1.69 to 2.33) | 1.84(1.66 to 2.02) |
| Austria | 85-89 | 0.70(0.38 to 1.01) | 1.18(0.58 to 1.78) | 0.82(0.54 to 1.10) |
| Austria | 90-94 | -0.38(-0.96 to 0.21) | -0.15(-1.58 to 1.29) | -0.38(-0.91 to 0.16) |
| Austria | 95-99 | -1.19(-2.67 to 0.31) | -1.21(-5.37 to 3.14) | -1.28(-2.67 to 0.14) |
| Belgium | 30-34 | 1.73(-0.15 to 3.64) | 1.81(0.65 to 2.99) | 1.76(0.54 to 2.99) |
| Belgium | 35-39 | 1.84(0.78 to 2.92) | 1.86(1.19 to 2.54) | 1.84(1.14 to 2.55) |
| Belgium | 40-44 | 1.66(0.87 to 2.46) | 1.48(0.94 to 2.02) | 1.53(0.98 to 2.09) |
| Belgium | 45-49 | 1.22(0.60 to 1.84) | 0.75(0.30 to 1.22) | 0.91(0.44 to 1.37) |
| Belgium | 50-54 | 0.60(0.11 to 1.09) | -0.08(-0.47 to 0.32) | 0.15(-0.23 to 0.54) |
| Belgium | 55-59 | 0.02(-0.36 to 0.40) | -0.69(-1.02 to -0.35) | -0.42(-0.74 to -0.10) |
| Belgium | 60-64 | -0.43(-0.72 to -0.14) | -0.97(-1.25 to -0.68) | -0.73(-0.99 to -0.47) |
| Belgium | 65-69 | -0.79(-1.02 to -0.56) | -1.06(-1.31 to -0.82) | -0.91(-1.13 to -0.69) |
| Belgium | 70-74 | -1.18(-1.37 to -0.98) | -1.12(-1.36 to -0.88) | -1.13(-1.33 to -0.94) |
| Belgium | 75-79 | -1.54(-1.72 to -1.34) | -1.17(-1.46 to -0.87) | -1.40(-1.61 to -1.18) |
| Belgium | 80-84 | -1.76(-1.96 to -1.55) | -1.21(-1.60 to -0.81) | -1.61(-1.87 to -1.36) |
| Belgium | 85-89 | -1.83(-2.13 to -1.53) | -1.28(-2.00 to -0.55) | -1.77(-2.15 to -1.38) |
| Belgium | 90-94 | -1.81(-2.35 to -1.27) | -1.42(-3.10 to 0.28) | -1.86(-2.59 to -1.12) |
| Belgium | 95-99 | -1.82(-3.13 to -0.48) | -1.58(-6.22 to 3.30) | -1.90(-3.73 to -0.04) |
| Canada | 30-34 | -0.58(-2.12 to 0.98) | -0.17(-0.99 to 0.66) | -0.29(-1.30 to 0.72) |
| Canada | 35-39 | -0.43(-1.26 to 0.40) | 0.08(-0.36 to 0.54) | -0.08(-0.63 to 0.47) |
| Canada | 40-44 | -0.29(-0.86 to 0.28) | 0.42(0.11 to 0.74) | 0.20(-0.18 to 0.59) |
| Canada | 45-49 | -0.37(-0.79 to 0.06) | 0.58(0.34 to 0.83) | 0.29(-0.00 to 0.58) |
| Canada | 50-54 | -0.70(-1.01 to -0.39) | 0.44(0.25 to 0.63) | 0.07(-0.16 to 0.29) |
| Canada | 55-59 | -1.12(-1.35 to -0.89) | 0.10(-0.05 to 0.25) | -0.32(-0.49 to -0.15) |
| Canada | 60-64 | -1.46(-1.63 to -1.28) | -0.29(-0.42 to -0.16) | -0.71(-0.86 to -0.57) |
| Canada | 65-69 | -1.52(-1.67 to -1.38) | -0.59(-0.70 to -0.48) | -0.93(-1.05 to -0.81) |
| Canada | 70-74 | -1.28(-1.41 to -1.15) | -0.73(-0.84 to -0.62) | -0.90(-1.01 to -0.78) |
| Canada | 75-79 | -0.91(-1.05 to -0.77) | -0.73(-0.86 to -0.60) | -0.73(-0.86 to -0.60) |
| Canada | 80-84 | -0.62(-0.79 to -0.44) | -0.70(-0.88 to -0.51) | -0.58(-0.75 to -0.41) |
| Canada | 85-89 | -0.48(-0.76 to -0.21) | -0.69(-1.02 to -0.36) | -0.51(-0.80 to -0.23) |
| Canada | 90-94 | -0.47(-0.97 to 0.03) | -0.69(-1.40 to 0.01) | -0.52(-1.08 to 0.03) |
| Canada | 95-99 | -0.51(-1.62 to 0.61) | -0.70(-2.47 to 1.10) | -0.56(-1.82 to 0.73) |
| Denmark | 30-34 | 1.00(-1.90 to 3.98) | 0.19(-1.04 to 1.44) | 0.32(-0.82 to 1.46) |
| Denmark | 35-39 | 1.08(-0.51 to 2.70) | 0.15(-0.54 to 0.85) | 0.28(-0.35 to 0.91) |
| Denmark | 40-44 | 1.13(0.08 to 2.19) | 0.08(-0.41 to 0.58) | 0.25(-0.20 to 0.70) |
| Denmark | 45-49 | 1.02(0.25 to 1.80) | -0.02(-0.42 to 0.38) | 0.19(-0.16 to 0.54) |
| Denmark | 50-54 | 0.75(0.18 to 1.33) | -0.14(-0.47 to 0.19) | 0.09(-0.19 to 0.38) |
| Denmark | 55-59 | 0.40(-0.05 to 0.85) | -0.29(-0.57 to 0.00) | -0.07(-0.31 to 0.17) |
| Denmark | 60-64 | 0.01(-0.34 to 0.36) | -0.51(-0.77 to -0.26) | -0.30(-0.51 to -0.10) |
| Denmark | 65-69 | -0.32(-0.60 to -0.05) | -0.78(-1.01 to -0.56) | -0.56(-0.73 to -0.38) |
| Denmark | 70-74 | -0.62(-0.85 to -0.39) | -1.07(-1.29 to -0.84) | -0.82(-0.98 to -0.66) |
| Denmark | 75-79 | -0.90(-1.14 to -0.67) | -1.34(-1.61 to -1.06) | -1.09(-1.27 to -0.92) |
| Denmark | 80-84 | -1.19(-1.47 to -0.90) | -1.51(-1.90 to -1.12) | -1.34(-1.57 to -1.11) |
| Denmark | 85-89 | -1.55(-1.96 to -1.13) | -1.62(-2.34 to -0.89) | -1.65(-2.00 to -1.29) |
| Denmark | 90-94 | -1.95(-2.67 to -1.22) | -1.75(-3.37 to -0.11) | -1.97(-2.62 to -1.32) |
| Denmark | 95-99 | -2.22(-3.83 to -0.58) | -1.86(-5.81 to 2.26) | -2.17(-3.67 to -0.66) |
| Finland | 30-34 | -0.97(-4.64 to 2.84) | -1.51(-2.82 to -0.19) | -1.40(-2.63 to -0.15) |
| Finland | 35-39 | -1.01(-2.96 to 0.98) | -1.50(-2.20 to -0.79) | -1.39(-2.05 to -0.72) |
| Finland | 40-44 | -1.13(-2.45 to 0.21) | -1.57(-2.08 to -1.05) | -1.46(-1.94 to -0.98) |
| Finland | 45-49 | -1.34(-2.29 to -0.37) | -1.62(-2.03 to -1.20) | -1.55(-1.93 to -1.17) |
| Finland | 50-54 | -1.61(-2.27 to -0.96) | -1.56(-1.89 to -1.23) | -1.60(-1.89 to -1.30) |
| Finland | 55-59 | -1.72(-2.18 to -1.26) | -1.39(-1.67 to -1.11) | -1.51(-1.74 to -1.27) |
| Finland | 60-64 | -1.63(-1.94 to -1.32) | -1.17(-1.40 to -0.93) | -1.32(-1.51 to -1.14) |
| Finland | 65-69 | -1.42(-1.65 to -1.20) | -1.01(-1.22 to -0.80) | -1.16(-1.31 to -1.01) |
| Finland | 70-74 | -1.17(-1.38 to -0.97) | -0.92(-1.15 to -0.69) | -1.03(-1.18 to -0.88) |
| Finland | 75-79 | -0.98(-1.21 to -0.74) | -0.79(-1.10 to -0.47) | -0.93(-1.11 to -0.74) |
| Finland | 80-84 | -0.94(-1.23 to -0.64) | -0.66(-1.13 to -0.18) | -0.91(-1.16 to -0.66) |
| Finland | 85-89 | -1.07(-1.51 to -0.63) | -0.72(-1.59 to 0.16) | -1.07(-1.46 to -0.68) |
| Finland | 90-94 | -1.26(-2.04 to -0.48) | -0.98(-2.92 to 1.00) | -1.31(-2.03 to -0.58) |
| Finland | 95-99 | -1.44(-3.30 to 0.46) | -1.23(-6.46 to 4.28) | -1.52(-3.27 to 0.27) |
| France | 30-34 | -0.73(-1.86 to 0.42) | -0.46(-0.94 to 0.01) | -0.54(-0.98 to -0.11) |
| France | 35-39 | -0.72(-1.32 to -0.12) | -0.45(-0.71 to -0.20) | -0.51(-0.74 to -0.28) |
| France | 40-44 | -0.69(-1.09 to -0.30) | -0.44(-0.62 to -0.26) | -0.49(-0.66 to -0.32) |
| France | 45-49 | -0.66(-0.94 to -0.38) | -0.44(-0.58 to -0.29) | -0.50(-0.63 to -0.37) |
| France | 50-54 | -0.63(-0.83 to -0.42) | -0.45(-0.56 to -0.33) | -0.52(-0.62 to -0.42) |
| France | 55-59 | -0.61(-0.76 to -0.46) | -0.45(-0.55 to -0.35) | -0.52(-0.60 to -0.43) |
| France | 60-64 | -0.61(-0.72 to -0.50) | -0.47(-0.55 to -0.38) | -0.51(-0.58 to -0.45) |
| France | 65-69 | -0.64(-0.72 to -0.55) | -0.50(-0.57 to -0.42) | -0.53(-0.58 to -0.47) |
| France | 70-74 | -0.70(-0.77 to -0.62) | -0.55(-0.63 to -0.48) | -0.60(-0.65 to -0.54) |
| France | 75-79 | -0.82(-0.90 to -0.74) | -0.64(-0.74 to -0.55) | -0.74(-0.80 to -0.68) |
| France | 80-84 | -1.00(-1.08 to -0.91) | -0.78(-0.90 to -0.65) | -0.95(-1.02 to -0.88) |
| France | 85-89 | -1.26(-1.38 to -1.14) | -0.98(-1.21 to -0.76) | -1.26(-1.37 to -1.16) |
| France | 90-94 | -1.55(-1.77 to -1.34) | -1.27(-1.81 to -0.72) | -1.63(-1.82 to -1.43) |
| France | 95-99 | -1.78(-2.28 to -1.27) | -1.50(-3.09 to 0.12) | -1.90(-2.38 to -1.42) |
| Germany | 30-34 | 0.21(-3.24 to 3.78) | 0.32(-0.28 to 0.93) | 0.25(-1.05 to 1.57) |
| Germany | 35-39 | 0.31(-1.59 to 2.24) | 0.34(0.00 to 0.69) | 0.24(-0.49 to 0.99) |
| Germany | 40-44 | 0.32(-1.02 to 1.68) | 0.30(0.04 to 0.57) | 0.23(-0.33 to 0.79) |
| Germany | 45-49 | 0.27(-0.72 to 1.27) | 0.24(0.03 to 0.45) | 0.20(-0.24 to 0.64) |
| Germany | 50-54 | 0.22(-0.48 to 0.92) | 0.17(0.01 to 0.34) | 0.17(-0.17 to 0.52) |
| Germany | 55-59 | 0.22(-0.29 to 0.74) | 0.13(-0.01 to 0.27) | 0.16(-0.12 to 0.44) |
| Germany | 60-64 | 0.31(-0.09 to 0.71) | 0.13(0.00 to 0.25) | 0.21(-0.02 to 0.45) |
| Germany | 65-69 | 0.38(0.06 to 0.70) | 0.13(0.01 to 0.25) | 0.31(0.10 to 0.53) |
| Germany | 70-74 | 0.09(-0.20 to 0.39) | -0.03(-0.16 to 0.10) | 0.18(-0.03 to 0.40) |
| Germany | 75-79 | -0.83(-1.15 to -0.52) | -0.50(-0.67 to -0.32) | -0.54(-0.79 to -0.29) |
| Germany | 80-84 | -1.96(-2.32 to -1.60) | -1.09(-1.32 to -0.85) | -1.57(-1.87 to -1.26) |
| Germany | 85-89 | -2.79(-3.30 to -2.28) | -1.54(-1.96 to -1.11) | -2.47(-2.93 to -2.01) |
| Germany | 90-94 | -3.18(-4.11 to -2.24) | -1.84(-2.86 to -0.82) | -3.05(-3.95 to -2.15) |
| Germany | 95-99 | -3.01(-5.22 to -0.75) | -2.04(-4.85 to 0.84) | -3.18(-5.36 to -0.94) |
| Greece | 30-34 | -1.46(-4.28 to 1.46) | -0.34(-1.56 to 0.90) | -0.55(-1.67 to 0.59) |
| Greece | 35-39 | -1.58(-2.97 to -0.18) | -0.39(-1.01 to 0.24) | -0.61(-1.18 to -0.04) |
| Greece | 40-44 | -1.71(-2.60 to -0.81) | -0.50(-0.95 to -0.06) | -0.75(-1.15 to -0.35) |
| Greece | 45-49 | -1.74(-2.38 to -1.10) | -0.53(-0.89 to -0.17) | -0.83(-1.14 to -0.52) |
| Greece | 50-54 | -1.68(-2.14 to -1.21) | -0.39(-0.68 to -0.09) | -0.79(-1.04 to -0.55) |
| Greece | 55-59 | -1.49(-1.84 to -1.14) | -0.12(-0.37 to 0.12) | -0.63(-0.83 to -0.43) |
| Greece | 60-64 | -1.19(-1.45 to -0.93) | 0.18(-0.02 to 0.39) | -0.39(-0.55 to -0.23) |
| Greece | 65-69 | -0.89(-1.09 to -0.68) | 0.34(0.16 to 0.53) | -0.23(-0.36 to -0.09) |
| Greece | 70-74 | -0.62(-0.80 to -0.44) | 0.18(-0.00 to 0.37) | -0.22(-0.35 to -0.09) |
| Greece | 75-79 | -0.48(-0.67 to -0.29) | -0.20(-0.42 to 0.02) | -0.34(-0.49 to -0.20) |
| Greece | 80-84 | -0.57(-0.80 to -0.34) | -0.63(-0.92 to -0.33) | -0.58(-0.76 to -0.40) |
| Greece | 85-89 | -0.90(-1.26 to -0.55) | -1.07(-1.56 to -0.57) | -0.95(-1.23 to -0.66) |
| Greece | 90-94 | -1.33(-2.05 to -0.61) | -1.47(-2.54 to -0.39) | -1.44(-2.04 to -0.85) |
| Greece | 95-99 | -1.68(-3.73 to 0.42) | -1.68(-4.75 to 1.50) | -2.08(-3.77 to -0.37) |
| Ireland | 30-34 | -1.29(-5.38 to 2.98) | -0.70(-2.43 to 1.05) | -0.99(-2.57 to 0.63) |
| Ireland | 35-39 | -1.21(-3.33 to 0.95) | -0.68(-1.59 to 0.24) | -0.90(-1.73 to -0.06) |
| Ireland | 40-44 | -1.13(-2.58 to 0.35) | -0.67(-1.35 to 0.01) | -0.82(-1.43 to -0.20) |
| Ireland | 45-49 | -1.05(-2.15 to 0.06) | -0.69(-1.26 to -0.13) | -0.82(-1.32 to -0.32) |
| Ireland | 50-54 | -1.02(-1.86 to -0.17) | -0.72(-1.21 to -0.24) | -0.85(-1.26 to -0.43) |
| Ireland | 55-59 | -1.03(-1.69 to -0.36) | -0.75(-1.16 to -0.33) | -0.86(-1.21 to -0.51) |
| Ireland | 60-64 | -1.10(-1.62 to -0.57) | -0.76(-1.12 to -0.39) | -0.86(-1.16 to -0.56) |
| Ireland | 65-69 | -1.24(-1.66 to -0.81) | -0.77(-1.10 to -0.43) | -0.89(-1.15 to -0.63) |
| Ireland | 70-74 | -1.43(-1.80 to -1.05) | -0.85(-1.18 to -0.51) | -1.02(-1.27 to -0.78) |
| Ireland | 75-79 | -1.64(-2.02 to -1.26) | -1.06(-1.47 to -0.66) | -1.30(-1.58 to -1.03) |
| Ireland | 80-84 | -1.78(-2.23 to -1.33) | -1.33(-1.91 to -0.74) | -1.60(-1.96 to -1.25) |
| Ireland | 85-89 | -1.77(-2.45 to -1.09) | -1.53(-2.65 to -0.39) | -1.81(-2.38 to -1.24) |
| Ireland | 90-94 | -1.64(-2.90 to -0.36) | -1.64(-4.38 to 1.17) | -1.87(-3.01 to -0.73) |
| Ireland | 95-99 | -1.48(-4.77 to 1.93) | -1.66(-9.60 to 6.97) | -1.81(-4.85 to 1.33) |
| Italy | 30-34 | -0.72(-3.52 to 2.16) | -1.24(-2.79 to 0.34) | -0.98(-2.62 to 0.70) |
| Italy | 35-39 | -0.55(-1.97 to 0.89) | -1.15(-1.96 to -0.34) | -0.88(-1.73 to -0.02) |
| Italy | 40-44 | -0.44(-1.32 to 0.46) | -1.05(-1.59 to -0.51) | -0.78(-1.34 to -0.22) |
| Italy | 45-49 | -0.41(-1.03 to 0.22) | -0.93(-1.34 to -0.51) | -0.69(-1.11 to -0.27) |
| Italy | 50-54 | -0.45(-0.91 to 0.02) | -0.77(-1.11 to -0.43) | -0.61(-0.94 to -0.29) |
| Italy | 55-59 | -0.51(-0.87 to -0.15) | -0.59(-0.88 to -0.30) | -0.54(-0.81 to -0.27) |
| Italy | 60-64 | -0.58(-0.86 to -0.30) | -0.40(-0.65 to -0.14) | -0.49(-0.71 to -0.27) |
| Italy | 65-69 | -0.68(-0.91 to -0.45) | -0.28(-0.51 to -0.06) | -0.52(-0.71 to -0.33) |
| Italy | 70-74 | -0.75(-0.95 to -0.55) | -0.28(-0.50 to -0.05) | -0.61(-0.79 to -0.44) |
| Italy | 75-79 | -0.83(-1.03 to -0.62) | -0.38(-0.66 to -0.11) | -0.74(-0.93 to -0.55) |
| Italy | 80-84 | -0.86(-1.10 to -0.63) | -0.54(-0.88 to -0.19) | -0.82(-1.05 to -0.60) |
| Italy | 85-89 | -0.80(-1.14 to -0.47) | -0.70(-1.29 to -0.12) | -0.81(-1.15 to -0.47) |
| Italy | 90-94 | -0.70(-1.31 to -0.08) | -0.82(-2.07 to 0.45) | -0.70(-1.34 to -0.06) |
| Italy | 95-99 | -0.64(-2.13 to 0.87) | -0.85(-4.20 to 2.62) | -0.57(-2.15 to 1.04) |
| Luxembourg | 30-34 | 2.21(-8.21 to 13.81) | -0.05(-4.65 to 4.77) | 0.31(-3.91 to 4.71) |
| Luxembourg | 35-39 | 1.11(-4.82 to 7.40) | 0.08(-2.56 to 2.79) | 0.22(-2.18 to 2.69) |
| Luxembourg | 40-44 | -0.34(-4.59 to 4.11) | 0.28(-1.77 to 2.36) | 0.10(-1.74 to 1.97) |
| Luxembourg | 45-49 | -1.20(-4.46 to 2.16) | 0.34(-1.37 to 2.09) | -0.04(-1.55 to 1.49) |
| Luxembourg | 50-54 | -1.13(-3.55 to 1.35) | 0.25(-1.18 to 1.71) | -0.07(-1.29 to 1.17) |
| Luxembourg | 55-59 | -0.71(-2.54 to 1.15) | 0.07(-1.14 to 1.29) | -0.08(-1.08 to 0.93) |
| Luxembourg | 60-64 | -0.34(-1.70 to 1.04) | -0.12(-1.11 to 0.88) | -0.09(-0.89 to 0.71) |
| Luxembourg | 65-69 | -0.12(-1.18 to 0.95) | -0.13(-0.98 to 0.72) | -0.01(-0.67 to 0.65) |
| Luxembourg | 70-74 | -0.09(-0.99 to 0.83) | 0.00(-0.88 to 0.88) | 0.07(-0.55 to 0.69) |
| Luxembourg | 75-79 | -0.18(-1.08 to 0.73) | 0.21(-0.87 to 1.30) | 0.05(-0.64 to 0.74) |
| Luxembourg | 80-84 | -0.40(-1.47 to 0.69) | 0.29(-1.24 to 1.83) | -0.13(-1.00 to 0.75) |
| Luxembourg | 85-89 | -0.96(-2.61 to 0.73) | -0.01(-2.96 to 3.02) | -0.71(-2.14 to 0.74) |
| Luxembourg | 90-94 | -1.73(-4.99 to 1.64) | -0.72(-7.90 to 7.03) | -1.56(-4.52 to 1.50) |
| Luxembourg | 95-99 | -2.23(-10.65 to 6.99) | -1.37(-21.98 to 24.67) | -2.12(-10.01 to 6.45) |
| Netherlands | 30-34 | -0.81(-3.00 to 1.43) | -0.48(-1.38 to 0.42) | -0.57(-1.41 to 0.27) |
| Netherlands | 35-39 | -0.81(-1.98 to 0.38) | -0.46(-0.96 to 0.04) | -0.57(-1.03 to -0.11) |
| Netherlands | 40-44 | -0.79(-1.55 to -0.03) | -0.46(-0.82 to -0.10) | -0.58(-0.90 to -0.25) |
| Netherlands | 45-49 | -0.76(-1.27 to -0.24) | -0.50(-0.78 to -0.22) | -0.59(-0.83 to -0.35) |
| Netherlands | 50-54 | -0.72(-1.08 to -0.36) | -0.59(-0.81 to -0.36) | -0.63(-0.82 to -0.44) |
| Netherlands | 55-59 | -0.69(-0.96 to -0.43) | -0.68(-0.87 to -0.48) | -0.68(-0.83 to -0.52) |
| Netherlands | 60-64 | -0.68(-0.88 to -0.48) | -0.74(-0.91 to -0.58) | -0.70(-0.82 to -0.57) |
| Netherlands | 65-69 | -0.70(-0.86 to -0.55) | -0.78(-0.93 to -0.63) | -0.72(-0.83 to -0.61) |
| Netherlands | 70-74 | -0.80(-0.93 to -0.66) | -0.78(-0.93 to -0.63) | -0.78(-0.88 to -0.69) |
| Netherlands | 75-79 | -0.97(-1.11 to -0.82) | -0.76(-0.94 to -0.59) | -0.91(-1.02 to -0.80) |
| Netherlands | 80-84 | -1.26(-1.42 to -1.09) | -0.78(-1.04 to -0.53) | -1.16(-1.30 to -1.02) |
| Netherlands | 85-89 | -1.72(-1.97 to -1.48) | -0.96(-1.44 to -0.48) | -1.62(-1.83 to -1.40) |
| Netherlands | 90-94 | -2.21(-2.64 to -1.77) | -1.32(-2.38 to -0.25) | -2.10(-2.50 to -1.71) |
| Netherlands | 95-99 | -2.49(-3.51 to -1.46) | -1.63(-4.35 to 1.16) | -2.39(-3.34 to -1.43) |
| Norway | 30-34 | -0.60(-4.68 to 3.65) | -0.24(-1.93 to 1.47) | -0.28(-1.84 to 1.31) |
| Norway | 35-39 | -0.56(-2.78 to 1.71) | -0.22(-1.15 to 0.72) | -0.24(-1.10 to 0.63) |
| Norway | 40-44 | -0.46(-1.89 to 0.98) | -0.14(-0.78 to 0.51) | -0.16(-0.75 to 0.42) |
| Norway | 45-49 | -0.33(-1.32 to 0.66) | -0.03(-0.51 to 0.46) | -0.07(-0.50 to 0.37) |
| Norway | 50-54 | -0.21(-0.92 to 0.51) | 0.09(-0.31 to 0.49) | 0.05(-0.29 to 0.40) |
| Norway | 55-59 | -0.17(-0.72 to 0.38) | 0.14(-0.20 to 0.48) | 0.10(-0.19 to 0.39) |
| Norway | 60-64 | -0.23(-0.65 to 0.19) | 0.07(-0.23 to 0.37) | 0.03(-0.21 to 0.27) |
| Norway | 65-69 | -0.37(-0.70 to -0.04) | -0.11(-0.37 to 0.16) | -0.14(-0.35 to 0.07) |
| Norway | 70-74 | -0.57(-0.85 to -0.29) | -0.36(-0.63 to -0.10) | -0.41(-0.60 to -0.22) |
| Norway | 75-79 | -0.81(-1.09 to -0.52) | -0.65(-0.96 to -0.33) | -0.73(-0.93 to -0.52) |
| Norway | 80-84 | -1.04(-1.37 to -0.72) | -0.90(-1.34 to -0.47) | -1.03(-1.28 to -0.77) |
| Norway | 85-89 | -1.30(-1.72 to -0.87) | -1.14(-1.86 to -0.41) | -1.32(-1.68 to -0.96) |
| Norway | 90-94 | -1.53(-2.19 to -0.86) | -1.38(-2.75 to 0.02) | -1.57(-2.16 to -0.98) |
| Norway | 95-99 | -1.71(-2.92 to -0.49) | -1.54(-4.38 to 1.39) | -1.71(-2.82 to -0.59) |
| Portugal | 30-34 | -1.48(-4.42 to 1.54) | -1.36(-2.73 to 0.03) | -1.34(-2.70 to 0.05) |
| Portugal | 35-39 | -1.60(-3.03 to -0.14) | -1.48(-2.18 to -0.77) | -1.49(-2.18 to -0.79) |
| Portugal | 40-44 | -1.62(-2.51 to -0.71) | -1.56(-2.04 to -1.06) | -1.57(-2.04 to -1.10) |
| Portugal | 45-49 | -1.38(-2.02 to -0.73) | -1.39(-1.79 to -1.00) | -1.38(-1.75 to -1.01) |
| Portugal | 50-54 | -0.92(-1.38 to -0.45) | -0.96(-1.28 to -0.64) | -0.93(-1.22 to -0.64) |
| Portugal | 55-59 | -0.40(-0.74 to -0.06) | -0.47(-0.74 to -0.21) | -0.42(-0.65 to -0.19) |
| Portugal | 60-64 | 0.12(-0.13 to 0.37) | -0.03(-0.25 to 0.19) | 0.05(-0.13 to 0.23) |
| Portugal | 65-69 | 0.35(0.16 to 0.54) | 0.20(0.01 to 0.39) | 0.27(0.13 to 0.42) |
| Portugal | 70-74 | 0.30(0.14 to 0.47) | 0.17(-0.02 to 0.36) | 0.23(0.09 to 0.36) |
| Portugal | 75-79 | 0.04(-0.13 to 0.21) | -0.02(-0.25 to 0.21) | -0.01(-0.16 to 0.14) |
| Portugal | 80-84 | -0.36(-0.58 to -0.15) | -0.29(-0.62 to 0.03) | -0.37(-0.56 to -0.17) |
| Portugal | 85-89 | -0.85(-1.19 to -0.50) | -0.63(-1.27 to 0.02) | -0.82(-1.16 to -0.49) |
| Portugal | 90-94 | -1.30(-2.03 to -0.57) | -1.00(-2.71 to 0.74) | -1.30(-2.03 to -0.56) |
| Portugal | 95-99 | -1.59(-3.58 to 0.44) | -1.25(-6.30 to 4.08) | -1.60(-3.64 to 0.47) |
| Spain | 30-34 | -0.30(-2.22 to 1.66) | 0.43(-0.27 to 1.13) | 0.26(-0.65 to 1.17) |
| Spain | 35-39 | -0.36(-1.30 to 0.60) | 0.31(-0.05 to 0.67) | 0.20(-0.26 to 0.66) |
| Spain | 40-44 | -0.54(-1.14 to 0.06) | 0.10(-0.15 to 0.36) | 0.00(-0.32 to 0.32) |
| Spain | 45-49 | -0.78(-1.21 to -0.35) | -0.05(-0.26 to 0.16) | -0.21(-0.47 to 0.04) |
| Spain | 50-54 | -0.99(-1.31 to -0.67) | -0.05(-0.23 to 0.12) | -0.34(-0.54 to -0.13) |
| Spain | 55-59 | -0.93(-1.18 to -0.68) | 0.14(-0.01 to 0.29) | -0.24(-0.41 to -0.07) |
| Spain | 60-64 | -0.60(-0.80 to -0.41) | 0.49(0.36 to 0.62) | 0.06(-0.09 to 0.20) |
| Spain | 65-69 | -0.29(-0.44 to -0.13) | 0.82(0.69 to 0.94) | 0.32(0.20 to 0.45) |
| Spain | 70-74 | -0.11(-0.25 to 0.03) | 0.94(0.82 to 1.06) | 0.40(0.28 to 0.51) |
| Spain | 75-79 | -0.10(-0.24 to 0.04) | 0.83(0.68 to 0.98) | 0.25(0.12 to 0.38) |
| Spain | 80-84 | -0.38(-0.55 to -0.22) | 0.52(0.31 to 0.73) | -0.14(-0.30 to 0.02) |
| Spain | 85-89 | -1.05(-1.28 to -0.81) | -0.13(-0.51 to 0.24) | -0.89(-1.13 to -0.64) |
| Spain | 90-94 | -1.83(-2.26 to -1.40) | -1.05(-1.87 to -0.23) | -1.72(-2.18 to -1.26) |
| Spain | 95-99 | -2.39(-3.46 to -1.31) | -1.73(-3.82 to 0.40) | -2.25(-3.40 to -1.09) |
| Sweden | 30-34 | 0.61(-1.43 to 2.70) | 0.52(-0.33 to 1.38) | 0.46(-0.38 to 1.31) |
| Sweden | 35-39 | 0.52(-0.62 to 1.67) | 0.72(0.23 to 1.21) | 0.62(0.14 to 1.10) |
| Sweden | 40-44 | 0.34(-0.43 to 1.12) | 0.89(0.52 to 1.25) | 0.71(0.36 to 1.06) |
| Sweden | 45-49 | 0.38(-0.17 to 0.94) | 1.11(0.82 to 1.40) | 0.89(0.62 to 1.17) |
| Sweden | 50-54 | 0.70(0.30 to 1.10) | 1.39(1.15 to 1.63) | 1.19(0.97 to 1.41) |
| Sweden | 55-59 | 1.22(0.91 to 1.52) | 1.62(1.41 to 1.83) | 1.51(1.33 to 1.70) |
| Sweden | 60-64 | 1.72(1.50 to 1.95) | 1.71(1.53 to 1.89) | 1.75(1.60 to 1.90) |
| Sweden | 65-69 | 1.65(1.48 to 1.83) | 1.48(1.32 to 1.64) | 1.59(1.46 to 1.71) |
| Sweden | 70-74 | 1.02(0.88 to 1.17) | 0.86(0.71 to 1.01) | 0.96(0.85 to 1.07) |
| Sweden | 75-79 | 0.23(0.09 to 0.37) | 0.11(-0.06 to 0.28) | 0.17(0.05 to 0.28) |
| Sweden | 80-84 | -0.38(-0.54 to -0.22) | -0.50(-0.73 to -0.27) | -0.48(-0.62 to -0.34) |
| Sweden | 85-89 | -0.67(-0.90 to -0.43) | -0.94(-1.33 to -0.54) | -0.87(-1.08 to -0.65) |
| Sweden | 90-94 | -0.70(-1.10 to -0.30) | -1.26(-2.10 to -0.40) | -1.04(-1.42 to -0.65) |
| Sweden | 95-99 | -0.64(-1.50 to 0.23) | -1.41(-3.56 to 0.79) | -1.06(-1.92 to -0.19) |
| UK | 30-34 | 0.43(-0.67 to 1.55) | 0.10(-0.36 to 0.56) | 0.10(-0.36 to 0.57) |
| UK | 35-39 | 0.35(-0.26 to 0.97) | 0.13(-0.13 to 0.39) | 0.14(-0.12 to 0.41) |
| UK | 40-44 | 0.20(-0.23 to 0.63) | 0.12(-0.07 to 0.32) | 0.13(-0.07 to 0.32) |
| UK | 45-49 | 0.00(-0.31 to 0.31) | 0.05(-0.11 to 0.21) | 0.03(-0.12 to 0.18) |
| UK | 50-54 | -0.19(-0.42 to 0.04) | -0.07(-0.20 to 0.06) | -0.11(-0.23 to 0.01) |
| UK | 55-59 | -0.30(-0.47 to -0.13) | -0.18(-0.28 to -0.07) | -0.20(-0.30 to -0.10) |
| UK | 60-64 | -0.31(-0.44 to -0.18) | -0.23(-0.32 to -0.14) | -0.22(-0.31 to -0.14) |
| UK | 65-69 | -0.28(-0.38 to -0.18) | -0.23(-0.32 to -0.15) | -0.21(-0.28 to -0.14) |
| UK | 70-74 | -0.26(-0.34 to -0.17) | -0.25(-0.33 to -0.17) | -0.22(-0.28 to -0.16) |
| UK | 75-79 | -0.29(-0.37 to -0.20) | -0.31(-0.41 to -0.21) | -0.30(-0.37 to -0.23) |
| UK | 80-84 | -0.47(-0.57 to -0.37) | -0.52(-0.66 to -0.38) | -0.55(-0.64 to -0.46) |
| UK | 85-89 | -0.92(-1.07 to -0.78) | -1.22(-1.48 to -0.96) | -1.13(-1.26 to -0.99) |
| UK | 90-94 | -1.55(-1.80 to -1.29) | -2.44(-3.04 to -1.83) | -1.91(-2.16 to -1.65) |
| UK | 95-99 | -2.15(-2.74 to -1.55) | -3.55(-5.08 to -2.00) | -2.56(-3.16 to -1.95) |
| USA | 30-34 | 0.38(-0.53 to 1.31) | -0.78(-1.73 to 0.19) | -0.47(-1.33 to 0.40) |
| USA | 35-39 | 0.37(-0.13 to 0.88) | -0.71(-1.25 to -0.18) | -0.41(-0.89 to 0.07) |
| USA | 40-44 | 0.39(0.02 to 0.75) | -0.40(-0.79 to -0.01) | -0.17(-0.52 to 0.19) |
| USA | 45-49 | 0.43(0.16 to 0.71) | 0.04(-0.28 to 0.35) | 0.17(-0.11 to 0.45) |
| USA | 50-54 | 0.49(0.28 to 0.70) | 0.48(0.23 to 0.73) | 0.50(0.29 to 0.72) |
| USA | 55-59 | 0.53(0.37 to 0.69) | 0.73(0.52 to 0.94) | 0.69(0.51 to 0.86) |
| USA | 60-64 | 0.55(0.42 to 0.67) | 0.77(0.59 to 0.95) | 0.71(0.57 to 0.86) |
| USA | 65-69 | 0.56(0.46 to 0.66) | 0.74(0.58 to 0.90) | 0.69(0.57 to 0.82) |
| USA | 70-74 | 0.58(0.49 to 0.67) | 0.83(0.67 to 0.99) | 0.72(0.61 to 0.84) |
| USA | 75-79 | 0.52(0.43 to 0.61) | 0.95(0.76 to 1.13) | 0.72(0.59 to 0.84) |
| USA | 80-84 | 0.24(0.13 to 0.35) | 0.90(0.64 to 1.17) | 0.50(0.34 to 0.67) |
| USA | 85-89 | -0.41(-0.57 to -0.25) | 0.53(0.09 to 0.97) | -0.07(-0.32 to 0.18) |
| USA | 90-94 | -1.34(-1.60 to -1.07) | -0.19(-1.05 to 0.67) | -0.97(-1.41 to -0.53) |
| USA | 95-99 | -2.19(-2.73 to -1.65) | -0.96(-2.91 to 1.02) | -1.83(-2.74 to -0.92) |

CI:confidence interval;

Local drifts indicate the annual percentage change over time specific to the age group.

All of Local drifts were statistically significant (p < 0.05).

Finland, Ireland, Italy, Luxembourg, and Norway was excluded because these data didn’t meet the APC model’s requirements.

Table S10. Wald Tests.

| Country | Parameter | Female_X2 | Female_df | Female_P_Value | Male_X2 | Male_df | Male_P_Value | Both_X2 | Both_df | Both_P_Value |
| --- | --- | --- | --- | --- | --- | --- | --- | --- | --- | --- |
| Australia | NetDrift = 0 | 6.04830188470425 | 1 | 0.0139196774344075 | 4.9187447393014 | 1 | 0.0265668128369928 | 2.41924390797411 | 1 | 0.119852766224962 |
| Australia | All Age Deviations = 0 | 4032.40623048423 | 12 | 0 | 5836.80221529631 | 12 | 0 | 8953.8156314785 | 12 | 0 |
| Australia | All Period Deviations = 0 | 4.21886894671831 | 4 | 0.377194765690784 | 11.3661266875885 | 4 | 0.0227432500896986 | 9.79705893450633 | 4 | 0.0439885287075894 |
| Australia | All Cohort Deviations = 0 | 70.8034378147245 | 17 | 1.5677448889121e-08 | 26.5029806298155 | 17 | 0.065771354346327 | 95.9026442394138 | 17 | 5.07929853255081e-13 |
| Australia | All Period RR = 1 | 11.7154042711713 | 5 | 0.0389024929235271 | 16.812822188131 | 5 | 0.00486881170252422 | 12.3243065342663 | 5 | 0.0306043470622933 |
| Australia | All Cohort RR = 1 | 97.6637747711236 | 18 | 5.92140163350552e-13 | 26.5727921737507 | 18 | 0.0873643831978605 | 105.339228162252 | 18 | 2.30532650407287e-14 |
| Australia | All Local Drifts = Net Drift | 68.8260625026383 | 14 | 3.14662904919078e-09 | 26.4413481145166 | 14 | 0.0227349366873986 | 95.5370044797804 | 14 | 3.37906877926377e-14 |
| Austria | NetDrift = 0 | 152.724782298591 | 1 | 4.39968590491614e-35 | 220.7267591 | 1 | 6.27856021178667e-50 | 733.055019453241 | 1 | 1.94046761000555e-161 |
| Austria | All Age Deviations = 0 | 5582.64424507848 | 12 | 0 | 4413.74760373327 | 12 | 0 | 9976.41985467563 | 12 | 0 |
| Austria | All Period Deviations = 0 | 48.5508866903788 | 4 | 7.24448790229815e-10 | 51.2346904357092 | 4 | 1.99385268809167e-10 | 111.029548715647 | 4 | 4.38936635353798e-23 |
| Austria | All Cohort Deviations = 0 | 141.785575264614 | 17 | 9.81192751319445e-22 | 29.9402001119275 | 17 | 0.0267795802397712 | 194.406437551319 | 17 | 3.80247763906073e-32 |
| Austria | All Period RR = 1 | 178.63302234412 | 5 | 1.04801253585454e-36 | 250.377307389527 | 5 | 4.56208071622609e-52 | 767.844812169528 | 5 | 1.04482184598533e-163 |
| Austria | All Cohort RR = 1 | 1088.21075029932 | 18 | 9.64722521133748e-220 | 1202.2806336122 | 18 | 3.63222258290791e-244 | 2472.96674076848 | 18 | 0 |
| Austria | All Local Drifts = Net Drift | 141.591095219345 | 14 | 3.42332350187652e-23 | 29.643973716919 | 14 | 0.00854094628015 | 193.348370609362 | 14 | 1.25020904393144e-33 |
| Belgium | NetDrift = 0 | 11.8462832196773 | 1 | 0.000577765478643615 | 11.3035692741351 | 1 | 0.000773582353256451 | 28.8827664527238 | 1 | 7.68940239175965e-08 |
| Belgium | All Age Deviations = 0 | 4023.07476891847 | 12 | 0 | 1806.36745428728 | 12 | 0 | 2963.95067056667 | 12 | 0 |
| Belgium | All Period Deviations = 0 | 28.3362981484225 | 4 | 1.06606485601061e-05 | 10.1129901359326 | 4 | 0.0385667872502219 | 19.5508230274039 | 4 | 0.000612387879049757 |
| Belgium | All Cohort Deviations = 0 | 130.410265083953 | 17 | 1.56248658037381e-19 | 99.7718579527134 | 17 | 9.80611411026869e-14 | 135.806097661073 | 17 | 1.41914241396242e-20 |
| Belgium | All Period RR = 1 | 37.8449688882433 | 5 | 4.05370611243023e-07 | 20.7205385019951 | 5 | 0.000914674829234576 | 46.1203742560311 | 5 | 8.58376607471057e-09 |
| Belgium | All Cohort RR = 1 | 440.644677355296 | 18 | 2.95228537412157e-82 | 181.420050025804 | 18 | 5.01719436522831e-29 | 327.644607210904 | 18 | 9.63703568047134e-59 |
| Belgium | All Local Drifts = Net Drift | 129.512543650073 | 14 | 8.48334599560842e-21 | 99.3474694873371 | 14 | 6.32422833249261e-15 | 135.610808522551 | 14 | 5.27617994831306e-22 |
| Canada | NetDrift = 0 | 124.170037681255 | 1 | 7.7325147394736e-29 | 16.573780708163 | 1 | 4.67934939535549e-05 | 50.4751273580127 | 1 | 1.20684667758604e-12 |
| Canada | All Age Deviations = 0 | 8891.92892015652 | 12 | 0 | 10481.6632601919 | 12 | 0 | 10555.5941648837 | 12 | 0 |
| Canada | All Period Deviations = 0 | 99.8381450638787 | 4 | 1.06488508001014e-20 | 24.8201767277018 | 4 | 5.4676252849453e-05 | 52.3013916017097 | 4 | 1.19310338991409e-10 |
| Canada | All Cohort Deviations = 0 | 90.7380272037585 | 17 | 4.48052027649545e-12 | 136.623163486766 | 17 | 9.85891936798185e-21 | 86.2471107451687 | 17 | 2.92023302458247e-11 |
| Canada | All Period RR = 1 | 202.339523074847 | 5 | 8.97209265723906e-42 | 37.168167599738 | 5 | 5.54206224770499e-07 | 91.3944148795399 | 5 | 3.42238843444854e-18 |
| Canada | All Cohort RR = 1 | 592.46585377866 | 18 | 3.36564899750308e-114 | 260.336620588687 | 18 | 6.40413773503135e-45 | 341.696865924728 | 18 | 1.19545677308943e-61 |
| Canada | All Local Drifts = Net Drift | 88.8300197969783 | 14 | 6.30980109275136e-13 | 136.320842458724 | 14 | 3.8153400192054e-22 | 85.1714436377979 | 14 | 3.07347448967859e-12 |
| Denmark | NetDrift = 0 | 1.84317102283689 | 1 | 0.174579738319963 | 30.9245847401786 | 1 | 2.68252030521395e-08 | 56.6741581728107 | 1 | 5.1435059485771e-14 |
| Denmark | All Age Deviations = 0 | 2851.640916 | 12 | 0 | 2154.68838175483 | 12 | 0 | 4580.96962929406 | 12 | 0 |
| Denmark | All Period Deviations = 0 | 20.5707518898842 | 4 | 0.00038515364647243 | 19.4936443634622 | 4 | 0.000628476399057171 | 38.8591861377586 | 4 | 7.44894316581995e-08 |
| Denmark | All Cohort Deviations = 0 | 58.1130497076037 | 17 | 2.14522667075877e-06 | 44.563276197919 | 17 | 0.000282742667011497 | 89.4565442210738 | 17 | 7.663588952088e-12 |
| Denmark | All Period RR = 1 | 23.9992063470748 | 5 | 0.000217189200136114 | 50.4054801457795 | 5 | 1.14474095968863e-09 | 100.620331285378 | 5 | 3.91113736467175e-20 |
| Denmark | All Cohort RR = 1 | 117.041948290872 | 18 | 1.51416926859846e-16 | 145.876523907293 | 18 | 4.68917759080881e-22 | 245.373304986896 | 18 | 7.10799295884402e-42 |
| Denmark | All Local Drifts = Net Drift | 58.0508264025234 | 14 | 2.5697899794077e-07 | 44.4896039022734 | 14 | 4.93486936535787e-05 | 89.4012774510587 | 14 | 4.92337529371356e-13 |
| Finland | NetDrift = 0 | 62.1033190498079 | 1 | 3.25901660065227e-15 | 55.7277516771767 | 1 | 8.32349485023261e-14 | 250.039903234506 | 1 | 2.54530843996375e-56 |
| Finland | All Age Deviations = 0 | 4194.89564252375 | 12 | 0 | 2391.17840283651 | 12 | 0 | 6295.84599663719 | 12 | 0 |
| Finland | All Period Deviations = 0 | 7.02580707015529 | 4 | 0.134530708588025 | 12.4778795626485 | 4 | 0.0141298595144765 | 15.4526950700585 | 4 | 0.00384877395906257 |
| Finland | All Cohort Deviations = 0 | 14.5322383703769 | 17 | 0.629133994185482 | 16.0665005197242 | 17 | 0.519125592036002 | 19.2029187520106 | 17 | 0.317009939820152 |
| Finland | All Period RR = 1 | 68.4076412790481 | 5 | 2.19709148171524e-13 | 69.8821584840917 | 5 | 1.08432009214719e-13 | 268.296361143022 | 5 | 6.49790594055513e-56 |
| Finland | All Cohort RR = 1 | 268.020146658723 | 18 | 1.73085502742859e-46 | 226.337454922628 | 18 | 5.09646568401526e-38 | 476.1645752 | 18 | 1.05986676775465e-89 |
| Finland | All Local Drifts = Net Drift | 12.9888803047418 | 14 | 0.527399390282973 | 15.4394293452086 | 14 | 0.348790697191142 | 18.1471012668932 | 14 | 0.200162988089531 |
| France | NetDrift = 0 | 255.586489168732 | 1 | 1.57248774246347e-57 | 166.950268444553 | 1 | 3.42998989720759e-38 | 764.148956557977 | 1 | 3.36470479190651e-168 |
| France | All Age Deviations = 0 | 28574.4745986467 | 12 | 0 | 21039.6697832656 | 12 | 0 | 46454.2643489138 | 12 | 0 |
| France | All Period Deviations = 0 | 59.2913412660248 | 4 | 4.08712538683905e-12 | 33.3526514580833 | 4 | 1.0114763980893e-06 | 81.5029965648249 | 4 | 8.36606494654051e-17 |
| France | All Cohort Deviations = 0 | 90.9796059516789 | 17 | 4.04869855632407e-12 | 27.7627237842714 | 17 | 0.0477882052556607 | 199.850704307049 | 17 | 3.06800865947316e-33 |
| France | All Period RR = 1 | 295.982874989042 | 5 | 7.31557409234252e-62 | 192.712479798955 | 5 | 1.02788215803835e-39 | 810.426937194979 | 5 | 6.41982012867003e-173 |
| France | All Cohort RR = 1 | 1086.71174908143 | 18 | 2.01895252221254e-219 | 480.540148803708 | 18 | 1.27865109368824e-90 | 1597.60161706645 | 18 | 0 |
| France | All Local Drifts = Net Drift | 90.6336552870804 | 14 | 2.88048115613095e-13 | 27.7211102007718 | 14 | 0.0154900051566377 | 199.715354763942 | 14 | 6.2797222972224e-35 |
| Germany | NetDrift = 0 | 6.96915727114419 | 1 | 0.0082926542126387 | 10.1516654794473 | 1 | 0.00144170933903942 | 18.0127180518437 | 1 | 2.19434055781357e-05 |
| Germany | All Age Deviations = 0 | 2203.23256013293 | 12 | 0 | 9645.48260162404 | 12 | 0 | 3733.05460445806 | 12 | 0 |
| Germany | All Period Deviations = 0 | 3.88560649988271 | 4 | 0.421708637235713 | 307.759352155874 | 4 | 2.29562123786706e-65 | 49.066644995091 | 4 | 5.65484554442025e-10 |
| Germany | All Cohort Deviations = 0 | 132.719454053721 | 17 | 5.60542009601529e-20 | 113.325679733234 | 17 | 2.84812617424764e-16 | 155.084020559486 | 17 | 2.4649133173081e-24 |
| Germany | All Period RR = 1 | 10.8529058102906 | 5 | 0.0543756993176652 | 319.487610948615 | 5 | 6.45235454513602e-67 | 68.6967113999616 | 5 | 1.91313416997045e-13 |
| Germany | All Cohort RR = 1 | 164.330868466001 | 18 | 1.17997403486904e-25 | 114.826569818148 | 18 | 3.94562606004144e-16 | 164.697997409598 | 18 | 9.99547234862783e-26 |
| Germany | All Local Drifts = Net Drift | 129.801145200464 | 14 | 7.44051834183916e-21 | 113.08012249221 | 14 | 1.41095199611746e-17 | 153.038713718276 | 14 | 1.77139575119274e-25 |
| Greece | NetDrift = 0 | 86.358398986435 | 1 | 1.50109649534347e-20 | 10.5222905300841 | 1 | 0.00117943207196419 | 83.0654317684476 | 1 | 7.93818042502761e-20 |
| Greece | All Age Deviations = 0 | 3990.40148177361 | 12 | 0 | 3145.37821712008 | 12 | 0 | 6729.19595577824 | 12 | 0 |
| Greece | All Period Deviations = 0 | 82.1273521531859 | 4 | 6.16849614473007e-17 | 57.822518351207 | 4 | 8.31446617960536e-12 | 144.724803636118 | 4 | 2.74714595808671e-30 |
| Greece | All Cohort Deviations = 0 | 37.6220202550616 | 17 | 0.0027691691919394 | 52.6278755279281 | 17 | 1.63636582026197e-05 | 42.4619550845218 | 17 | 0.000574978800256177 |
| Greece | All Period RR = 1 | 177.379111193457 | 5 | 1.94138728839163e-36 | 70.6200153624472 | 5 | 7.61349812305908e-14 | 241.162069823269 | 5 | 4.32509306969019e-50 |
| Greece | All Cohort RR = 1 | 189.806969092995 | 18 | 1.08261333959849e-30 | 54.4574895547998 | 18 | 1.55696376212985e-05 | 134.433739422881 | 18 | 7.52435088026788e-20 |
| Greece | All Local Drifts = Net Drift | 37.0328501212795 | 14 | 0.00072934454178058 | 51.9803784199435 | 14 | 2.83361008263194e-06 | 42.2533790802909 | 14 | 0.000112688333307323 |
| Ireland | NetDrift = 0 | 38.0664370325461 | 1 | 6.83762511993622e-10 | 16.6872438521907 | 1 | 4.40763785625648e-05 | 83.2729136673813 | 1 | 7.14722465458911e-20 |
| Ireland | All Age Deviations = 0 | 795.52912712583 | 12 | 1.50478321521318e-162 | 956.063883968855 | 12 | 5.20026586628502e-197 | 1674.51109290819 | 12 | 0 |
| Ireland | All Period Deviations = 0 | 19.7840824851232 | 4 | 0.000550871558994967 | 3.45610277585333 | 4 | 0.484584328140821 | 12.4628274630241 | 4 | 0.0142218014612212 |
| Ireland | All Cohort Deviations = 0 | 4.39662689056185 | 17 | 0.99902864851015 | 3.84710315295076 | 17 | 0.999603074261084 | 14.1699795539018 | 17 | 0.655035315171212 |
| Ireland | All Period RR = 1 | 52.1336957489465 | 5 | 5.06467748471218e-10 | 19.3261034318171 | 5 | 0.00167096454178428 | 90.2067993714521 | 5 | 6.07969383144418e-18 |
| Ireland | All Cohort RR = 1 | 126.473630964099 | 18 | 2.49165375724085e-18 | 63.5068414908591 | 18 | 5.48271090796476e-07 | 180.992412057411 | 18 | 6.09843986140091e-29 |
| Ireland | All Local Drifts = Net Drift | 4.37612242604808 | 14 | 0.992744828052215 | 3.79826085937529 | 14 | 0.996562350979408 | 14.0577794244538 | 14 | 0.445415352017098 |
| Italy | NetDrift = 0 | 25.9201080105493 | 1 | 3.55843082145014e-07 | 28.4706837105831 | 1 | 9.51280373601439e-08 | 56.450210217717 | 1 | 5.76395615963989e-14 |
| Italy | All Age Deviations = 0 | 2452.73581182166 | 12 | 0 | 1418.23548704696 | 12 | 1.62763185340846e-296 | 2585.16853007613 | 12 | 0 |
| Italy | All Period Deviations = 0 | 8.06381934324687 | 4 | 0.0892681869995401 | 83.3226420002492 | 4 | 3.44153729319249e-17 | 34.0044621810785 | 4 | 7.4362021894427e-07 |
| Italy | All Cohort Deviations = 0 | 3.786580935 | 17 | 0.999643815909686 | 11.819922821119 | 17 | 0.810916774654327 | 5.74426998683328 | 17 | 0.994745724622558 |
| Italy | All Period RR = 1 | 31.1759179156763 | 5 | 8.64707693821311e-06 | 109.613307773877 | 5 | 4.94558200292795e-22 | 83.6623783185201 | 5 | 1.43561331353419e-16 |
| Italy | All Cohort RR = 1 | 111.864847854904 | 18 | 1.4133127253202e-15 | 51.1579201140712 | 18 | 5.03452254512307e-05 | 119.777188364219 | 18 | 4.62363568241068e-17 |
| Italy | All Local Drifts = Net Drift | 3.57595924029078 | 14 | 0.997523104 | 11.5915552923084 | 14 | 0.639066996442696 | 5.24053112078568 | 14 | 0.982175506407341 |
| Luxembourg | NetDrift = 0 | 0.614132179680396 | 1 | 0.433236200071878 | 2.32912791663793e-08 | 1 | 0.999878231002608 | 0.329582030992493 | 1 | 0.565905307068894 |
| Luxembourg | All Age Deviations = 0 | 232.039140275697 | 12 | 7.51233074126346e-43 | 207.703998579535 | 12 | 8.35131085781018e-38 | 430.299256954532 | 12 | 1.43346998637582e-84 |
| Luxembourg | All Period Deviations = 0 | 0.160272920821634 | 4 | 0.996955568684159 | 0.0841339769276266 | 4 | 0.99913961150057 | 0.243416086058278 | 4 | 0.993167964209764 |
| Luxembourg | All Cohort Deviations = 0 | 2.06856356234419 | 17 | 0.999995552629189 | 0.610075968717897 | 17 | 0.99999999973573 | 1.64796641585127 | 17 | 0.999999223959772 |
| Luxembourg | All Period RR = 1 | 0.763432267054166 | 5 | 0.979306882068343 | 0.0841878189209838 | 5 | 0.999893847196195 | 0.574587584422037 | 5 | 0.989137175116586 |
| Luxembourg | All Cohort RR = 1 | 3.16358413898968 | 18 | 0.99995840009922 | 0.634375624287138 | 18 | 0.999999999932658 | 1.71987299012018 | 18 | 0.999999672145576 |
| Luxembourg | All Local Drifts = Net Drift | 2.06342425716288 | 14 | 0.999899218534414 | 0.578603250821999 | 14 | 0.999999973859563 | 1.64000507642653 | 14 | 0.999975760804532 |
| Netherlands | NetDrift = 0 | 105.886399752947 | 1 | 7.80856991320705e-25 | 71.690557701554 | 1 | 2.5173428864029e-17 | 323.414739456957 | 1 | 2.61281815733385e-72 |
| Netherlands | All Age Deviations = 0 | 8598.90640269115 | 12 | 0 | 4558.5355351775 | 12 | 0 | 12665.1120280197 | 12 | 0 |
| Netherlands | All Period Deviations = 0 | 63.9140204452924 | 4 | 4.35707071818545e-13 | 33.4815417534313 | 4 | 9.51805198399729e-07 | 97.9591471740929 | 4 | 2.67444709769359e-20 |
| Netherlands | All Cohort Deviations = 0 | 59.6013550029002 | 17 | 1.22260651898262e-06 | 5.87543540086806 | 17 | 0.993984275714577 | 74.359763157087 | 17 | 3.78037396645115e-09 |
| Netherlands | All Period RR = 1 | 162.639784289152 | 5 | 2.70934617619876e-33 | 101.101244500868 | 5 | 3.09684353696816e-20 | 407.031156043623 | 5 | 9.05242139463588e-86 |
| Netherlands | All Cohort RR = 1 | 457.939281629178 | 18 | 7.0441074410185e-86 | 215.197652880221 | 18 | 8.96548378739979e-36 | 688.545034262674 | 18 | 1.52835518987046e-134 |
| Netherlands | All Local Drifts = Net Drift | 59.5264251689816 | 14 | 1.42018118333046e-07 | 5.80949689911842 | 14 | 0.971066875281126 | 74.3310499025719 | 14 | 3.13833971456744e-10 |
| Norway | NetDrift = 0 | 11.084438787894 | 1 | 0.000870551653618413 | 7.95516209707976 | 1 | 0.00479504141570037 | 23.541671561231 | 1 | 1.22237389200994e-06 |
| Norway | All Age Deviations = 0 | 1570.48922001519 | 12 | 0 | 1586.69481720218 | 12 | 0 | 2919.46370288963 | 12 | 0 |
| Norway | All Period Deviations = 0 | 2.22285396512925 | 4 | 0.694847439790128 | 2.05366327996987 | 4 | 0.725889242250297 | 4.18459006126836 | 4 | 0.381600325259893 |
| Norway | All Cohort Deviations = 0 | 15.5241467457088 | 17 | 0.557782757999213 | 17.2335382724821 | 17 | 0.438655549317493 | 49.2593757187063 | 17 | 5.50028569058878e-05 |
| Norway | All Period RR = 1 | 13.2929054275014 | 5 | 0.0207832093874308 | 10.4596773472096 | 5 | 0.0632102123882743 | 29.0568026891092 | 5 | 2.26001414194294e-05 |
| Norway | All Cohort RR = 1 | 70.391662300414 | 18 | 3.87930188378617e-08 | 26.6870800740311 | 18 | 0.0850546577465266 | 97.5243684339462 | 18 | 6.27839266307396e-13 |
| Norway | All Local Drifts = Net Drift | 15.5166420846675 | 14 | 0.343773889300316 | 17.231819570282 | 14 | 0.24403455967467 | 49.254598181973 | 14 | 8.13472521944947e-06 |
| Portugal | NetDrift = 0 | 19.5813804173955 | 1 | 9.64041574212055e-06 | 15.3957903228553 | 1 | 8.71823010783095e-05 | 49.0097968733602 | 1 | 2.54687245725316e-12 |
| Portugal | All Age Deviations = 0 | 5380.12707212567 | 12 | 0 | 2873.46420702539 | 12 | 0 | 6396.2672683251 | 12 | 0 |
| Portugal | All Period Deviations = 0 | 4.58251642413424 | 4 | 0.332874983265452 | 30.3525199830058 | 4 | 4.148701116512e-06 | 12.9566835205421 | 4 | 0.0114893968420387 |
| Portugal | All Cohort Deviations = 0 | 73.6790988278732 | 17 | 4.96964820099862e-09 | 68.5233286062101 | 17 | 3.86688645897383e-08 | 117.792128585786 | 17 | 4.05689301776942e-17 |
| Portugal | All Period RR = 1 | 25.3675680316399 | 5 | 0.000118311154919116 | 45.3681207230362 | 5 | 1.2211590115965e-08 | 61.0734483821065 | 5 | 7.29155127433118e-12 |
| Portugal | All Cohort RR = 1 | 74.4856085805952 | 18 | 7.75819302199866e-09 | 75.8932880820873 | 18 | 4.43713142422996e-09 | 124.01519722897 | 18 | 7.3000982538856e-18 |
| Portugal | All Local Drifts = Net Drift | 73.414290219926 | 14 | 4.61750411207816e-10 | 68.3741995088543 | 14 | 3.79632282011403e-09 | 117.603726632077 | 14 | 1.8515455199857e-18 |
| Spain | NetDrift = 0 | 61.8937074932199 | 1 | 3.62506108375565e-15 | 13.0688362851509 | 1 | 0.000300249617370521 | 10.3874080201294 | 1 | 0.00126877608309247 |
| Spain | All Age Deviations = 0 | 6740.87494603898 | 12 | 0 | 7099.28733509679 | 12 | 0 | 8131.2188097797 | 12 | 0 |
| Spain | All Period Deviations = 0 | 59.7757619343916 | 4 | 3.23330247828219e-12 | 83.126150582942 | 4 | 3.78807657259986e-17 | 87.640238382382 | 4 | 4.17481318169199e-18 |
| Spain | All Cohort Deviations = 0 | 102.795211396256 | 17 | 2.69202361053059e-14 | 119.717312566263 | 17 | 1.74571916629507e-17 | 143.760388328307 | 17 | 4.04856549875288e-22 |
| Spain | All Period RR = 1 | 132.358340456041 | 5 | 7.51628461049555e-27 | 91.573820111524 | 5 | 3.13777534730576e-18 | 106.009465154716 | 5 | 2.85367269578013e-21 |
| Spain | All Cohort RR = 1 | 180.951172026466 | 18 | 6.21429002401317e-29 | 294.388495348135 | 18 | 6.85566481993555e-52 | 144.786492995954 | 18 | 7.6229791767965e-22 |
| Spain | All Local Drifts = Net Drift | 102.559989579509 | 14 | 1.52956475099103e-15 | 119.152887766503 | 14 | 9.21771531961361e-19 | 143.555718872883 | 14 | 1.3906253864751e-23 |
| Sweden | NetDrift = 0 | 37.1308430884551 | 1 | 1.10462622691167e-09 | 72.084858707192 | 1 | 2.06139393829754e-17 | 149.654263020523 | 1 | 2.06314698882967e-34 |
| Sweden | All Age Deviations = 0 | 7647.0820485331 | 12 | 0 | 5256.27247915494 | 12 | 0 | 10372.6831281627 | 12 | 0 |
| Sweden | All Period Deviations = 0 | 17.8184262293235 | 4 | 0.00133911253380436 | 41.6800286200292 | 4 | 1.94334407677127e-08 | 24.1481726946878 | 4 | 7.45937214125831e-05 |
| Sweden | All Cohort Deviations = 0 | 305.931589383699 | 17 | 6.71034023743937e-55 | 259.957337680152 | 17 | 1.92055226462413e-45 | 538.211059289124 | 17 | 1.65351254504043e-103 |
| Sweden | All Period RR = 1 | 56.6025462899652 | 5 | 6.10652844859032e-11 | 105.090275953322 | 5 | 4.46104748541427e-21 | 163.251597289074 | 5 | 2.00644441907271e-33 |
| Sweden | All Cohort RR = 1 | 404.520439920864 | 18 | 1.04400205348989e-74 | 499.784111549056 | 18 | 1.15832425728668e-94 | 799.156771129898 | 18 | 4.8014031828248e-158 |
| Sweden | All Local Drifts = Net Drift | 304.692914675984 | 14 | 1.24098374144113e-56 | 258.062521480012 | 14 | 6.16286743242788e-47 | 536.639814338524 | 14 | 1.56499832005901e-105 |
| UK | NetDrift = 0 | 41.7496825736685 | 1 | 1.03739091798185e-10 | 86.2900664756782 | 1 | 1.55387094793283e-20 | 157.989389709433 | 1 | 3.1114250198747e-36 |
| UK | All Age Deviations = 0 | 21627.0849794394 | 12 | 0 | 18385.2554743019 | 12 | 0 | 30809.6670014016 | 12 | 0 |
| UK | All Period Deviations = 0 | 165.30486041226 | 4 | 1.06410432375412e-34 | 103.720964811395 | 4 | 1.58638934607392e-21 | 212.375513169778 | 4 | 8.19197184167142e-45 |
| UK | All Cohort Deviations = 0 | 125.517178414971 | 17 | 1.36111487707597e-18 | 103.545640337195 | 17 | 1.95121972881445e-14 | 251.874508207893 | 17 | 8.64063883182835e-44 |
| UK | All Period RR = 1 | 193.597796245769 | 5 | 6.64743965832736e-40 | 179.148422381246 | 5 | 8.13401145991458e-37 | 342.256473176703 | 5 | 8.13015111042645e-72 |
| UK | All Cohort RR = 1 | 297.962007665377 | 18 | 1.26385386779762e-52 | 188.175956080305 | 18 | 2.28558512541155e-30 | 460.603776623352 | 18 | 1.94672629688898e-86 |
| UK | All Local Drifts = Net Drift | 125.492068172371 | 14 | 5.25776052359913e-20 | 103.544162978499 | 14 | 9.89058135620346e-16 | 251.854570553034 | 14 | 1.18818816317297e-45 |
| USA | NetDrift = 0 | 23.7146761853566 | 1 | 1.11727273070418e-06 | 25.1721232419764 | 1 | 5.24346727887479e-07 | 23.4312304793501 | 1 | 1.29459666471468e-06 |
| USA | All Age Deviations = 0 | 21278.5245490401 | 12 | 0 | 4448.14240512309 | 12 | 0 | 9739.32428356409 | 12 | 0 |
| USA | All Period Deviations = 0 | 58.682478967528 | 4 | 5.48649412355107e-12 | 11.9624819700683 | 4 | 0.0176324505650848 | 16.3447231068474 | 4 | 0.00258982034653647 |
| USA | All Cohort Deviations = 0 | 223.323713994654 | 17 | 5.59584846143599e-38 | 58.1217212103892 | 17 | 2.13823773381994e-06 | 99.4220813978437 | 17 | 1.13832822077267e-13 |
| USA | All Period RR = 1 | 75.88065698 | 5 | 6.09248431683013e-15 | 35.6265479150218 | 5 | 1.12799242812857e-06 | 36.1192127583211 | 5 | 8.99058950627576e-07 |
| USA | All Cohort RR = 1 | 357.129506962005 | 18 | 7.56740126335277e-65 | 215.219447995214 | 18 | 8.87542963529879e-36 | 279.836776123725 | 18 | 6.62318608132191e-49 |
| USA | All Local Drifts = Net Drift | 221.026998238865 | 14 | 2.70250041708651e-39 | 57.1585565525848 | 14 | 3.6719126848423e-07 | 98.0840575326212 | 14 | 1.10340652534733e-14 |

The Wald Tests follow a Chi-Square distribution when the Null Hypothesis is true. The df (degrees of freedom) count the number of free parameters included in each test. The web tool reports P-values; values less than 0.05 are often considered "statistically significant", meaning there is statistical evidence that the Null Hypothesis is unlikely to be correct.
