# Supplementary material for: Temporal Trend Analysis of Atrial Fibrillation/Flutter Disease Burden in High-Income Countries Between 1990 and 2021
Source: Rev Cardiovasc Med. 2025 Jul 25;26(7):36427. doi: 10.31083/RCM36427 (PMC12326443; doi:10.31083/RCM36427)
Supplement: Supplementary file 1 [file 2153-8174-26-7-36427-s1.zip › Supplementary Material 1.docx]

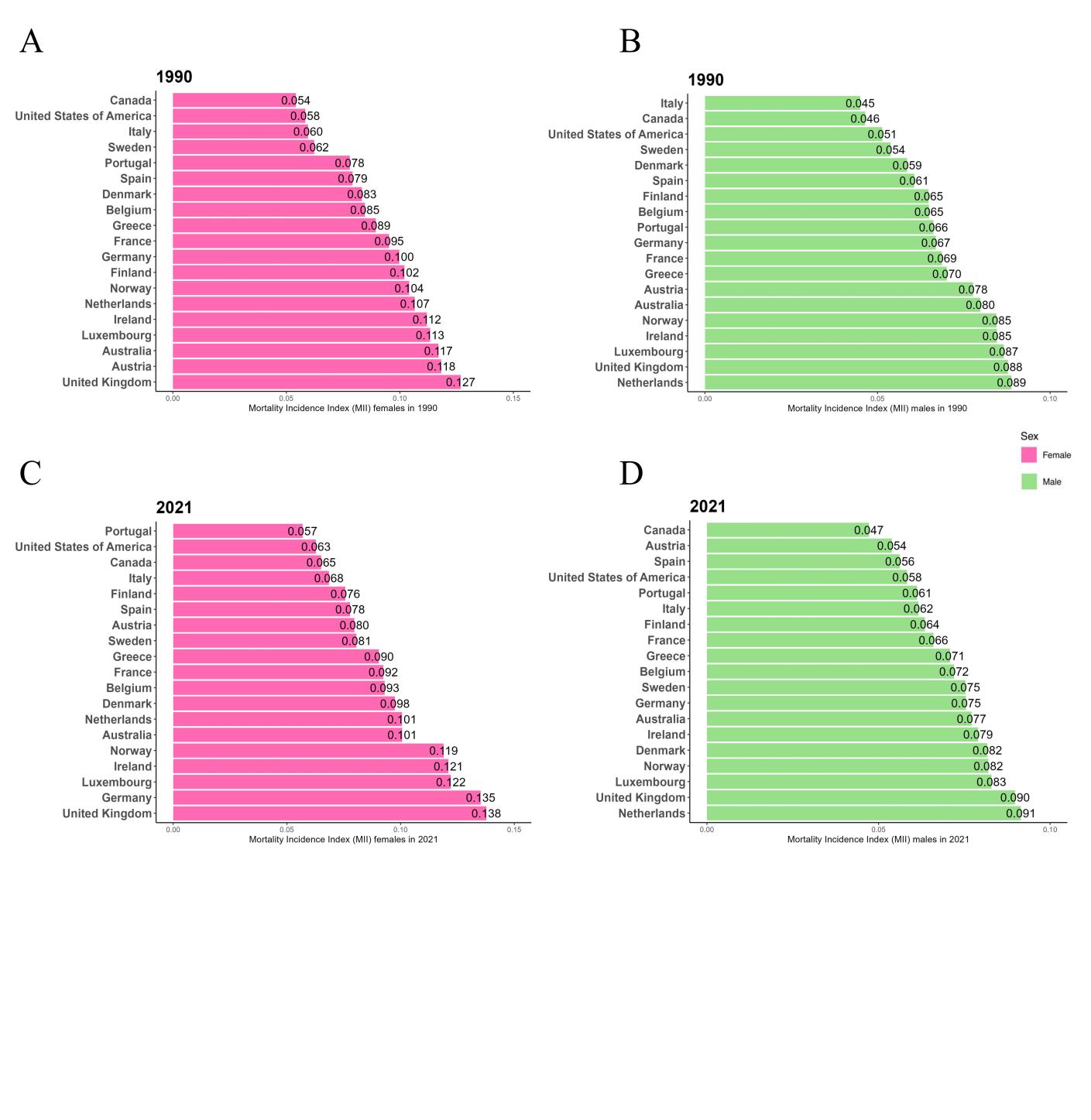


**Supplementary Fig. 1. the MII (Mortality-to-Incidence Ratio) for AF/AFL in 1990 and 2021in EU15+ countries.** (A) Female MII in 1990. (B) Male MII in 1990.(C)Female MII in 2021.(D)Male MII in 2021.


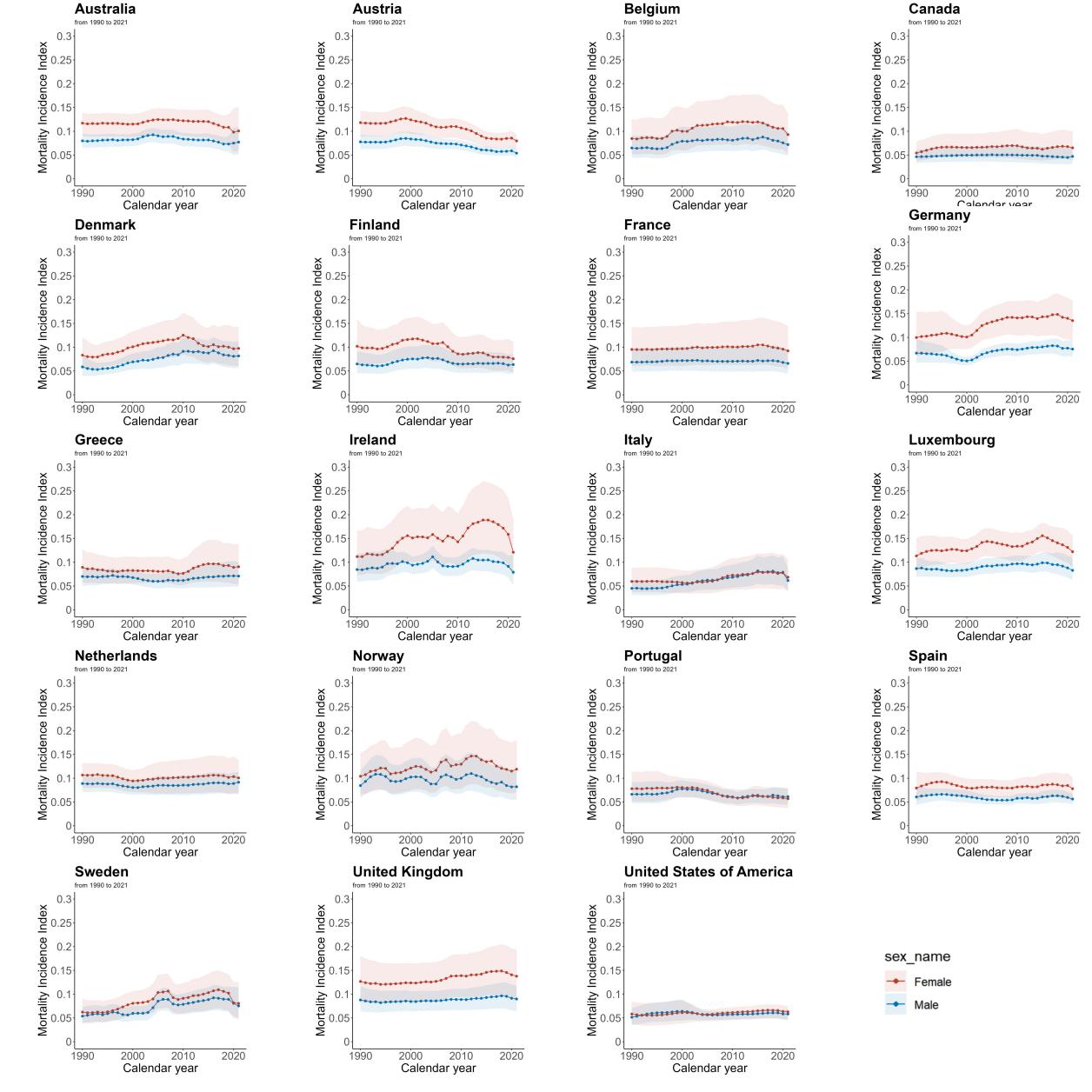


**Supplementary Fig. 2. Trends in the MII (Mortality-to-Incidence Ratio) for AF/AFL in EU15+ countries between 1990 and 2021**.Blue squares indicate males and red squares indicate females.

| **Supplementary Table 1. Joinpoint analysis for Atrial fibrillation/flutter disease mortality incidence index (MII) in EU15+ countries for years 1990–2021 in males.** | **AAPC** | **AAPC(95%CI)** | -0.15  (-0.68to0.39) | -1.06  (-1.66to-0.45)* | | 0.36  (-0.21to0.92) | | 0.03  (-0.09to0.14) | | 1.02  (0.32to1.72) | | -0.07  (-0.49to0.37) | | -0.12  (-0.30to0.06) | | 0.37  (-0.11to0.86) | | 0.11  (-0.22to0.45) | | 0.27  (-0.97to1.52) | | 1.15  (0.60to1.71)* | | -0.08  (-0.52to0.36) | | 0.04(  -0.28to0.37) | | -0.21  (-1.88to1.49) | | -0.21  (-0.77to0.34) | | -0.25  (-0.68to0.19) | | 0.94  (-0.70to2.60) | | 0.09  (-0.19to0.36) | | 0.41  (0.21to0.60)* | | *Significantly diﬀerent from 0 (P < 0.001).  CI, confidence interval; APC, annual percentage change. | |  |
| --- | --- | --- | --- | --- | --- | --- | --- | --- | --- | --- | --- | --- | --- | --- | --- | --- | --- | --- | --- | --- | --- | --- | --- | --- | --- | --- | --- | --- | --- | --- | --- | --- | --- | --- | --- | --- | --- | --- | --- | --- | --- | --- |
|  |  | **Year** | 1990-2021 | 1990-2021 | | 1990-2021 | | 1990-2021 | | 1990-2021 | | 1990-2021 | | 1990-2021 | | 1990-2021 | | 1990-2021 | | 1990-2021 | | 1990-2021 | | 1990-2021 | | 1990-2021 | | 1990-2021 | | 1990-2021 | | 1990-2021 | | 1990-2021 | | 1990-2021 | | 1990-2021 | |  |  |  |
|  |  |  |  |  | |  | |  | |  | |  | |  | |  | |  | |  | |  | |  | |  | |  | |  | |  | |  | |  | |  | |  |  |  |
|  | **Trend6** | **APC(95%CI)** | 1.19  (-1.59to4.05) | -1.30  (-1.96to-0.64)* | | -3.54  (-4.39to-2.68)* | | 1.62  (0.51to2.73)* | | -2.23  (-3.53to-0.90)* | | -1.69  (-3.29to-0.06)* | | -2.73  (-3.47to-1.98)* | | -2.05  (-3.27to-0.81)* | | 0.62  (0.11to1.14)* | | -2.68  (-3.89to-1.46)* | | -11.15  (-15.01to-7.12)* | | -3.14  (-3.99to-2.29)* | | 0.03(-0.61to0.67) | | -3.38  (-4.54to-2.20)* | | -0.26  (-0.80to0.29) | | -4.09  (-5.67to-2.48)* | | -8.25  (-19.68to4.81) | | -2.34  (-3.37to-1.30)* | | -1.54  (-2.18to-0.89)* | |  |  |  |
|  |  | **Year** | 2018-2021 | 2014-2021 | | 2016-2021 | | 2019-2021 | | 2016-2021 | | 2018-2021 | | 2018-2021 | | 2017-2021 | | 2014-2021 | | 2014-2021 | | 2019-2021 | | 2016-2021 | | 2016-2021 | | 2012-2021 | | 2014-2021 | | 2018-2021 | | 2019-2021 | | 2018-2021 | | 2018-2021 | |  |  |  |
|  |  |  |  |  | |  | |  | |  | |  | |  | |  | |  | |  | |  | |  | |  | |  | |  | |  | |  | |  | |  | |  |  |  |
|  | **Trend5** | **APC(95%CI)** | -3.64  (-6.95to-0.21)* | -4.10  (-6.35to-1.79)* | | 1.11  (0.25to1.97)* | | -1.61  (-1.95to-1.28)* | | -0.30  (-1.99to1.43) | | 0.16  (-0.21to0.53) | | 0.28  (0.15to0.42)* | | 1.07  (0.76to1.38)* | | 2.44  (0.86to4.05)* | | 3.62  (1.85to5.42)* | | 0.67  (-1.44to2.82) | | 1.62  (-1.50to4.85) | | 0.89  (0.40to1.39)* | | 2.11  (0.38to3.86)* | | 2.46  (-1.19to6.25) | | 2.48  (0.73to4.27)* | | -1.04  (-9.97to8.77) | | 1.23  (0.90to1.56)* | | 1.06  (0.45to1.69)* | |  |  |  |
|  |  | **Year** | 2015-2018 | 2010-2014 | | 2010-2016 | | 2014-2019 | | 2011-2016 | | 2009-2018 | | 2008-2018 | | 2007-2017 | | 2010-2014 | | 2008-2014 | | 2015-2019 | | 2013-2016 | | 2009-2016 | | 2004-2012 | | 2011-2014 | | 2014-2018 | | 2016-2019 | | 2011-2018 | | 2014-2018 | |  |  |  |
|  |  |  |  |  | |  | |  | |  | |  | |  | |  | |  | |  | |  | |  | |  | |  | |  | |  | |  | |  | |  | |  |  |  |
|  | **Trend4** | **APC(95%CI)** | 0.05  (-3.10to3.30) | -0.80  (-3.06to1.52) | | -0.64  (-4.26to3.13) | | -0.50  (-0.82to-0.18)* | | 2.62  (2.28to2.96)* | | -5.68  (-8.74to-2.51)* | | -0.61  (-1.06to-0.16)* | | 2.35  (-1.00to5.81) | | 0.67  (0.06to1.29)* | | -6.83  (-13.71to0.60) | | 2.89  (2.33to3.46)* | | -0.73  (-3.40to2.01) | | -0.41  (-3.06to2.31) | | -4.31  (-15.70to8.63) | | -3.11  (-3.56to-2.65)* | | 0.03  (-3.02to3.17) | | 2.63  (1.03to4.26)* | | -0.09  (-1.87to1.73) | | 0.51  (0.37to0.64)* | |  |  |  |
|  |  | **Year** | 2012-2015 | 2006-2010 | | 2007-2010 | | 2009-2014 | | 1999-2011 | | 2006-2009 | | 2003-2008 | | 2004-2007 | | 2004-2010 | | 2005-2008 | | 2007-2015 | | 2010-2013 | | 2006-2009 | | 2001-2004 | | 2003-2011 | | 2011-2014 | | 2009-2016 | | 2008-2011 | | 2005-2014 | |  |  |  |
|  |  |  |  |  | |  | |  | |  | |  | |  | |  | |  | |  | |  | |  | |  | |  | |  | |  | |  | |  | |  | |  |  |  |
|  | **Trend3** | **APC(95%CI)** | -1.56  (-1.96to-1.15)* | -2.06  (-2.98to-1.13)* | | 0.90  (0.38to1.42)* | | -0.10  (-0.58to0.37) | | 5.67  (1.28to10.26)* | | 0.66  (0.11to1.21)* | | 0.19  (-0.12to0.51) | | 8.58  (6.88to10.32)* | | -2.94  (-3.64to-2.25)* | | 5.09  (-2.43to13.18) | | 1.12  (-2.79to5.18) | | 0.93  (0.47to1.39)* | | 1.09  (0.53to1.66)* | | 3.27  (-2.77to9.68) | | -1.24  (-4.54to2.18) | | 2.45(  1.03to3.89)* | | -6.37  (-14.32to2.32) | | 1.07  (-0.70to2.86) | | -2.85  (-3.38to-2.31)* | |  |  |  |
|  |  | **Year** | 2004-2012 | 1999-2006 | | 1999-2007 | | 2005-2009 | | 1996-1999 | | 1999-2006 | | 1997-2003 | | 2000-2004 | | 1999-2004 | | 2002-2005 | | 2004-2007 | | 2003-2010 | | 2000-2006 | | 1997-2001 | | 2000-2003 | | 2007-2011 | | 2006-2009 | | 2005-2008 | | 2000-2005 | |  |  |  |
|  |  |  |  |  | |  | |  | |  | |  | |  | |  | |  | |  | |  | |  | |  | |  | |  | |  | |  | |  | |  | |  |  |  |
|  | **Trend2** | **APC(95%CI)** | 3.03  (1.53to4.54)* | 2.78  (-0.22to5.86) | | 7.13  (2.78to11.67)* | | 0.29  (0.09to0.49)* | | 1.60  (-0.59to3.83) | | 5.23  (3.47to7.01)* | | 1.06  (-0.36to2.49) | | -5.02  (-6.19to-3.83)* | | -0.67(  -2.79to1.49) | | -2.30  (-9.02to4.92) | | 3.46  (3.02to3.90)* | | 2.63  (1.28to3.99)* | | -2.31  (-3.37to-1.23)* | | -4.57  (-10.13to1.33) | | 3.76  (1.67to5.91)* | | -1.98  (-2.17to-1.78)* | | 15.00  (5.60to25.23)* | | 0.30  (0.17to0.42)* | | 1.38  (0.94to1.82)* | |  |  |  |
|  |  | **Year** | 2000-2004 | 1995-1999 | | 1996-1999 | | 1999-2005 | | 1992-1996 | | 1995-1999 | | 1994-1997 | | 1995-2000 | | 1996-1999 | | 1999-2002 | | 1995-2004 | | 1999-2003 | | 1996-2000 | | 1993-1997 | | 1996-2000 | | 1995-2007 | | 2003-2006 | | 1993-2005 | | 1993-2000 | |  |  |  |
|  |  |  |  |  | |  | |  | |  | |  | |  | |  | |  | |  | |  | |  | |  | |  | |  | |  | |  | |  | |  | |  |  |  |
|  | **Trend1** | **APC(95%CI)** | 0.35  (0.11to0.59)* | -0.04  (-1.42to1.37) | | -0.57  (-1.28to0.14) | | 0.77  (0.69to0.85)* | | -4.74  (-10.75to1.68) | | -1.15  (-1.93to-0.35)* | | 0.15  (-0.29to0.59) | | -0.99  (-2.38to0.42) | | 0.40(-  0.02to0.83) | | 2.17  (1.19to3.16)* | | -0.06  (-0.93to0.83) | | -0.68  (-0.95to-0.40)* | | -0.16  (-0.53to0.22) | | 9.39  (3.02to16.15)* | | 0.35  (-0.56to1.27) | | 1.87  (0.96to2.79)* | | 0.68  (0.19to1.18)* | | -1.73  (-2.75to-0.69)* | | 4.54  (3.18to5.92)* | |  |  |  |
|  |  | **Year** | 1990-2000 | 1990-1995 | | 1990-1996 | | 1990-1999 | | 1990-1992 | | 1990-1995 | | 1990-1994 | | 1990-1995 | | 1990-1996 | | 1990-1999 | | 1990-1995 | | 1990-1999 | | 1990-1996 | | 1990-1993 | | 1990-1996 | | 1990-1995 | | 1990-2003 | | 1990-1993 | | 1990-1993 | |  |  |  |
|  |  | **Country** | **Australia** | **Austria** | | **Belgium** | | **Canada** | | **Denmark** | | **Finland** | | **France** | | **Germany** | | **Greece** | | **Ireland** | | **Italy** | | **Luxembourg** | | **Netherlands** | | **Norway** | | **Portugal** | | **Spain** | | **Sweden** | | **UK** | | **USA** | |  |  |  |
| **Supplementary Table 2. Joinpoint analysis for Atrial fibrillation/flutter disease mortality incidence index (MII) in EU15+ countries for years 1990–2021 in females.** | **AAPC** | **AAPC(95%CI)** | -0.51(-0.94to-o.o9) | | -1.15(-1.61 to -0.70)* | | 0.37(-0.30 to 1.05) | | 0.60(0.35 to 0.86) | | 0.54(-0.13 to 1.21)* | | -0.92(-1.48 to -0.36) | | -0.07(-0.28 to 0.15) | | 1.00(0.58 to 1.41) | | 0.06(-0.35 to 0.47) | | 0.37(-0.58 to 1.33) | | 0.55(-0.18 to 1.28)* | | 0.29(-0.06 to 0.64) | | -0.17(-0.37 to 0.02) | | 0.27(-1.49 to 2.07) | | -1.02(-1.33 to -0.71) | | -0.08(-0.58 to 0.44) | | 0.78(-0.30 to 1.87) | | 0.28(0.03 to 0.53) | | 0.29(0.08 to 0.50)* | | *Significantly diﬀerent from 0 (P < 0.001).  CI, confidence interval; APC, annual percentage change. | |
|  |  | **Year** | 1990-2021 | | 1990-2021 | | 1990-2021 | | 1990-2021 | | 1990-2021 | | 1990-2021 | | 1990-2021 | | 1990-2021 | | 1990-2021 | | 1990-2021 | | 1990-2021 | | 1990-2021 | | 1990-2021 | | 1990-2021 | | 1990-2021 | | 1990-2021 | | 1990-2021 | | 1990-2021 | | 1990-2021 | |  |  |
|  |  |  |  | |  | |  | |  | |  | |  | |  | |  | |  | |  | |  | |  | |  | |  | |  | |  | |  | |  | |  | |  |  |
|  | **Trend6** | **APC(95%CI)** | -3.10(-3.76to-2.45)* | | -0.68(-1.39to0.05) | | -5.64(-10.28to-0.77)* | | -2.67(-5.02to-0.27)* | | -0.83(-1.56to-0.09)* | | -2.16(-2.78to-1.54)* | | -2.43(-2.74to-2.13)* | | -2.35(-4.04to-0.62)* | | -1.10(-1.57to-0.63)* | | -16.07(-22.50to-9.10)* | | -4.20(-6.77to-1.56)* | | -3.67(-4.21to-3.13)* | | -1.32(-1.96to-0.68)* | | -3.07(-4.40to-1.73)* | | -2.03(-2.39to-1.66)* | | -3.11(-5.53to-0.62)* | | -10.75(-14.68to-6.64)* | | -2.83(-3.78to-1.86)* | | -1.29(-1.72to-0.86)* | |  |  |
|  |  | **Year** | 2015-2021 | | 2015-2021 | | 2019-2021 | | 2019-2021 | | 2014-2021 | | 2014-2021 | | 2016-2021 | | 2018-2021 | | 2014-2021 | | 2019-2021 | | 2018-2021 | | 2015-2021 | | 2017-2021 | | 2013-2021 | | 2015-2021 | | 2018-2021 | | 2018-2021 | | 2018-2021 | | 2017-2021 | |  |  |
|  |  |  |  | |  | |  | |  | |  | |  | |  | |  | |  | |  | |  | |  | |  | |  | |  | |  | |  | |  | |  | |  |  |
|  | **Trend5** | **APC(95%CI)** | -0.13(-2.93to2.75) | | -4.78(-6.01to-3.54)* | | -2.91(-5.29to-0.47)* | | 2.56(1.35to3.79)* | | -5.50(-10.12to-0.63)* | | 0.75(-1.47to3.03) | | 1.17(-0.23to2.58) | | 0.39(0.16to0.62)* | | 6.51(4.92to8.13)* | | -1.26(-3.40to0.92) | | 1.36(0.79to1.94)* | | 4.38(2.95to5.83)* | | 0.55(0.42to0.69)* | | 2.33(0.97to3.71)* | | 3.00(1.00to5.04)* | | 0.98(0.59to1.38)* | | 2.38(1.54to3.22)* | | 1.11(0.81to1.42)* | | 1.17(1.03to1.32)* | |  |  |
|  |  | **Year** | 2012-2015 | | 2010-2015 | | 2015-2019 | | 2015-2019 | | 2011-2014 | | 2010-2014 | | 2013-2016 | | 2007-2018 | | 2010-2014 | | 2014-2019 | | 2009-2018 | | 2011-2015 | | 2005-2017 | | 2004-2013 | | 2012-2015 | | 2008-2018 | | 2009-2018 | | 2011-2018 | | 2008-2017 | |  |  |
|  |  |  |  | |  | |  | |  | |  | |  | |  | |  | |  | |  | |  | |  | |  | |  | |  | |  | |  | |  | |  | |  |  |
|  | **Trend4** | **APC(95%CI)** | -0.67(-1.99to0.66) | | 0.32(-1.66to2.34) | | 0.41(-0.11to0.94) | | -1.88(-2.40to-1.35)* | | 1.60(1.17to2.03)* | | -7.52(-11.44to-3.44)* | | 0.29(0.06to0.52)* | | 2.95(1.56to4.36)* | | -2.04(-4.66to0.65)* | | 6.33(2.86to9.91)* | | 5.95(0.73to11.44)* | | -1.23(-1.74to-0.72)* | | 1.19(0.68to1.70)* | | -1.94(-12.84to10.32) | | -1.00(-2.88to0.92) | | -0.86(-4.43to2.86) | | -6.13(-12.60to0.82) | | 0.23(-1.47to1.95) | | 1.63(0.32to2.95)* | |  |  |
|  |  | **Year** | 2008-2012 | | 2006-2010 | | 2006-2015 | | 2009-2015 | | 2001-2011 | | 2007-2010 | | 2006-2013 | | 2003-2007 | | 2007-2010 | | 2010-2014 | | 2006-2009 | | 2004-2011 | | 2000-2005 | | 2001-2004 | | 2009-2012 | | 2005-2008 | | 2006-2009 | | 2008-2011 | | 2005-2008 | |  |  |
|  |  |  |  | |  | |  | |  | |  | |  | |  | |  | |  | |  | |  | |  | |  | |  | |  | |  | |  | |  | |  | |  |  |
|  | **Trend3** | **APC(95%CI)** | -0.06(-1.37to1.27) | | -2.30(-3.02to-1.58)* | | 2.06(1.23to2.90)* | | 0.92(0.44to1.41)* | | 4.03(1.82to6.28)* | | -1.32(-2.01to-0.61)* | | -0.02(-1.32to1.29) | | 7.78(4.91to10.72)* | | -0.32(-0.67to0.04)* | | -0.32(-0.82to0.18) | | 1.28(-0.27to2.86) | | 3.61(2.11to5.14)* | | -2.80(-3.46to-2.13)* | | 3.56(-2.40to9.89) | | -5.03(-5.89to-4.17)* | | 0.50(-0.72to1.74) | | 7.46(0.01to15.46)* | | 2.88(1.19to4.60)* | | -1.52(-2.06to-0.98)* | |  |  |
|  |  | **Year** | 2004-2008 | | 1999-2006 | | 1999-2006 | | 2003-2009 | | 1997-2001 | | 2000-2007 | | 2003-2006 | | 2000-2003 | | 1999-2007 | | 1999-2010 | | 2001-2006 | | 2000-2004 | | 1996-2000 | | 1997-2001 | | 2005-2009 | | 2000-2005 | | 2003-2006 | | 2005-2008 | | 2000-2005 | |  |  |
|  |  |  |  | |  | |  | |  | |  | |  | |  | |  | |  | |  | |  | |  | |  | |  | |  | |  | |  | |  | |  | |  |  |
|  | **Trend2** | **APC(95%CI)** | 2.49(-0.06to5.11) | | 1.94(0.46to3.45)* | | 5.39(0.17to10.88)* | | 0.00(-0.22to0.22) | | 2.68(1.32to4.06)* | | 4.09(2.76to5.45)* | | 1.00(-0.30to2.32) | | -2.29(-3.80to-0.76)* | | 0.90(-1.74to3.61)* | | 8.83(1.87to16.26)* | | -1.61(-6.16to3.16) | | -0.10(-0.64to0.43) | | -0.65(-1.97to0.70) | | -3.73(-14.58to8.51) | | -2.74(-4.37to-1.08)* | | -3.40(-4.54to-2.24)* | | 3.86(3.05to4.67)* | | 0.43(0.30to0.56)* | | 2.02(1.46to2.60)* | |  |  |
|  |  | **Year** | 2001-2004 | | 1994-1999 | | 1996-1999 | | 1994-2003 | | 1992-1997 | | 1995-2000 | | 2000-2003 | | 1996-2000 | | 1996-1999 | | 1996-1999 | | 1998-2001 | | 1993-2000 | | 1993-1996 | | 1994-1997 | | 2002-2005 | | 1995-2000 | | 1994-2003 | | 1994-2005 | | 1994-2000 | |  |  |
|  |  |  |  | |  | |  | |  | |  | |  | |  | |  | |  | |  | |  | |  | |  | |  | |  | |  | |  | |  | |  | |  |  |
|  | **Trend1** | **APC(95%CI)** | -0.10(-0.26to0.07) | | -0.49(-1.98to1.03) | | 0.20(-0.64to1.05) | | 5.08(4.43to5.74)* | | -2.73(-7.79to2.60) | | -0.74(-1.79to0.33) | | 0.19(0.09to0.28)* | | 1.48(0.67to2.30)* | | -1.59(-2.06to-1.12)* | | 1.10(-0.09to2.31) | | -0.16(-0.69to0.37) | | 3.23(1.18to5.33)* | | 0.23(-0.45to0.92) | | 4.05(0.29to7.94)* | | 0.30(0.17to0.43)* | | 2.99(1.88to4.10)* | | 0.11(-2.46to2.75) | | -1.11(-1.71to-0.50)* | | -1.38(-2.17to-0.60)* | |  |  |
|  |  | **Year** | 1990-2001 | | 1990-1994 | | 1990-1996 | | 1990-1994 | | 1990-1992 | | 1990-1995 | | 1990-2000 | | 1990-1996 | | 1990-1996 | | 1990-1996 | | 1990-1998 | | 1990-1993 | | 1990-1993 | | 1990-1994 | | 1990-2002 | | 1990-1995 | | 1990-1994 | | 1990-1994 | | 1990-1994 | |  |  |
|  |  | **Country** | **Australia** | | **Austria** | | **Belgium** | | **Canada** | | **Denmark** | | **Finland** | | **France** | | **Germany** | | **Greece** | | **Ireland** | | **Italy** | | **Luxembourg** | | **Netherlands** | | **Norway** | | **Portugal** | | **Spain** | | **Sweden** | | **UK** | | **USA** | |  |  |
